# Visualization of Genome Signatures of Eukaryote Genomes by Batch-Learning Self-Organizing Map with a Special Emphasis on *Drosophila* Genomes

**DOI:** 10.1155/2014/985706

**Published:** 2014-03-11

**Authors:** Takashi Abe, Yuta Hamano, Toshimichi Ikemura

**Affiliations:** ^1^Information Engineering, Niigata University, Niigata-shi, Niigata-ken 950-2181, Japan; ^2^Graduate School of Information Science, Nara Institute of Science and Technology, Ikoma-shi, Nara-ken 630-0101, Japan; ^3^Nagahama Institute of Bio-Science and Technology, Nagahama-shi, Shiga-ken 526-0829, Japan

## Abstract

A strategy of evolutionary studies that can compare vast numbers of genome sequences is becoming increasingly important with the remarkable progress of high-throughput DNA sequencing methods. We previously established a sequence alignment-free clustering method “BLSOM” for di-, tri-, and tetranucleotide compositions in genome sequences, which can characterize sequence characteristics (genome signatures) of a wide range of species. In the present study, we generated BLSOMs for tetra- and pentanucleotide compositions in approximately one million sequence fragments derived from 101 eukaryotes, for which almost complete genome sequences were available. BLSOM recognized phylotype-specific characteristics (e.g., key combinations of oligonucleotide frequencies) in the genome sequences, permitting phylotype-specific clustering of the sequences without any information regarding the species. In our detailed examination of 12 *Drosophila* species, the correlation between their phylogenetic classification and the classification on the BLSOMs was observed to visualize oligonucleotides diagnostic for species-specific clustering.

## 1. Introduction

Genome sequences, even protein-noncoding sequences, contain a wealth of information. The G + C content (%GC) is a fundamental characteristic of individual genomes and is used for a long period as a basic phylogenetic parameter to characterize individual genomes and genomic portions. The %GC, however, is too simple a parameter to differentiate wide varieties of genomes. Many groups have reported that oligonucleotide composition varies significantly among genomes and can be used to study genome diversity [1–8]. Because oligonucleotide composition can be used to distinguish species even with the same %GC, it has been called a “genome signature” [4, 5].

The unsupervised neural network algorithm known as Kohonen's Self-Organizing Map (SOM) is a powerful tool for clustering and visualizing high-dimensional vectorial data on a two-dimensional map [9–11]; oligonucleotide composition is an example of such high-dimensional data. We have previously developed a modified type SOM (batch-learning SOM: BLSOM) that depends on neither the order of data input nor the initial conditions, for codon frequencies in gene sequences [12] and oligonucleotide frequencies in genome sequences [13, 14]. BLSOM could recognize and visualize species-specific characteristics of codon or oligonucleotide frequencies in individual genomes, permitting clustering of genes or genome fragments according to species without the need for species information during BLSOM learning. Various high-performance supercomputers are now available for biological studies, and the BLSOM is suitable for actualizing high-performance parallel-computing with high-performance supercomputers. Therefore, this alignment-free clustering method was successfully applied to the phylogenetic classification of genome sequence fragments [15] and to the analysis of a large number of microbial sequences obtained by metagenome studies of environmental and clinical samples [16].

To test the power of BLSOM to detect differences among eukaryote genomes and particularly among closely related species, the present study examined* Drosophila* in detail, for which the genomes of many closely related species have been sequenced. We constructed BLSOM with tetra- and pentanucleotide compositions in most (if not all) of the* Drosophila* genomes available and focused on species-specific characteristics of oligonucleotide frequencies in each* Drosophila* genome (genome signature), in connection with their phylogenetic classification.

## 2. Materials and Methods

### 2.1. Batch-Learning Self-Organizing Map (BLSOM)

SOM is an unsupervised neural network algorithm that implements a characteristic nonlinear projection from the high-dimensional space of input data onto a two-dimensional array of weight vectors [9–11]. We modified the conventional SOM for genome informatics to make the learning process and resulting map independent of the order of data input, on the basis of batch learning SOM (BLSOM) [12, 13]. The initial weight vectors were defined by Principal Component Analysis (PCA) instead of random values. BLSOM learning was conducted as described previously [13], and the BLSOM program was obtained from UNTROD Inc. (takaabe@ie.niigata-u.ac.jp or y_wada@nagahama-i-bio.ac.jp).

### 2.2. Genome Sequences

Genome DNA sequences were obtained from http://hgdownload.cse.ucsc.edu/downloads.html. When the number of undetermined nucleotides (Ns) in a sequence exceeded 10% of the window size, the sequence was omitted from the analysis. When the number of Ns was less than 10%, the oligonucleotide frequencies were normalized to the length without Ns and included in the analysis.

## 3. Results

### 3.1. BLSOMs for 101 Eukaryote Genomes

**Figure 1:**
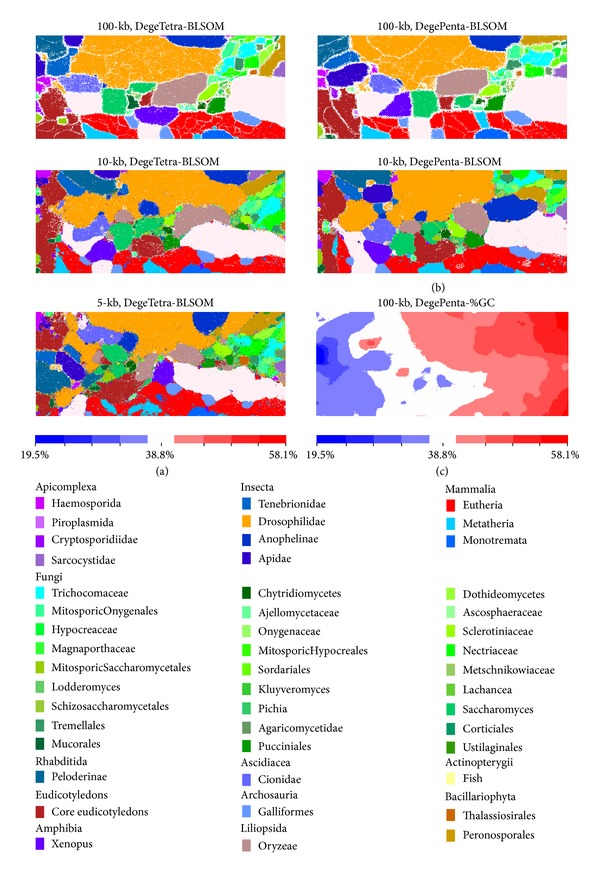
BLSOMs for the overlapping 100 kb with a 10 kb sliding step and the nonoverlapping 10- and 5 kb sequences from 101 eukaryotic genomes. (a) DegeTetra- and (b) DegePenta-BLSOMs. BLSOM was constructed with frequencies for degenerate sets in which the frequencies of a pair of complimentary tetra- or pentanucleotides were added. Nodes that include sequences from more than one phylogenetic family are indicated in black, those that contain no genomic sequences are indicated in white, and those containing sequences from a single family are indicated in colors. Differences in color were difficult to distinguish individual phylogenetic families because 47 families were analyzed simultaneously, but the observation that the back nodes were very rare showed the proper clustering of sequences according to phylotype. (c) 100 kb DegePenta-%GC; the %GC was calculated from the vectorial data representing each node in the 100 kb DegePenta-BLSOM and divided into nine categories with an equal number of nodes, as listed at the bottom of this panel; the %GC ranged from 19.5 to 58.1 and the midvalue was 38.8.

A large number of genomes, including a wide variety of eukaryotes, have been sequenced with the remarkable progress of currently available DNA sequencing technologies. To investigate the clustering capacity of BLSOM for sequences derived from a wide range of eukaryotes, we first analyzed tetra- and pentanucleotide frequencies in ca. 1,800,000 nonoverlapping 5 kb sequences as well as ca. 900,000 nonoverlapping 10 kb sequences and overlapping 100 kb sequences with a 10 kb sliding step from 101 eukaryotic genomes, most of which were completely sequenced. To analyze sequences derived even from lower eukaryotes with small genome sizes in accurate detail, excess representation of higher eukaryotes' sequences derived from their large genomes had to be avoided. Therefore, for higher eukaryotes, 100 kb sequences were selected randomly from each large genome up to 200 Mb and used for the BLSOM analyses. In DNA databases, only one strand of a pair of complementary sequences is registered, and the choice between the two complementary sequences is often arbitrary in the registration. When global characteristics of oligonucleotide composition in the genome are considered, the distinction of frequencies between the complementary oligonucleotides (e.g., AAAC versus GTTT) is not important in most cases. In the present study, BLSOM was constructed with frequencies for degenerate sets in which the frequencies of a pair of complementary tetra- or pentanucleotides were added (DegeTetra- or DegePenta-BLSOM in Figure 1); this process roughly halved the computation time. The oligonucleotide frequencies were initially analyzed by PCA, and the resulting first and second principal components were used to set the initial weight vectors for the successive BLSOM. After 145 cycles of BLSOM learning, oligonucleotide frequencies in the fragment sequences were represented by the final weight vectors in the two-dimensional array. The resulting BLSOM revealed the clear separation (self-organization) of genomic fragments according to phylotypes (Figure 1). The computational time of DegeTetra-BLSOM was approximately six hours using high performance parallel computers; if PC servers were used, the time required for computation was 100 or more times as long.

In this figure, a node that included sequences from a single phylogenetic family was indicated in the color representing the family, while a node that included sequences from more than one family was indicated in black. Sequences from each family were clustered tightly on these tetra- and pentanucleotide BLSOMs. The accuracy level of separation by family on the 5-, 10-, and 100 kb DegeTetra-BLSOM was approximately 74, 90, and 99%, respectively, showing that longer sequences gave the higher accuracy of clustering according to phylotype.

In the 100 kb BLSOMs, the phylogenetic family territories were surrounded by contiguous white nodes, which contained no genome sequences in the final map. In other words, family borders could be drawn automatically on the basis of the contiguous white nodes. This is because the representative vectors of the family-specific nodes were very distinctive between different families even near territory borders. Much narrower white borders were observed within a certain family territory and primarily represented genus/species separation.

### 3.2. Diagnostic Oligonucleotides for Phylotype-Specific Clustering

%GC has long been used as a fundamental parameter for the phylogenetic characterization of species. We previously found that the %GC from the weight vector representing each node on a BLSOM was reflected in the horizontal axis [13]. Supporting the previous finding, the %GC increased globally from left to right on the present BLSOMs (the DegePenta-%GC panel in Figure 1(c)); therefore, sequences with high %GC (red in Figure 1(c)) were located on the right side. Importantly, sequences even with the same %GC were clearly separated on BLSOMs by a complex combination of oligonucleotide frequencies, resulting in accurate phylotype separation.

**Figure 2:**
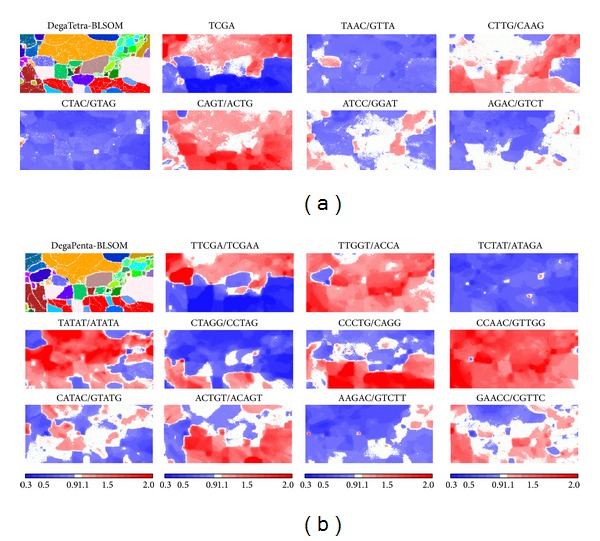
The level of each pair of complimentary tetra- or pentanucleotides on 100 kb BLSOMs. (a) DegeTetra- and (b) DegePenta-BLSOMs were those listed in Figure 1. Diagnostic examples of phylotype separations are presented. The level of each pair of complimentary tetra- or pentanucleotides in each node on the 100 kb DegeTetra- and DegePenta-BLSOMs in Figure 1 was calculated and normalized with the level expected from the mononucleotide composition of the node. The observed/expected ratio is indicated in colors shown at the bottom of the figure.

BLSOM provides a powerful ability for visualizing diagnostic oligonucleotides that contribute to the self-organization of sequences according to phylotype [14, 15]. The frequency of each tetra- or pentanucleotide obtained from the weight vector for each node in the 100 kb DegeTetra- or DegePenta-BLSOM listed in Figure 1 was calculated and normalized with the level expected from the mononucleotide composition calculated from the vectorial data representing each node. The observed/expected ratios were illustrated in red (overrepresented), blue (underrepresented), and white (moderately represented) in Figure 2. This normalization allowed oligonucleotide frequencies in each node to be studied independent of the %GC of the sequences [15]. Transitions between red (overrepresentation) and blue (underrepresentation) for various tetra- and pentanucleotides often coincided exactly with phylotype territory borders, indicating that BLSOM recognized the phylotype-specific combination of oligonucleotide frequencies that was the representative signature of each genome. Seven tetranucleotide and eleven pentanucleotide examples, which were diagnostic for phylotype territory formation, were presented in Figure 2; distribution patterns for all tetra- and pentanucleotides were listed in Supplementary Figures S1 and S2 (see Figures S1 and S2 in Supplementary Material available online at http://dx.doi.org/10.1155/2014/985706). It should be stressed that complex combinations of many oligonucleotides contributed to the self-organization of sequence fragments according to phylotype and that BLSOM could visualize the diagnostic oligonucleotides in an easy-to-understand manner.

### 3.3. Visualization of 12* Drosophila* Genome Sequences

**Figure 3:**
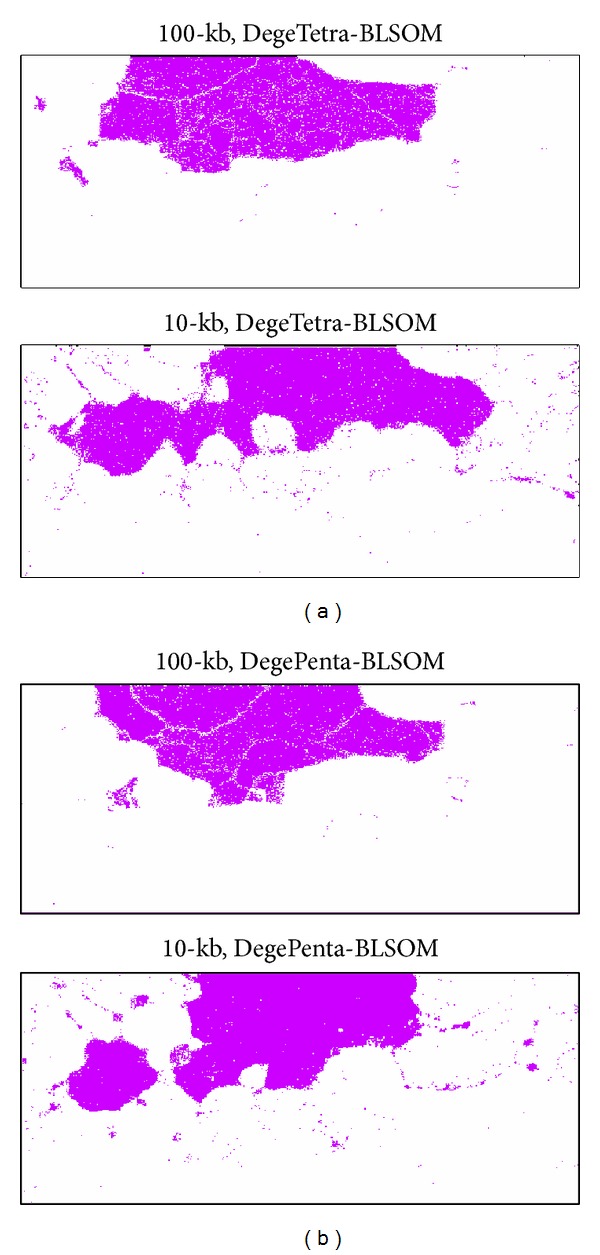
Distribution patterns of nodes containing sequences derived only from 12* Drosophila* genomes on 100 kb DegeTetra- (a) and DegePenta- (b) BLSOMs, which were listed in Figure 1.

**Figure 4:**
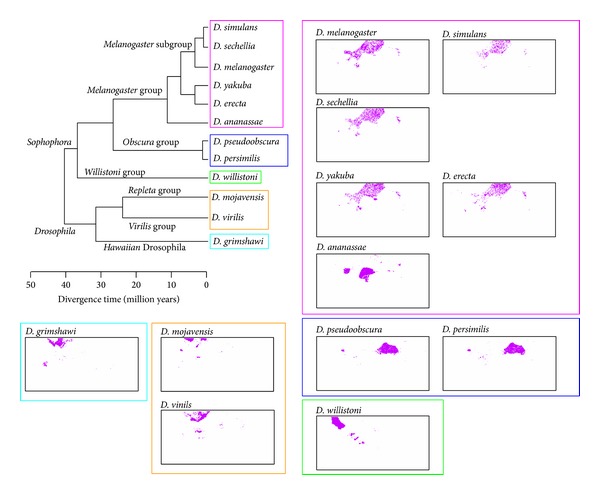
Distribution pattern for each of the 12* Drosophila* genomes on 100 kb DegePenta-BLSOM listed in Figure 1. The phylogenetic tree for the 12* Drosophila *species was obtained from Flybase [17]. The species belonging to one of the five* Drosophila *groups were separately specified by five boxes with different colors in the phylogenetic tree. A distribution pattern of nodes containing sequences derived from one* Drosophila* species was presented in the box marked with the color representing the respective* Drosophila *group, which was specified in the phylogenetic tree.

In Figure 1, we analyzed 101 eukaryotic genomes that covered a wide range of phylogenetically distant eukaryotes. Then, to investigate the clustering capacity of BLSOM for the genomes of phylogenetically closely related species, nodes containing sequences derived only from 12* Drosophila* genomes were specifically marked in pink on the BLSOMs (Figure 3). In Figure 4, we examined the correlation between the classification pattern on the DegePenta-BLSOM and the phylogenetic classification according to* Drosophila* genomes by referring to a phylogenetic tree for the 12* Drosophila *species, which was obtained from Flybase [17]. Distribution patterns for species belonging to one of the five* Drosophila *groups, which were specified by five boxes in the phylogenetic tree, were similar to each other, but patterns for species belonging to different groups were clearly distinct; the same result was obtained in DegeTetra-BLSOM (data not shown). This showed that BLSOM could properly extract sequence characteristics of* Drosophila *genomes with phylogenetic clustering through sequence homology searching. The diagnostic oligonucleotides contributing to the clustering according to the* Drosophila* group on BLSOMs could be assigned as shown in Figure 2. For example, the frequency of CTTCG was low only for the melanogaster group, while those of ATTCX, TGGTC, and TTCGY were low only for the virilis group; see the distribution patterns for tetra- and pentanucleotides listed in Supplementary Figures S1 and S2.

### 3.4. BLSOMs Constructed with 12* Drosophila* Genomes

**Figure 5:**
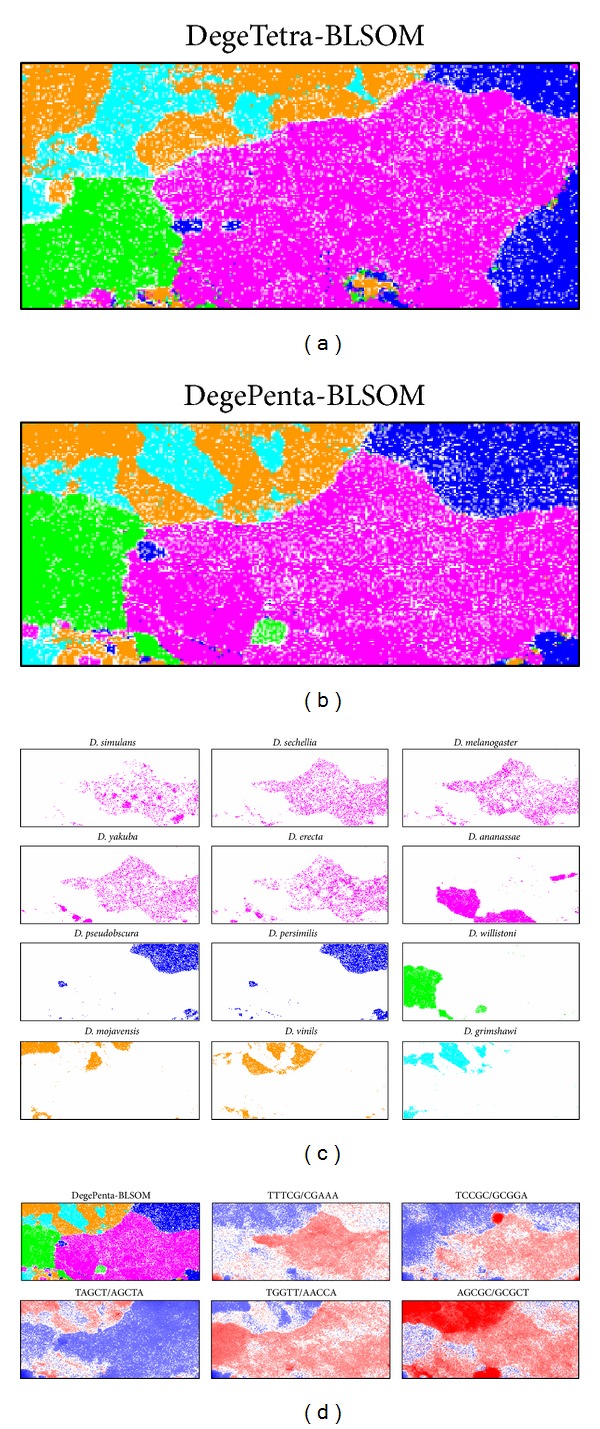
DegeTetra- (a) and DegePenta- (b) BLSOMs for the overlapping 100 kb sequences with a 10 kb sliding step derived from the 12* Drosophila* genomes. Nodes containing sequences derived from genomes belonging to more than one group were indicated in black and those belonging to one group were indicated in colors, which were used to distinguish the boxes representing the five* Drosophila* groups. (c) The distribution pattern for each* Drosophila* genome on the DegePenta-BLSOM, which was listed in (b). Nodes were indicated in the color representing the group. (d) The level of each pair of complimentary pentanucleotides on the DegePenta-BLSOM listed in (b) was shown as described in Figure 2.

The results presented in Figures 1 and 2 showed that BLSOM could analyze almost all eukaryotic genome sequences available from the current DNA databanks simultaneously on a single map and visualize their genome signatures. This comprehensive, panoramic view of a huge number of genome sequences will become increasingly important because a large number of genomes (closely or distantly related with each other) have been sequenced intensively with next-generation DNA sequencers. This is one capacity of BLSOM. The results presented in Figures 3 and 4 also showed that BLSOM had a good capacity for distinguishing closely related species. When focusing only on closely related species, such as those belonging to one genus, BLSOM constructed only for these species may provide much detailed information. To examine this possibility, we constructed DegeTetra- and DegePenta-BLSOMs for 100 kb sequences with a 10 kb sliding step derived from the 12* Drosophila* genomes. Nodes containing sequences only from one* Drosophila* group listed in Figure 4 were marked in the color representing the group (Figures 5(a) and 5(b)). In Figure 5(c), nodes containing sequences only from one species on the DegePenta-BLSOM were marked in the color representing the species. Species belonging to one group had similar patterns or their territories were adjacently located; for their phylogenetic closeness, refer to the phylogenetic tree listed in Figure 4. It should also be noted that there was segmentation of the territory of one species and/or that there were minor satellite territories apart from its major territory. Similar results were also obtained from DegeTetra-BLSOM (data not shown). These characteristics of individual species on BLSOMs may presumably represent genome characteristics of these species.

In Figure 5(d), examples of the pentanucleotides diagnostic for separation by group/species were presented; the results of all pentanucleotides were presented in Supplementary Figure S3. Although the functions of the oligonucleotides diagnostic for separation by group/species have not yet been studied, some of them may relate to the biological functions and/or evolutionary processes leading to the construction of the present genomes [18–20].

## 4. Discussion and Conclusion

When characteristic oligonucleotides, both underrepresented and overrepresented in each genome, are considered (Figures 2 and 4), various molecular mechanisms, including context-dependent mutation, repair, and modification, may be responsible [1–8]. For overrepresented sequences, preferences for sequences recognized by ubiquitous DNA-binding proteins and ubiquitous repetitive elements must be considered. It should also be mentioned that oligonucleotides, such as tetra-heptanucleotides, often represent motif sequences responsible for sequence-specific protein binding (e.g., transcription factor binding). Occurrences of such motif oligonucleotides should differ from the occurrences expected from the mononucleotide composition in the respective genome and may differ among genomes and among genomic portions within a single genome. Actually, we have recently found that a pentanucleotide-BLSOM for the human genome can detect the characteristic enrichment of many transcription-factor-binding motifs in pericentric heterochromatin regions [21]. Functional signals, such as transcription-regulatory signals, are typically longer than pentanucleotides; therefore, analyses of longer oligonucleotides will become important. To conduct BLSOM with longer oligonucleotides, such as hexa- and heptanucleotides (4,096- and 16,384-dimensional data), for a large number of currently available genome sequences, large-scale computation using a high-performance supercomputer is essential, and the BLSOM algorithm is suitable for high-performance parallel computing.

For almost half of genes from the novel genomes sequenced, it has become clear that protein functions cannot always be estimated through sequence homology searching. We have applied BLSOM to protein sequence studies to analyze the frequency of oligopeptides and found the separation (self-organization) of proteins according to their functions [22]. This finding indicates that the BLSOM can be used for protein function estimation that does not rely on sequence homology searching and troublesome and confusing sequence alignment. Large-scale BLSOM analyses of a large amount and a wide variety of genome and protein sequences facilitate efficient extraction of fundamental information that supports research and development in a broad range of life sciences and industrial fields.

## Supplementary Material

Supplemental Figures list the distribution patterns of each pair of complimentary tetra- and pentanucleotide in 100-kb DegeTetra- and DegePenta-BLSOM for the overlapping 100-kb sequences of 101 eukaryotic and 12 Drosophila genomes.
Supplementary Figure S1. Level of each pair of complimentary tetranucleotide in 100-kb DegeTetra-BLSOM for the overlapping 100-kb sequences of 101 eukaryotic genomes.
Supplementary Figure S2. Level of each pair of complimentary pentanucleotide in 100-kb DegePenta-BLSOM for the overlapping 100-kb sequences of 101 eukaryotic genomes.
Supplementary Figure S3. Level of each pair of complimentary pentanucleotide in 100-kb DegePenta-BLSOM for the overlapping 100-kb sequences of 12 Drosophila genomes.
