# Supplementary material for: Visualization of Genome Signatures of Eukaryote Genomes by Batch-Learning Self-Organizing Map with a Special Emphasis on Drosophila Genomes
Source: Biomed Res Int. 2014 Mar 11;2014:985706. doi: 10.1155/2014/985706 (PMC3967822; doi:10.1155/2014/985706)
Supplement: Supplementary file 1 — Supplemental Figures list the distribution patterns of each pair of complimentary tetra- and pentanucleotide in 100-kb DegeTetra- and DegePenta-BLSOM for the overlapping 100-kb sequences of 101 eukaryotic and 12 Drosophila genomes. Supplementary Figure S1. Level of each pair of complimentary tetranucleotide in 100-kb DegeTetra-BLSOM for the overlapping 100-kb sequences of 101 eukaryotic genomes. Supplementary Figure S2. Level of each pair of complimentary pentanucleotide in 100-kb DegePenta-BLSOM for the overlapping 100-kb sequences of 101 eukaryotic genomes. Supplementary Figure S3. Level of each pair of complimentary pentanucleotide in 100-kb DegePenta-BLSOM for the overlapping 100-kb sequences of 12 Drosophila genomes. [file 985706.f1.pdf]

Supplementary Figure S1.
Level of each pair of complimentary tetranucleotide in 100-kb DegeTetra-BLSOM for the overlapping 100-kb sequences of 101 eukaryotic genomes.

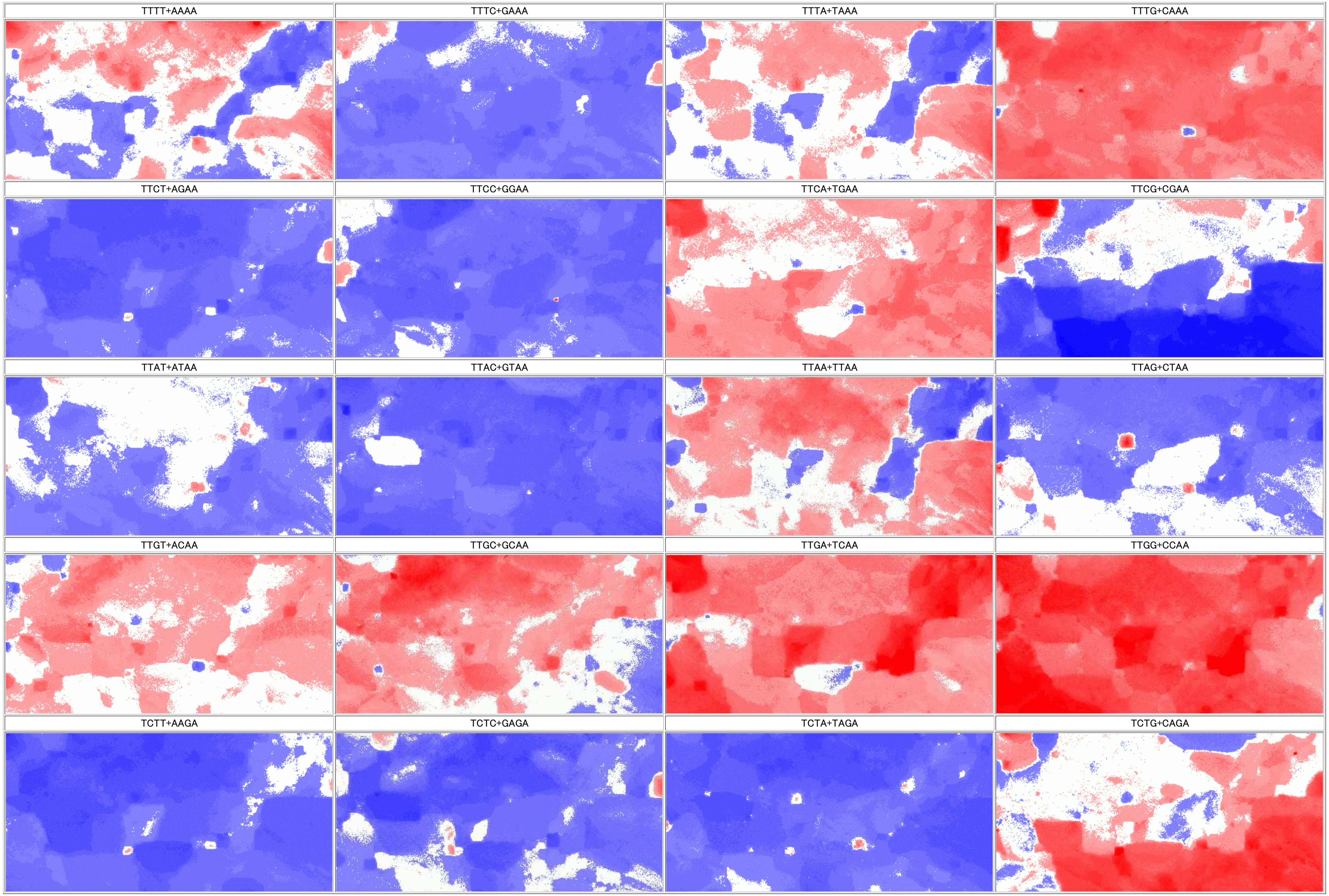

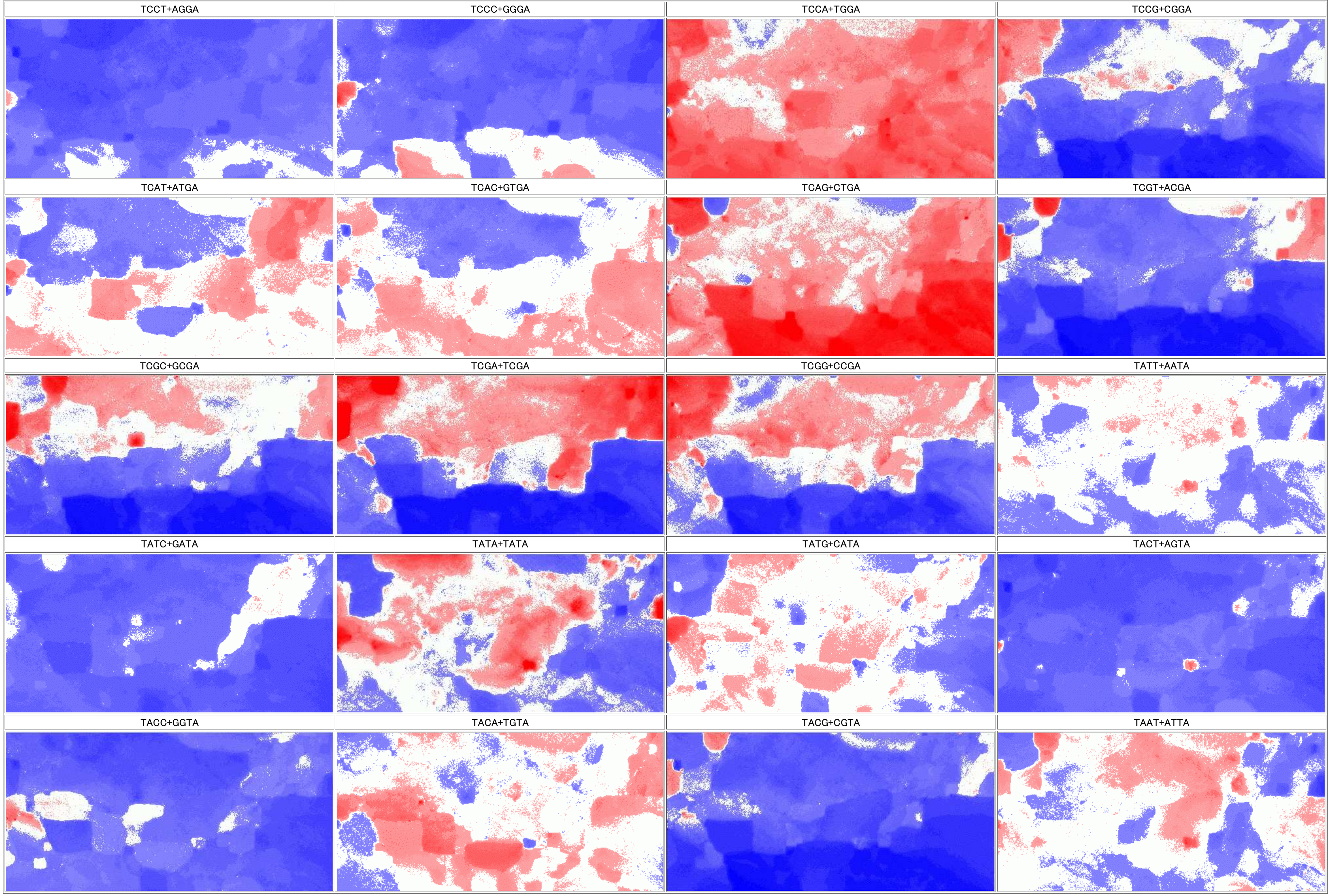

Supplementary Figure S1

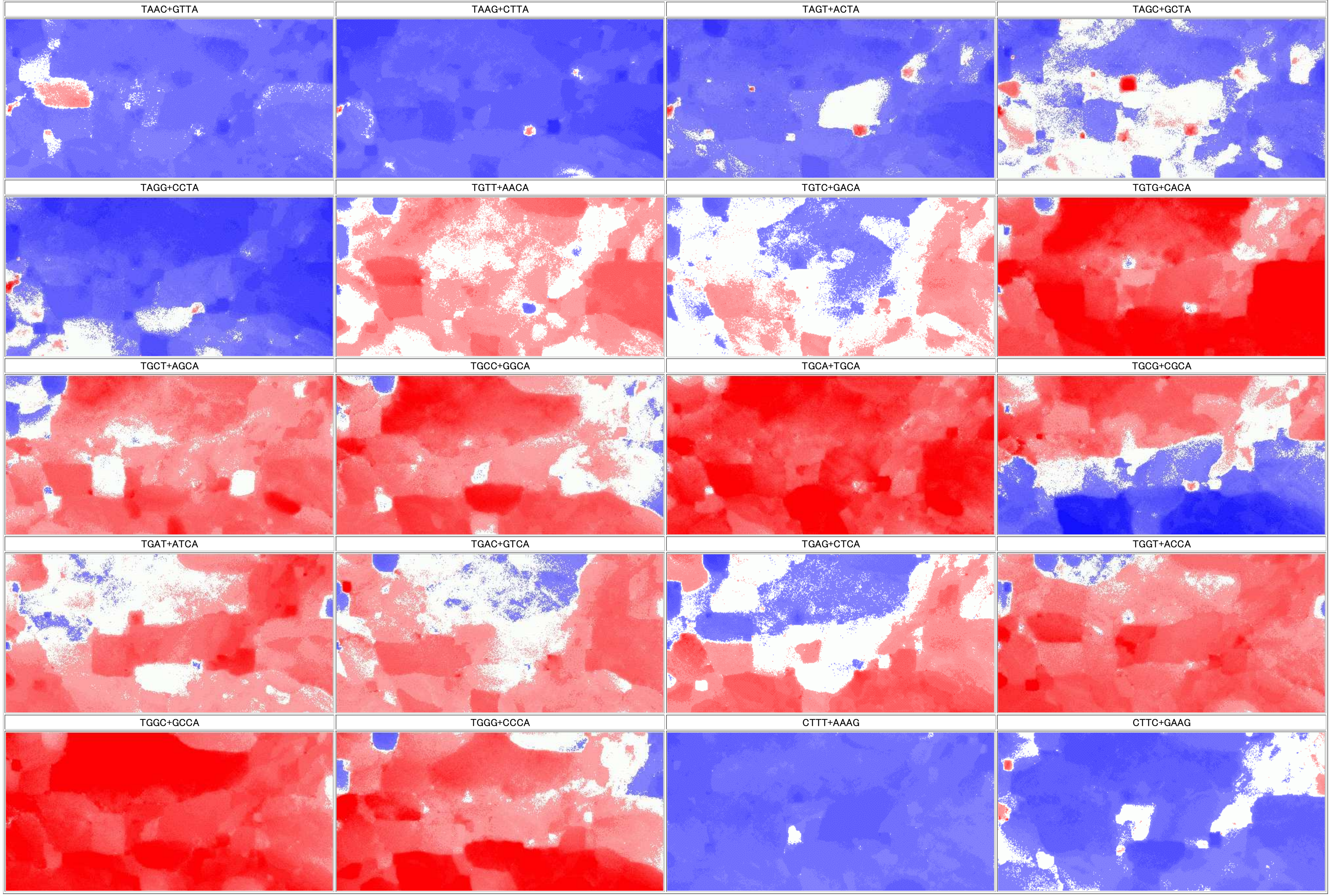

Supplementary Figure S1

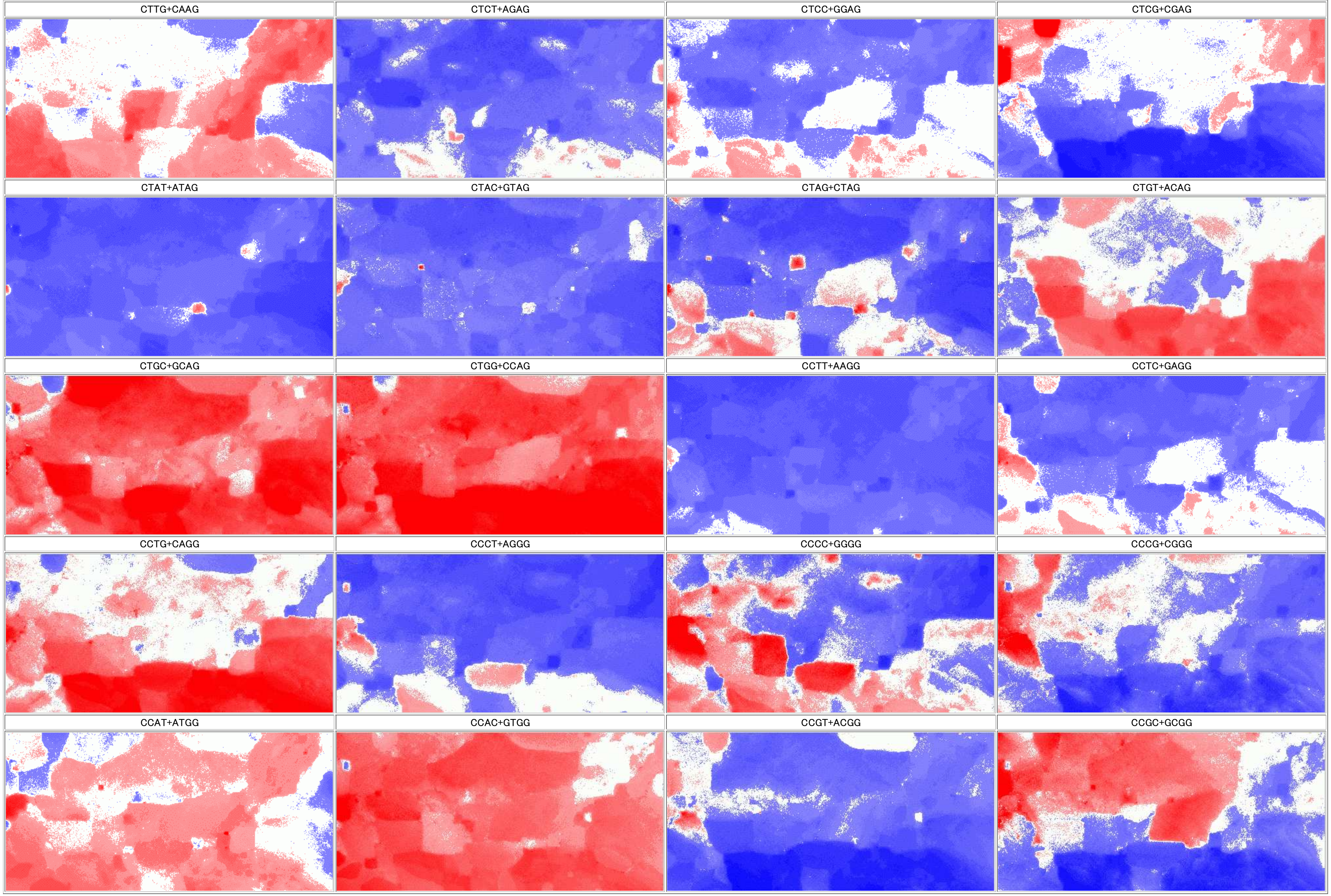

Supplementary Figure S1

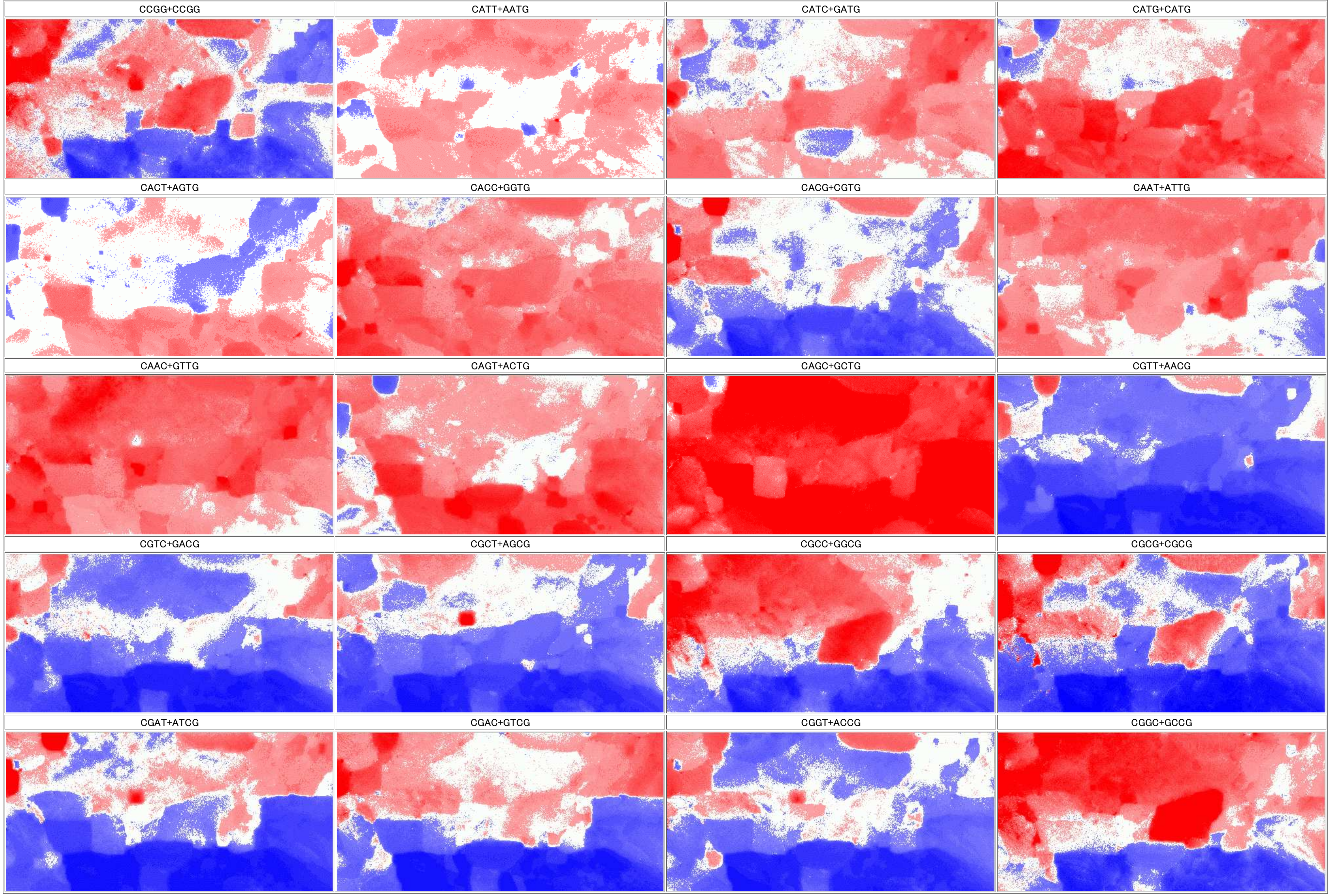

Supplementary Figure S1

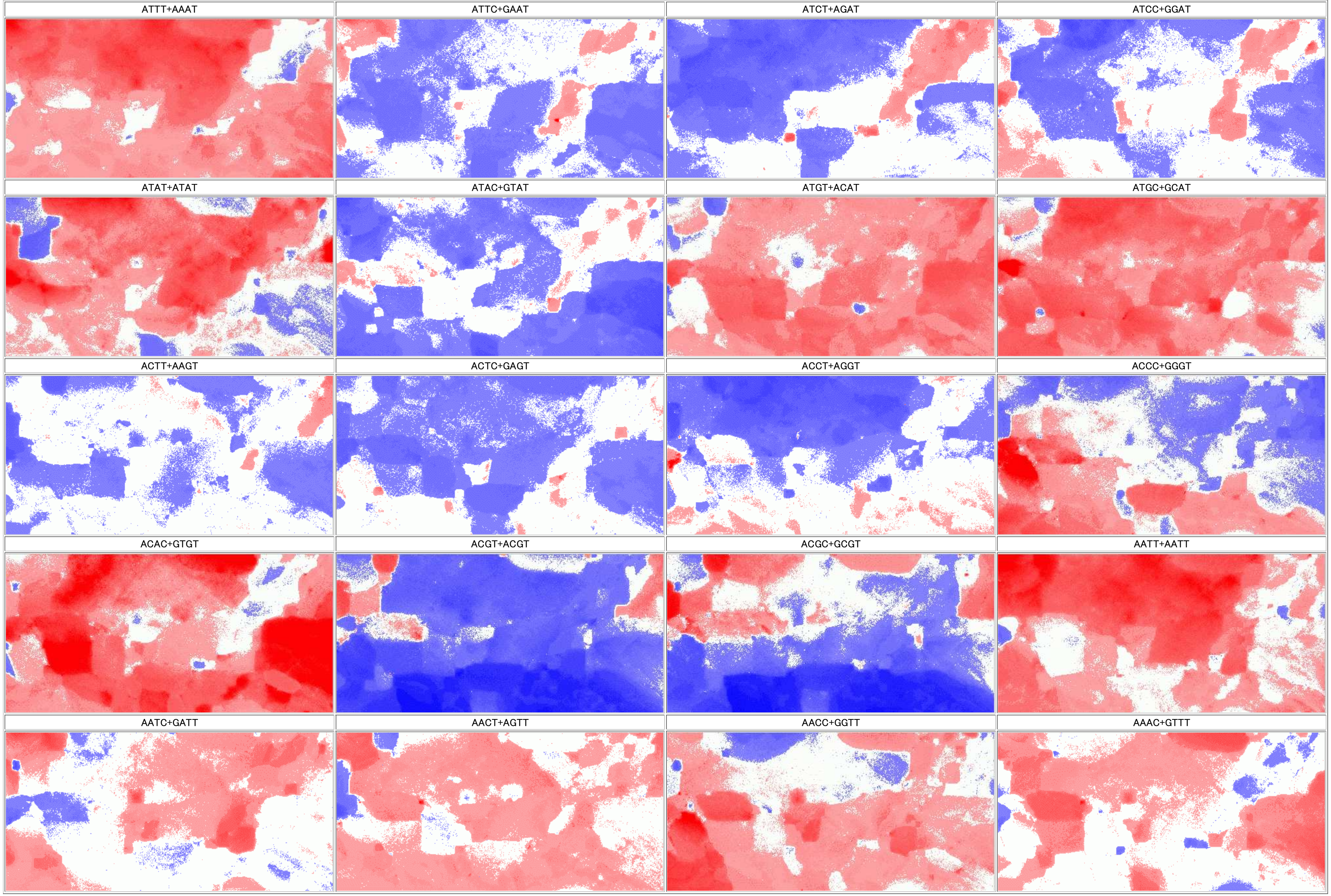

Supplementary Figure S1

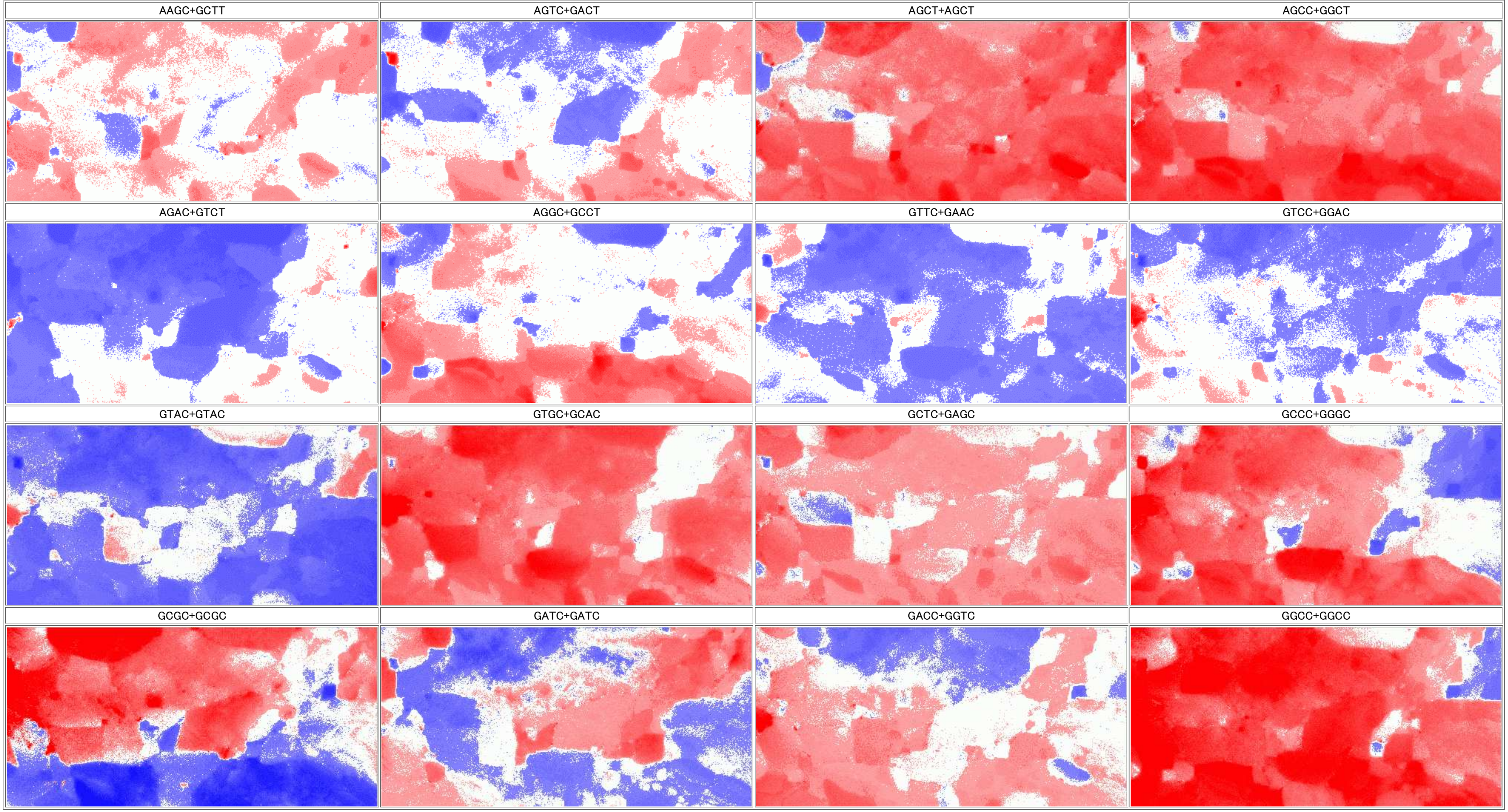

Supplementary Figure S1

Supplementary Figure S2.
Level of each pair of complimentary pentanucleotide in 100-kb DegePenta-BLSOM for the overlapping 100-kb sequences of 101 eukaryotic genomes.

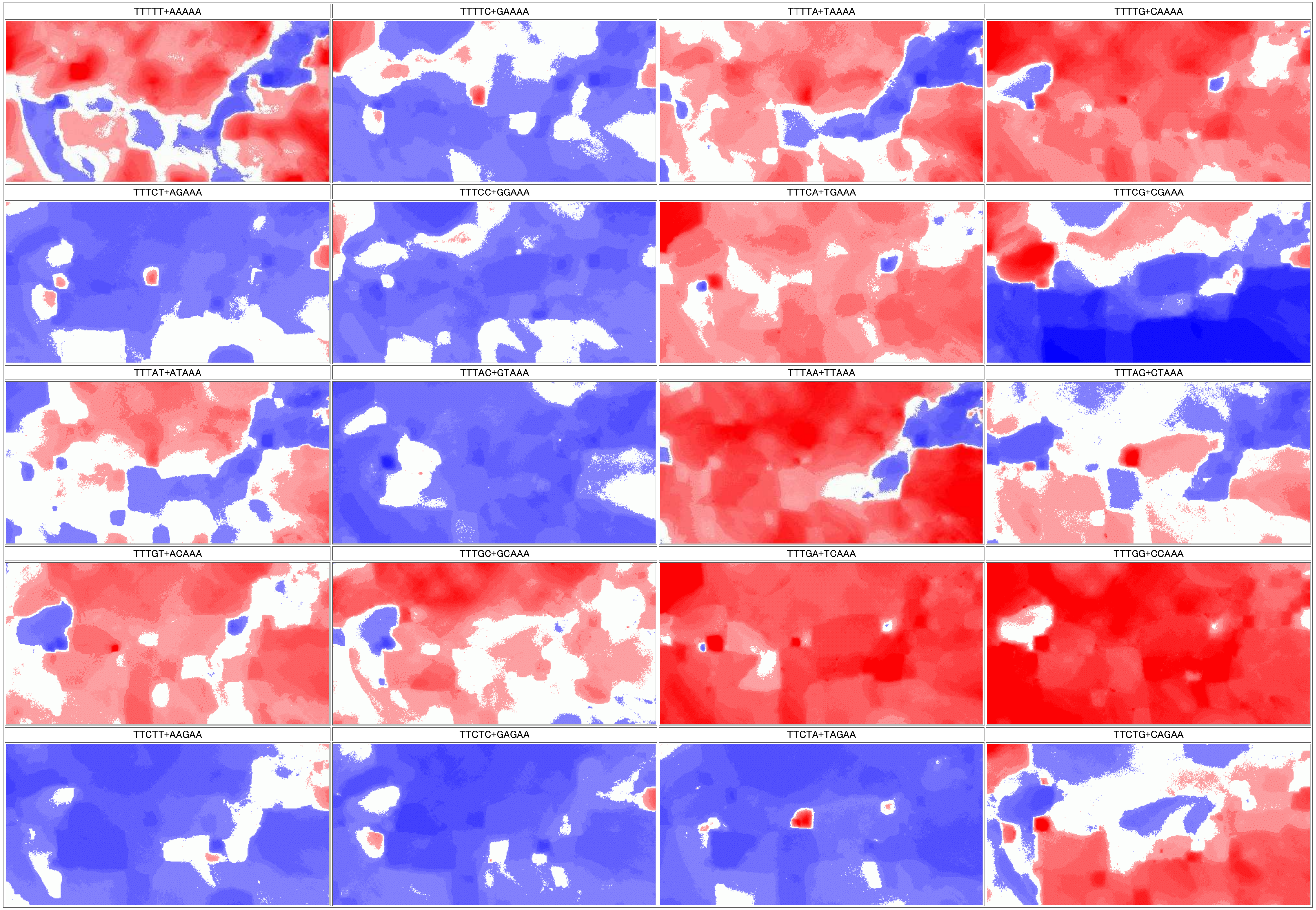

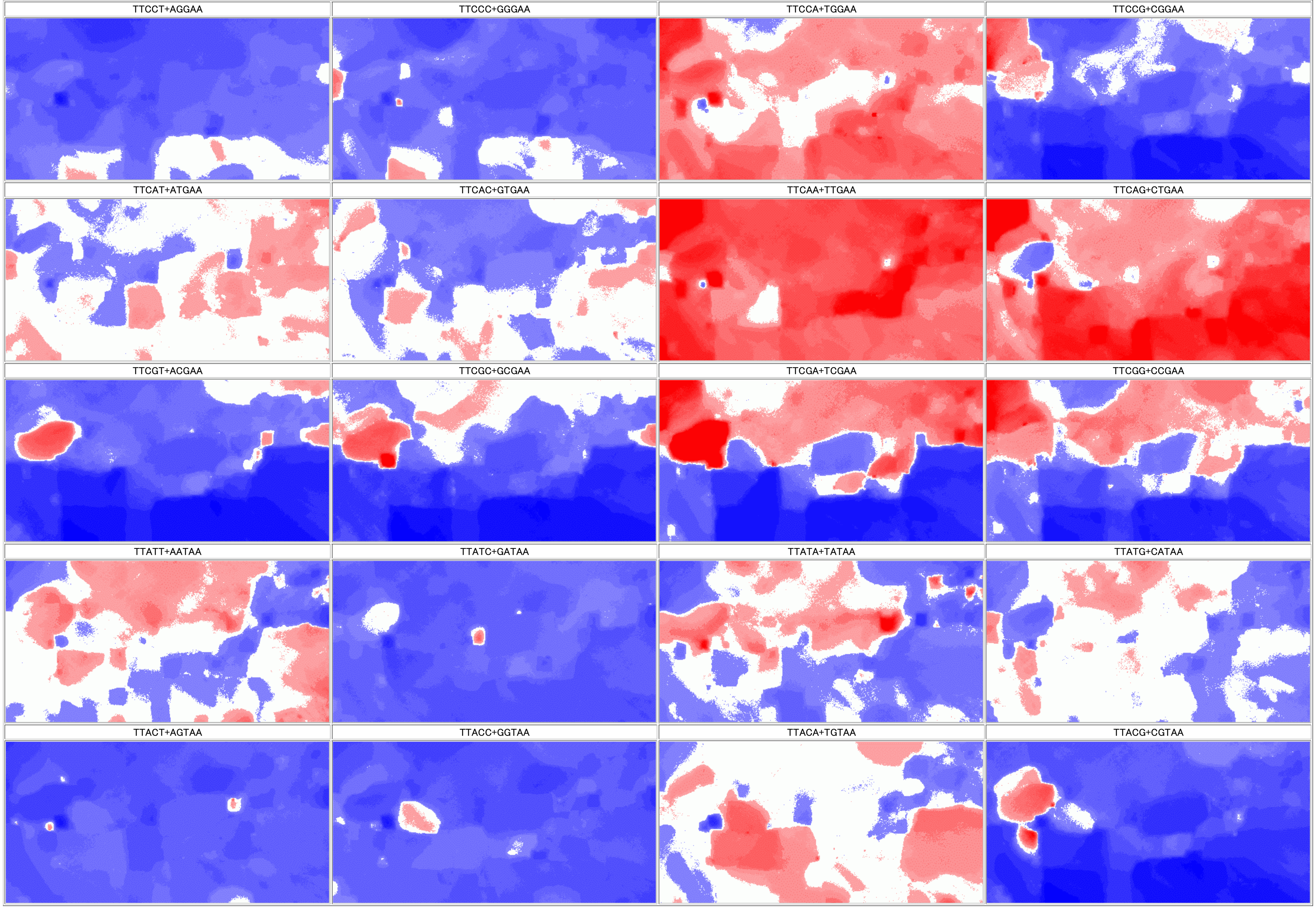

Supplementary Figure S2

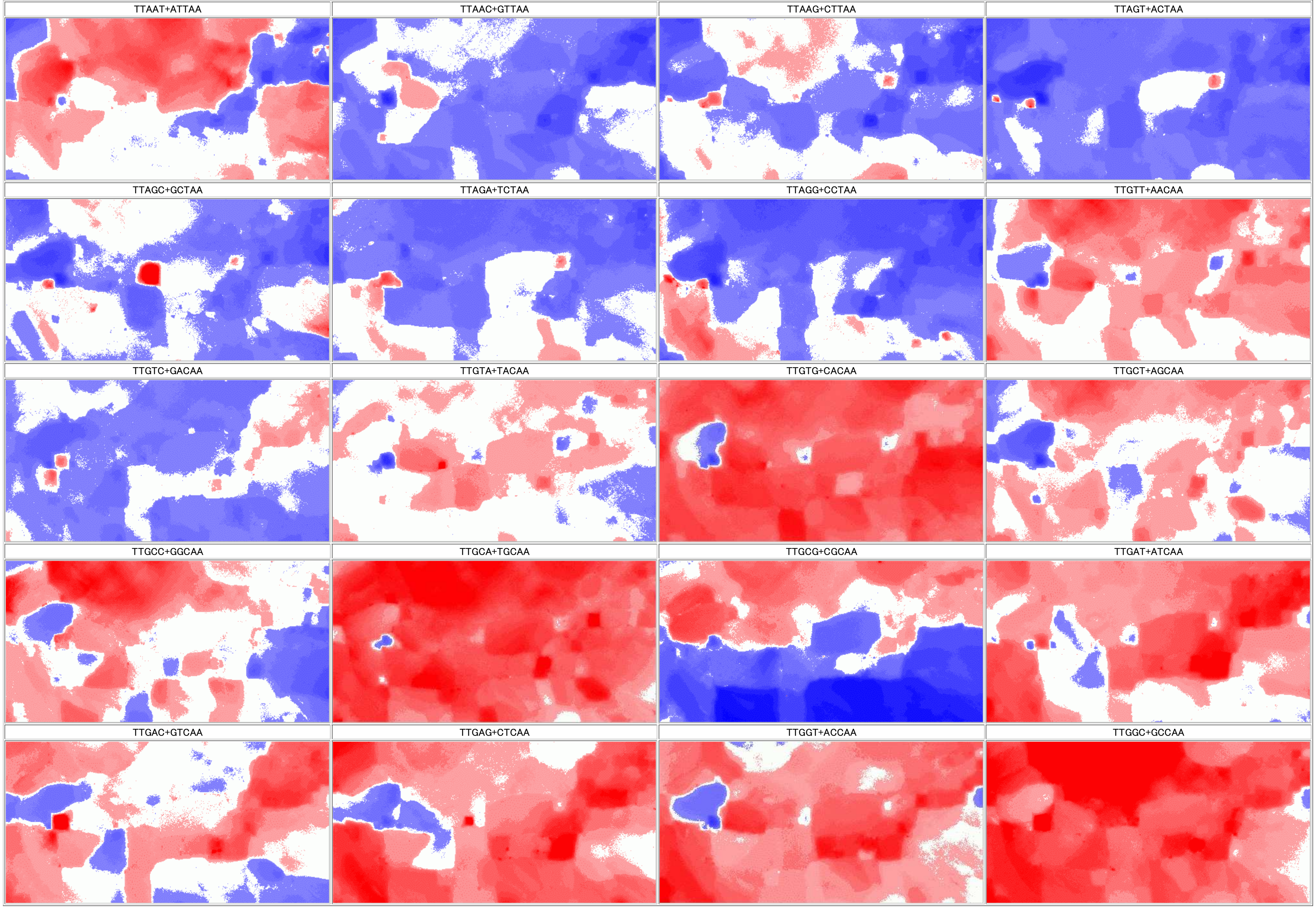

Supplementary Figure S2

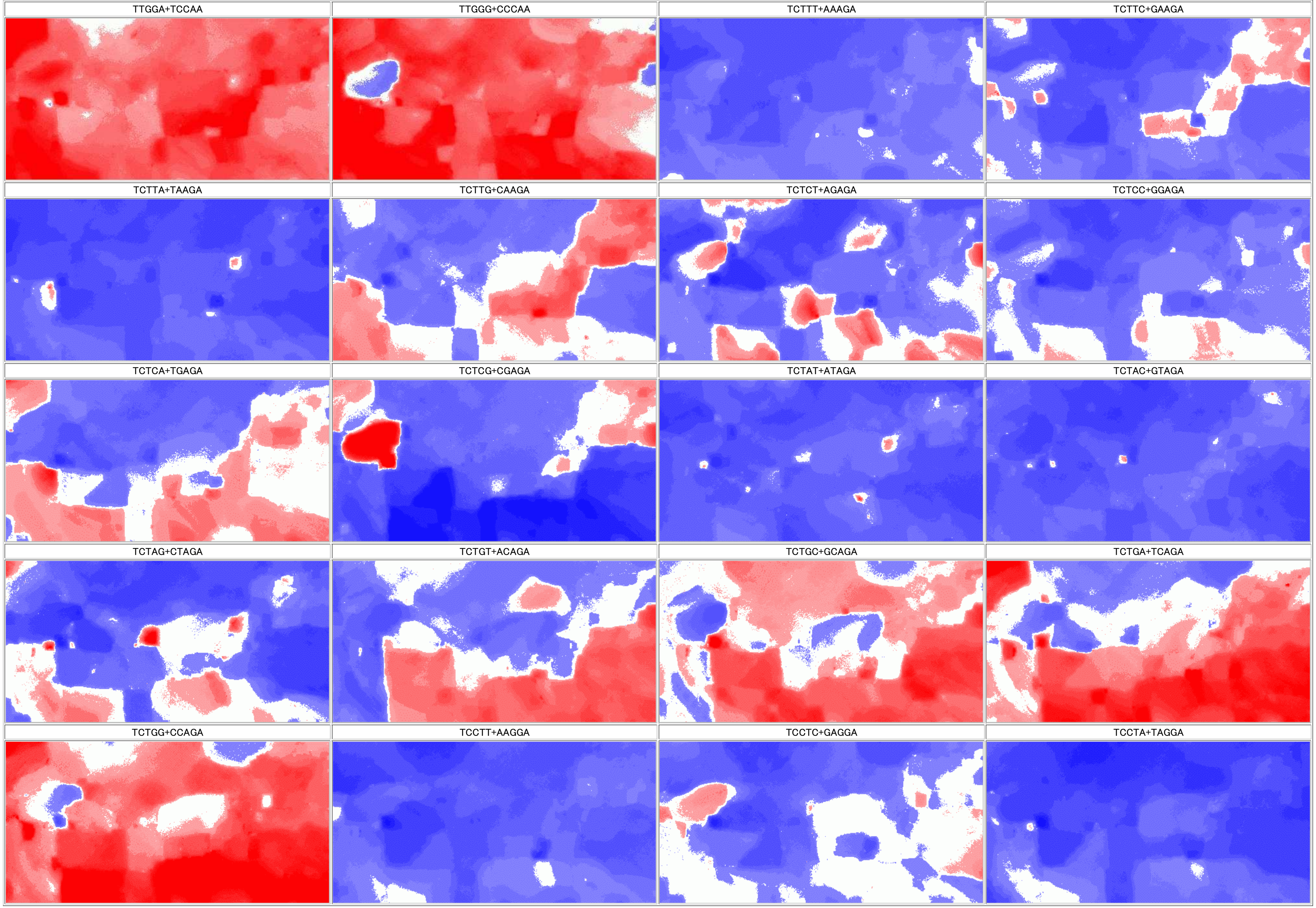

Supplementary Figure S2

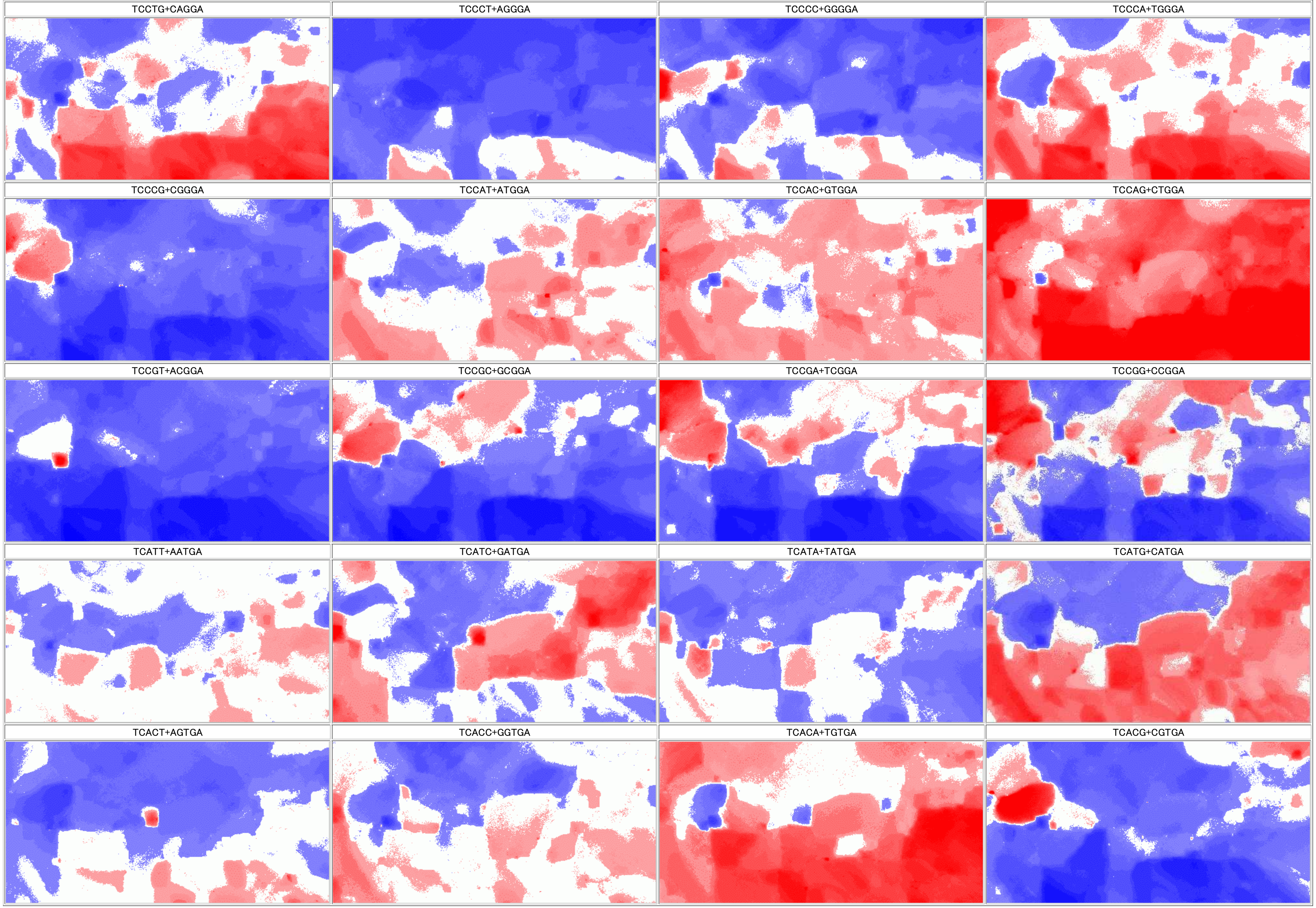

Supplementary Figure S2

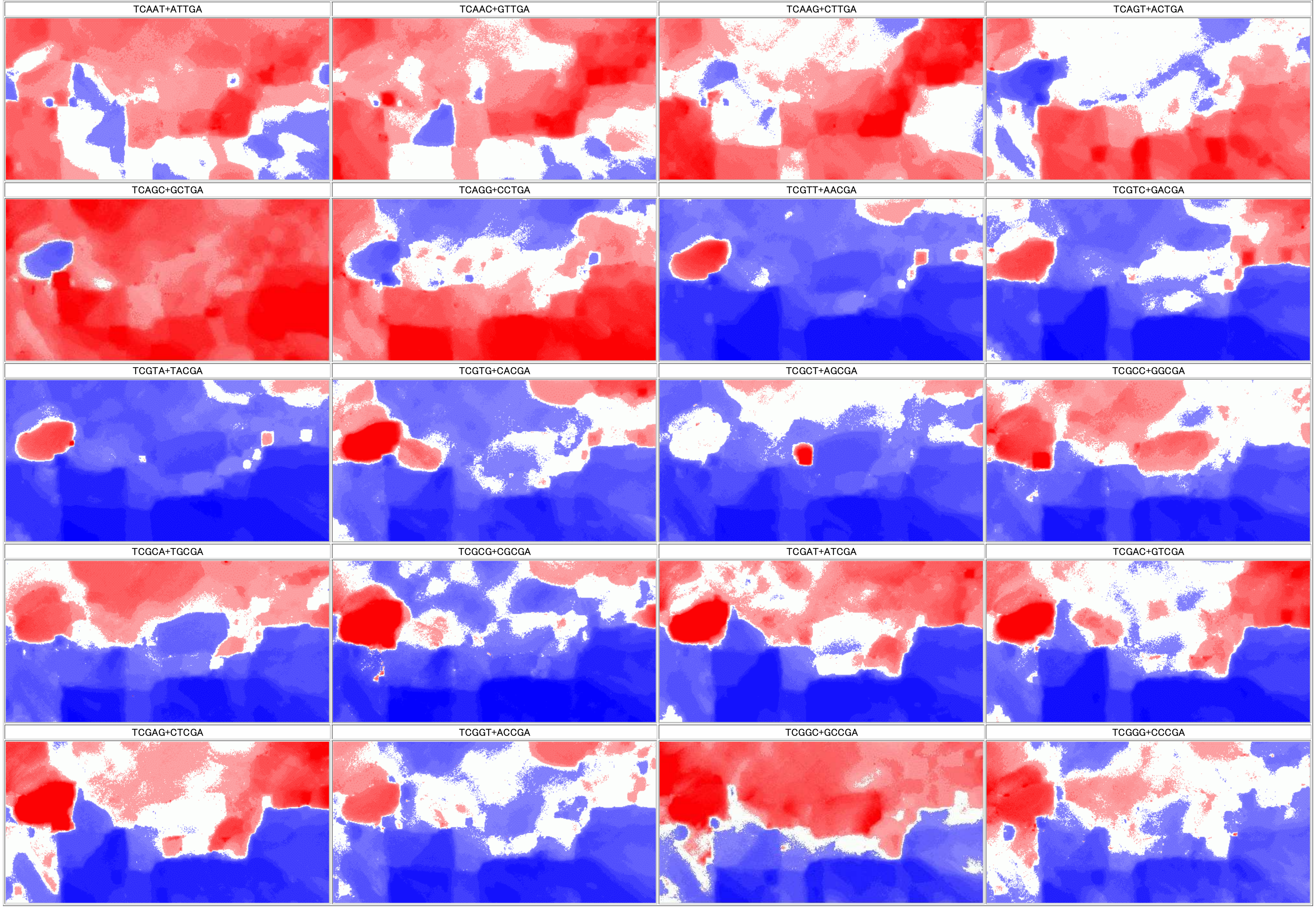

Supplementary Figure S2

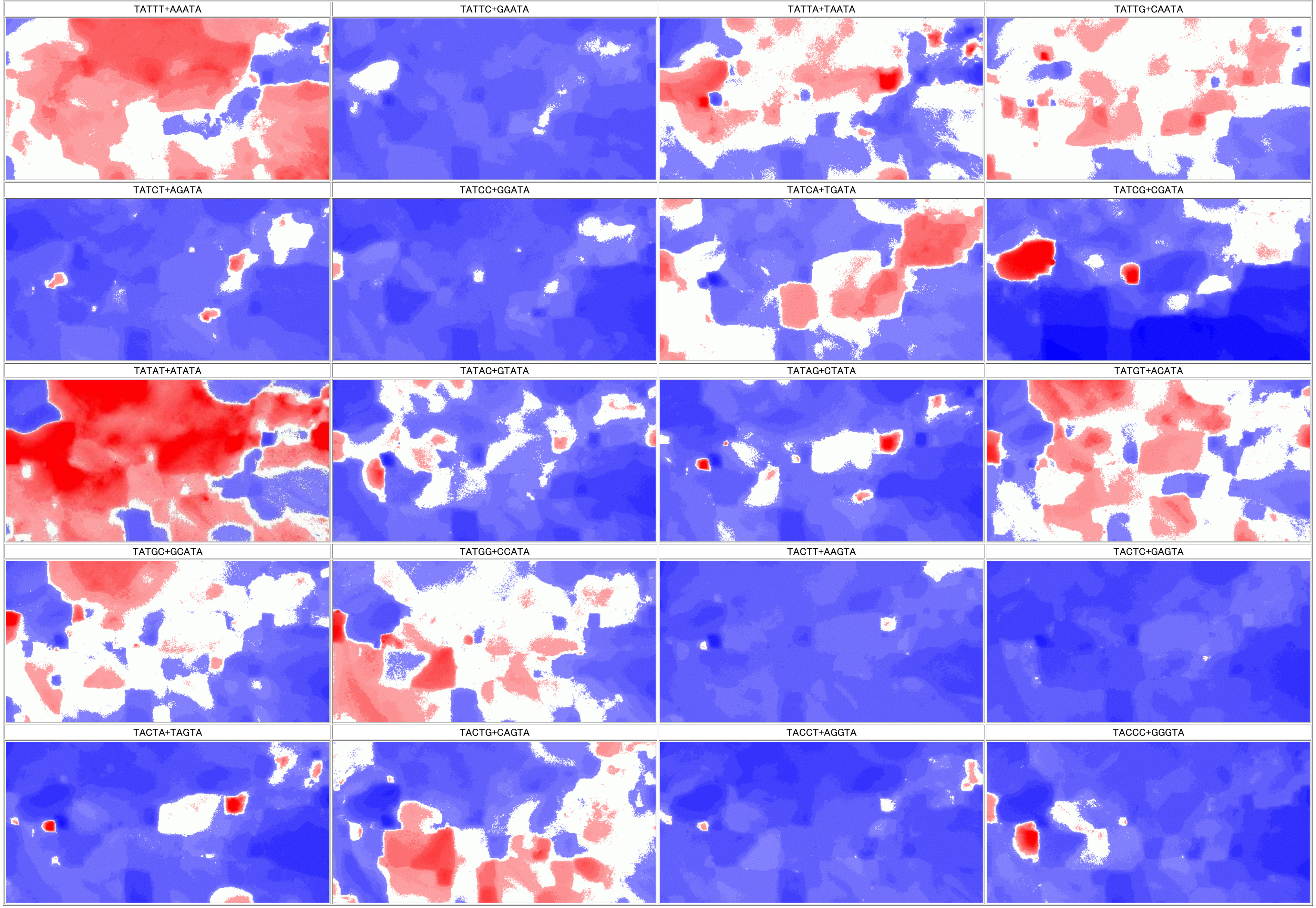

Supplementary Figure S2

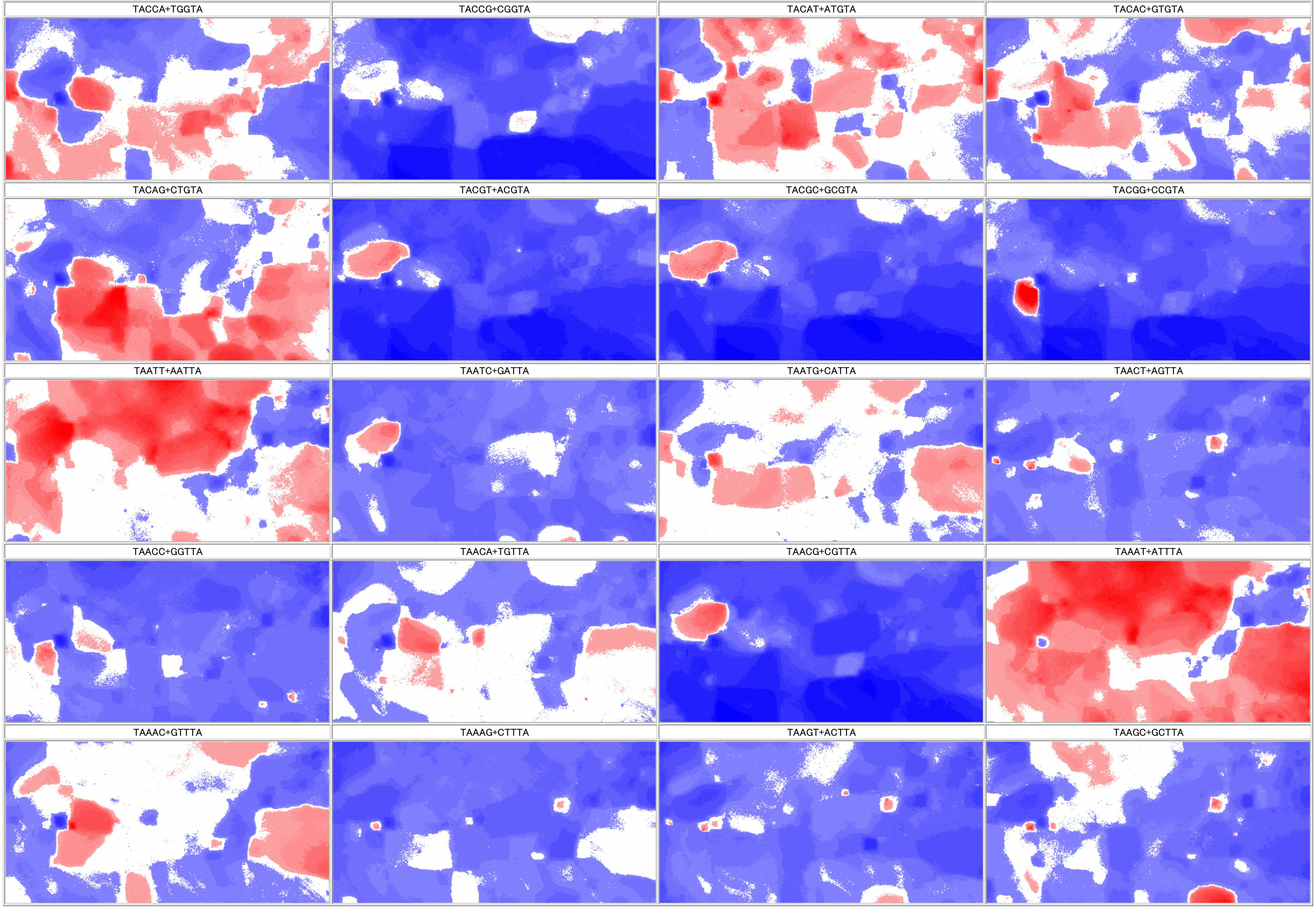

Supplementary Figure S2

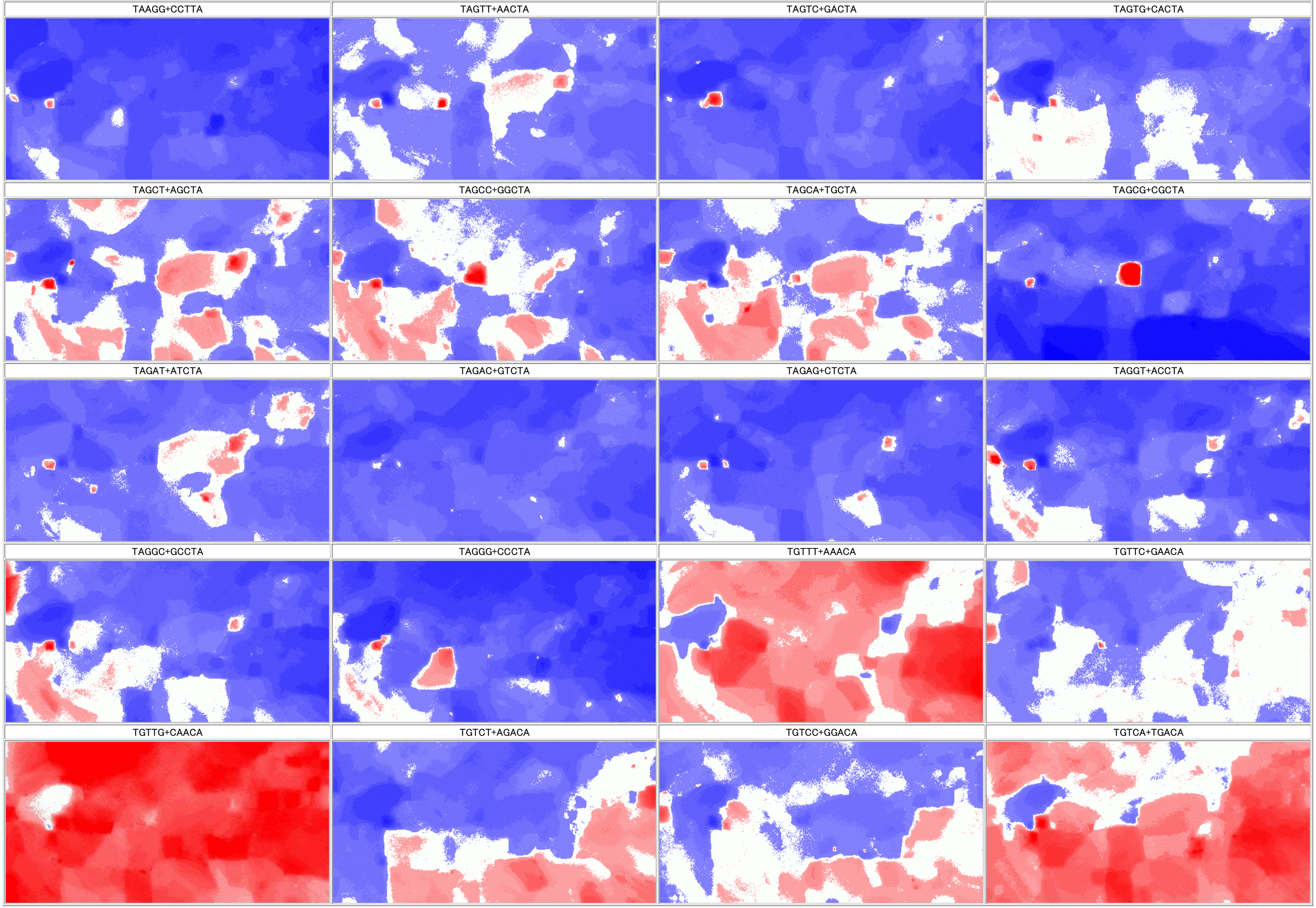

Supplementary Figure S2

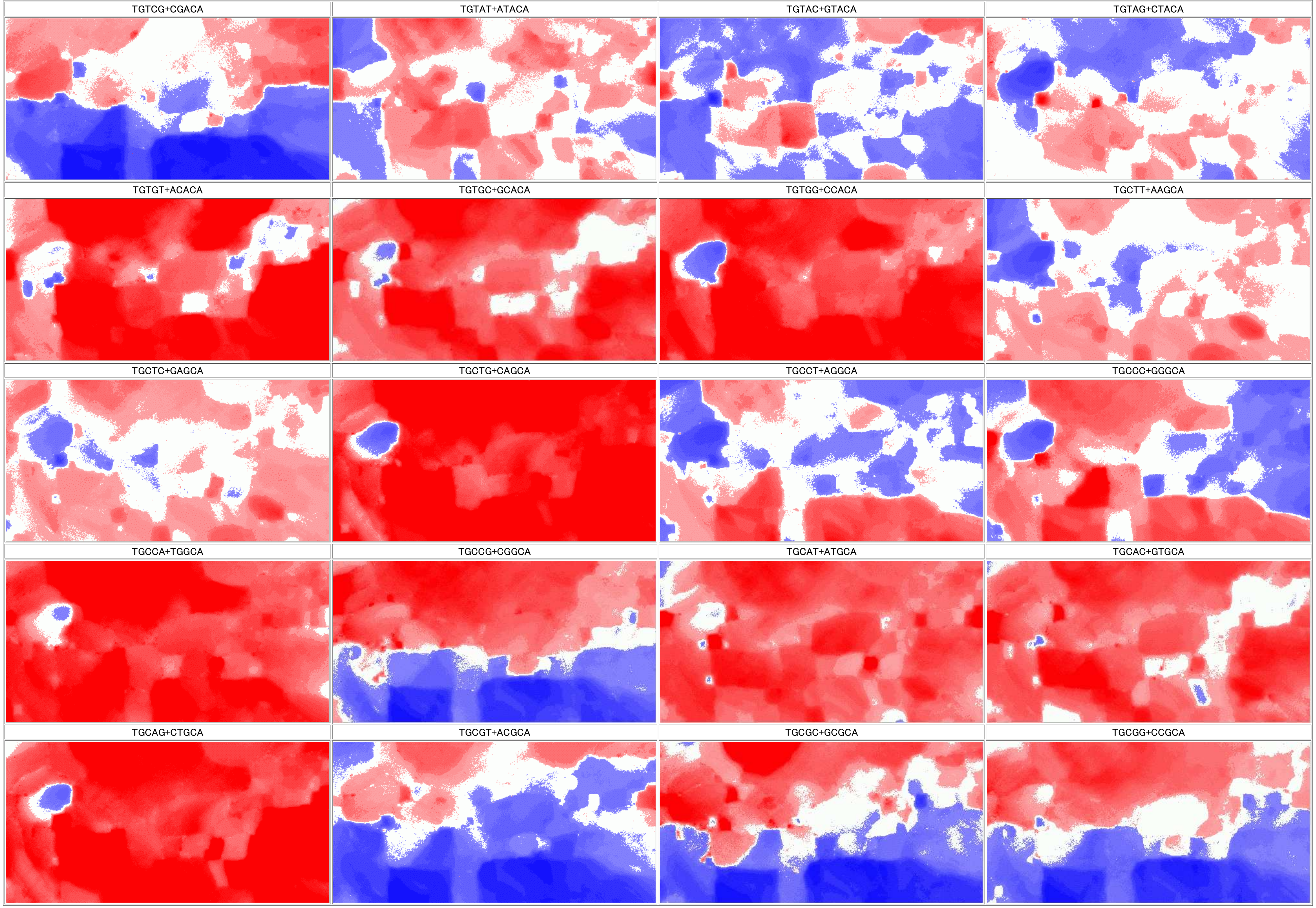

Supplementary Figure S2

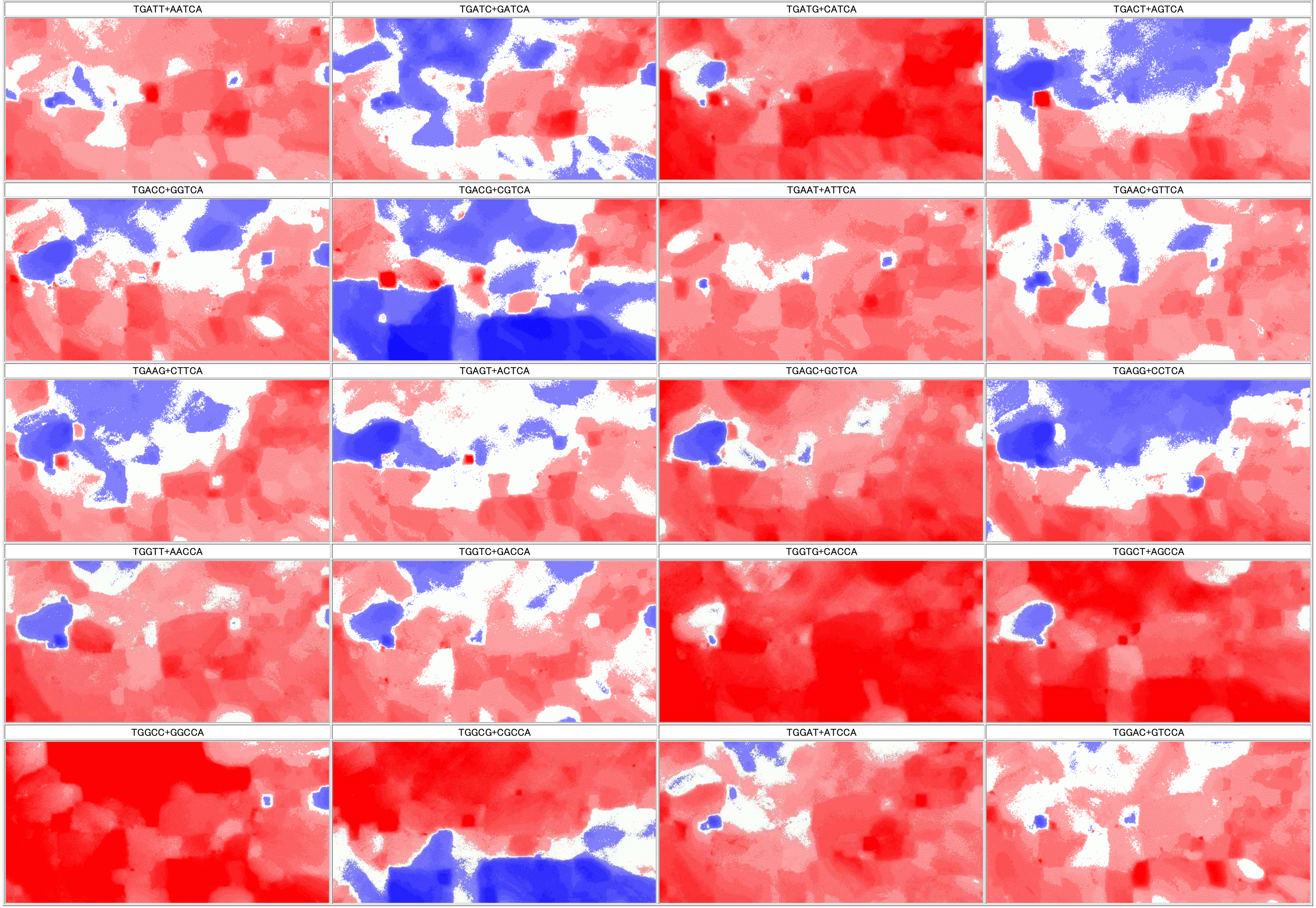

Supplementary Figure S2

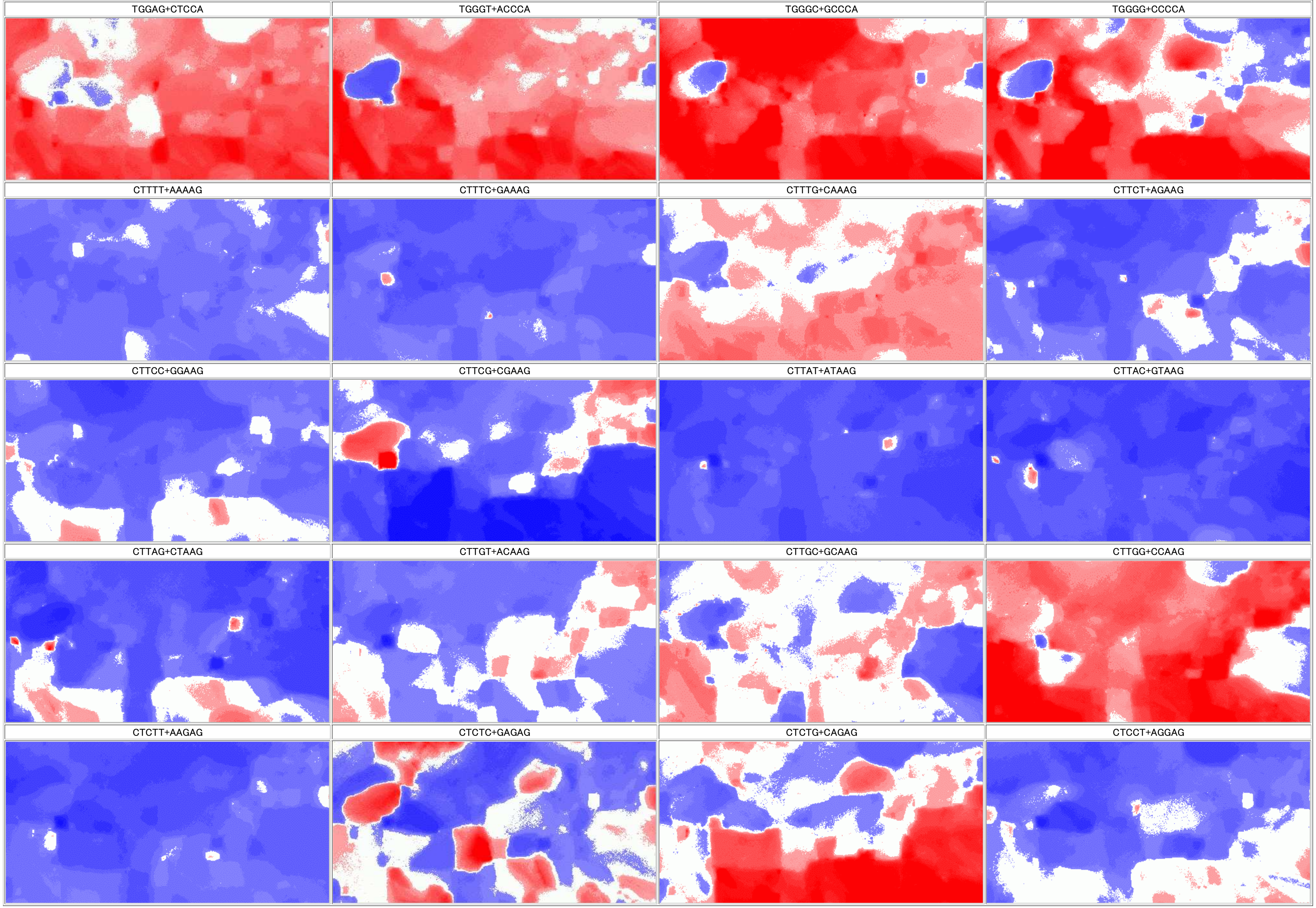

Supplementary Figure S2

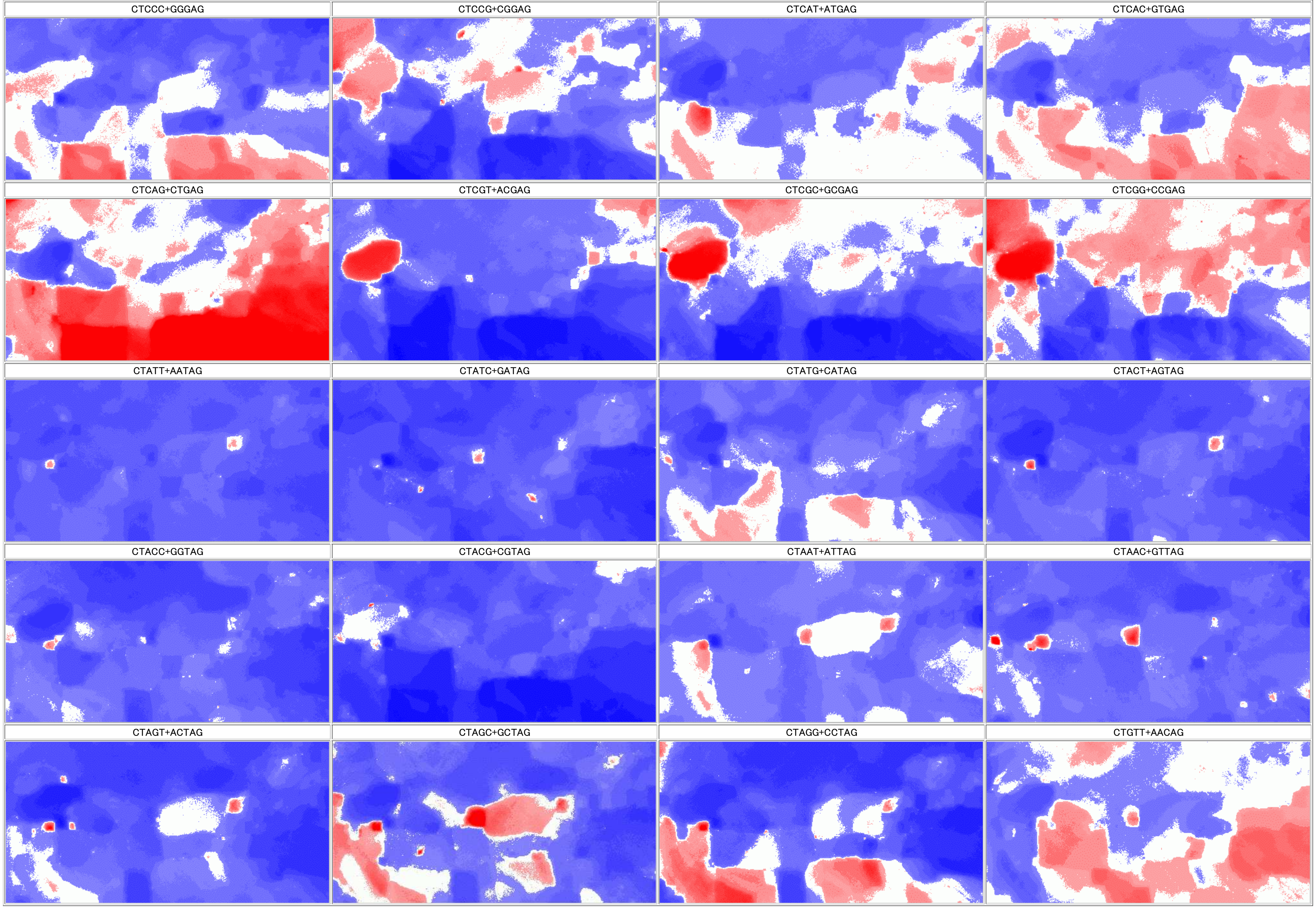

Supplementary Figure S2

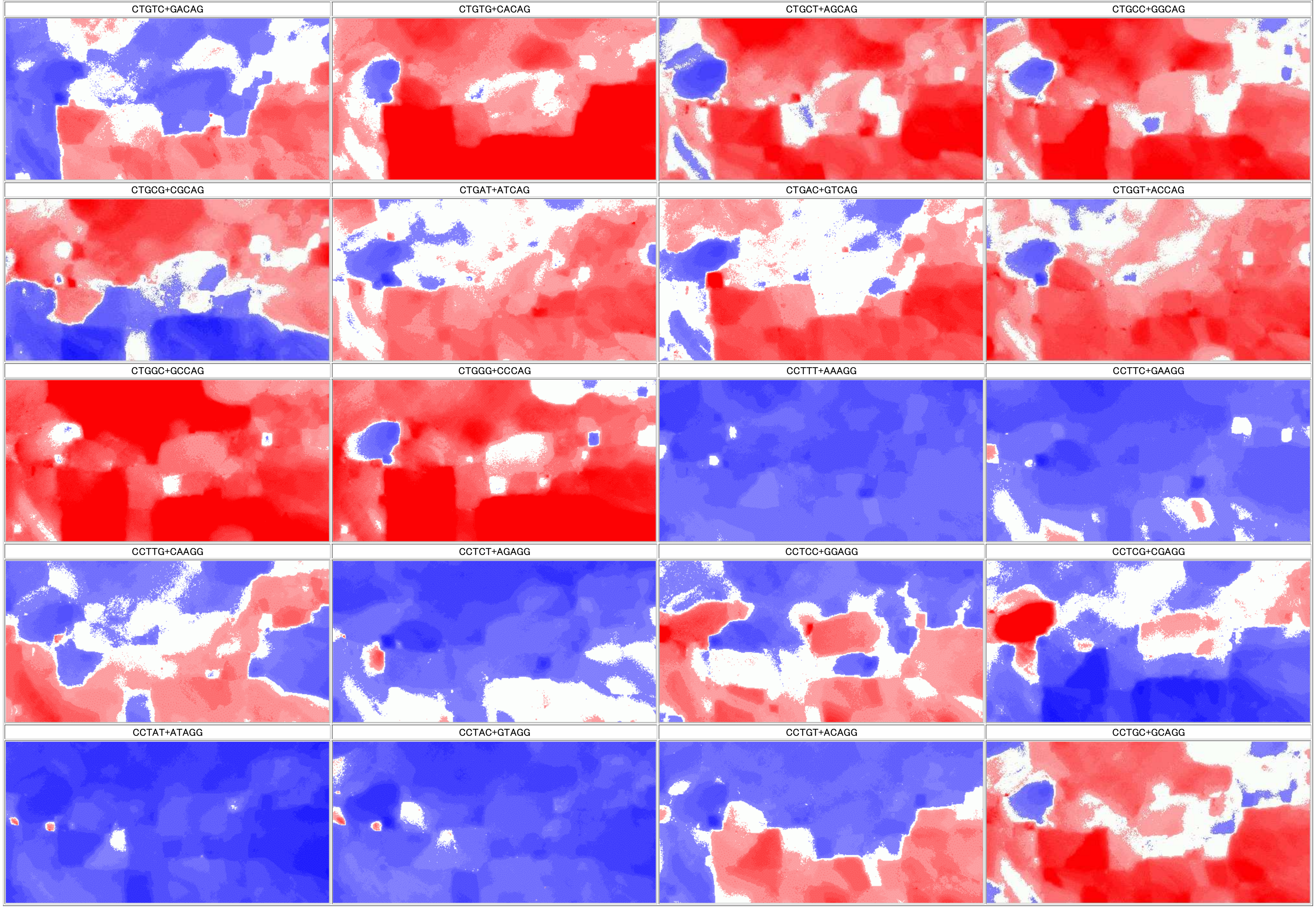

Supplementary Figure S2

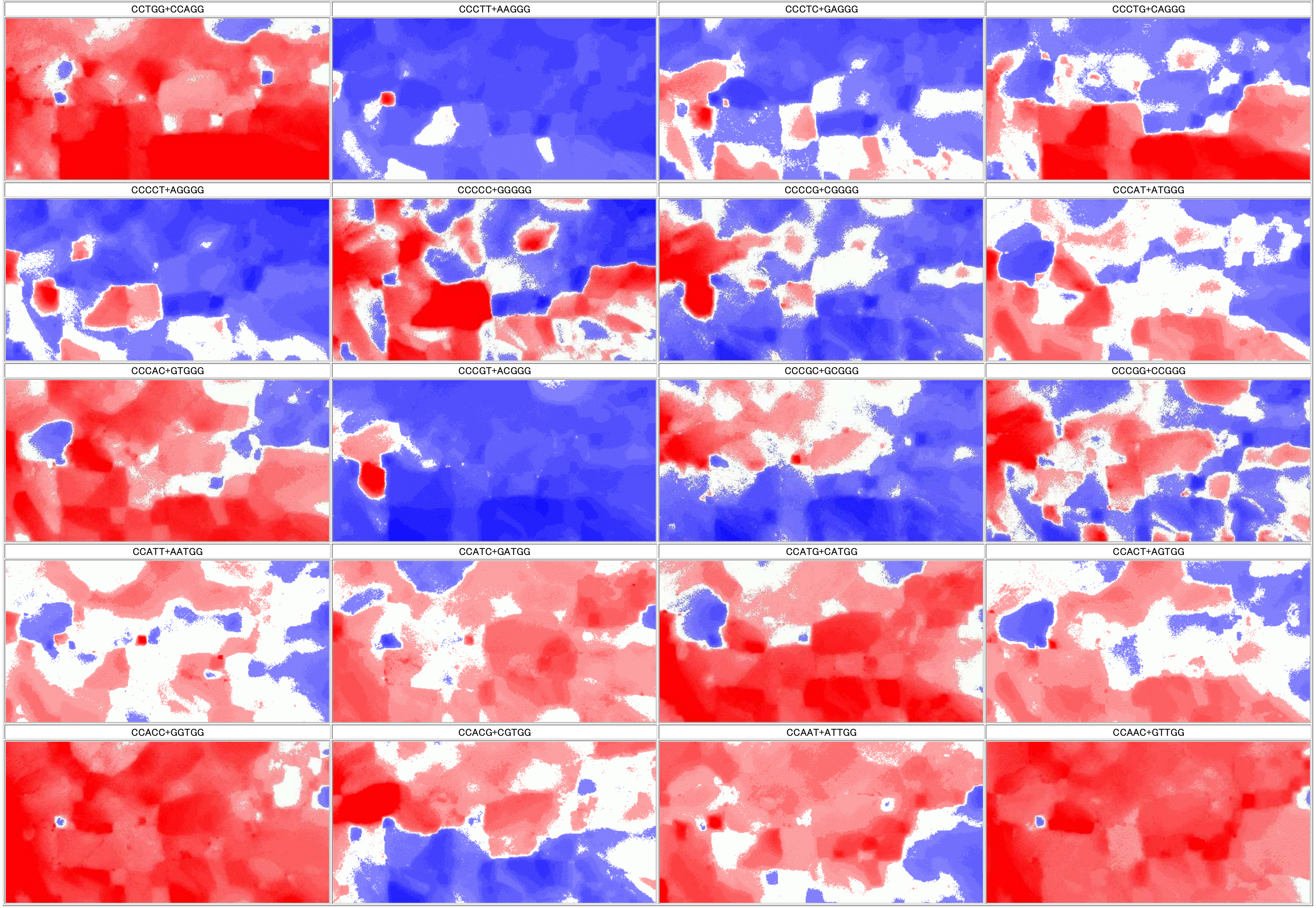

Supplementary Figure S2

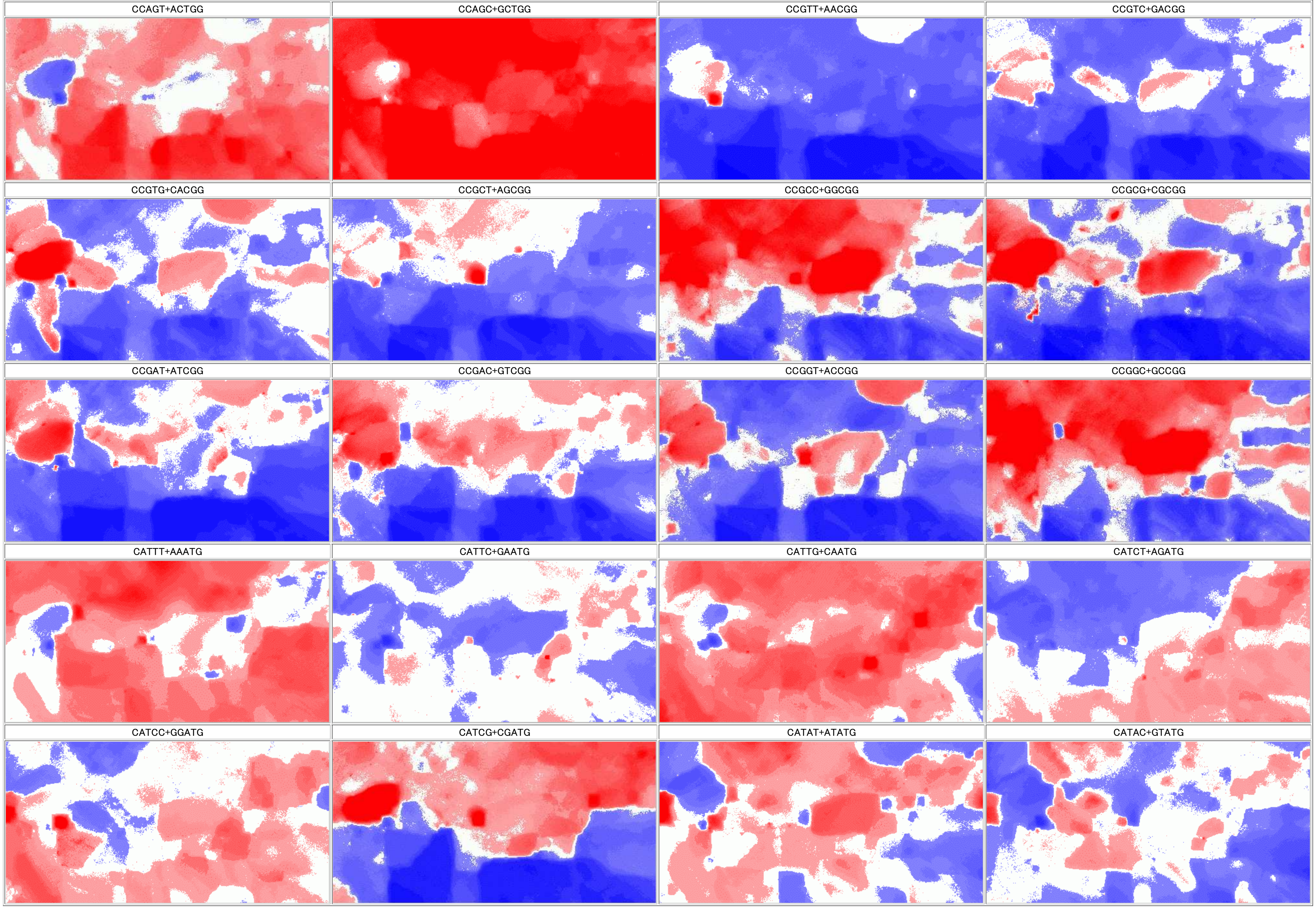

Supplementary Figure S2

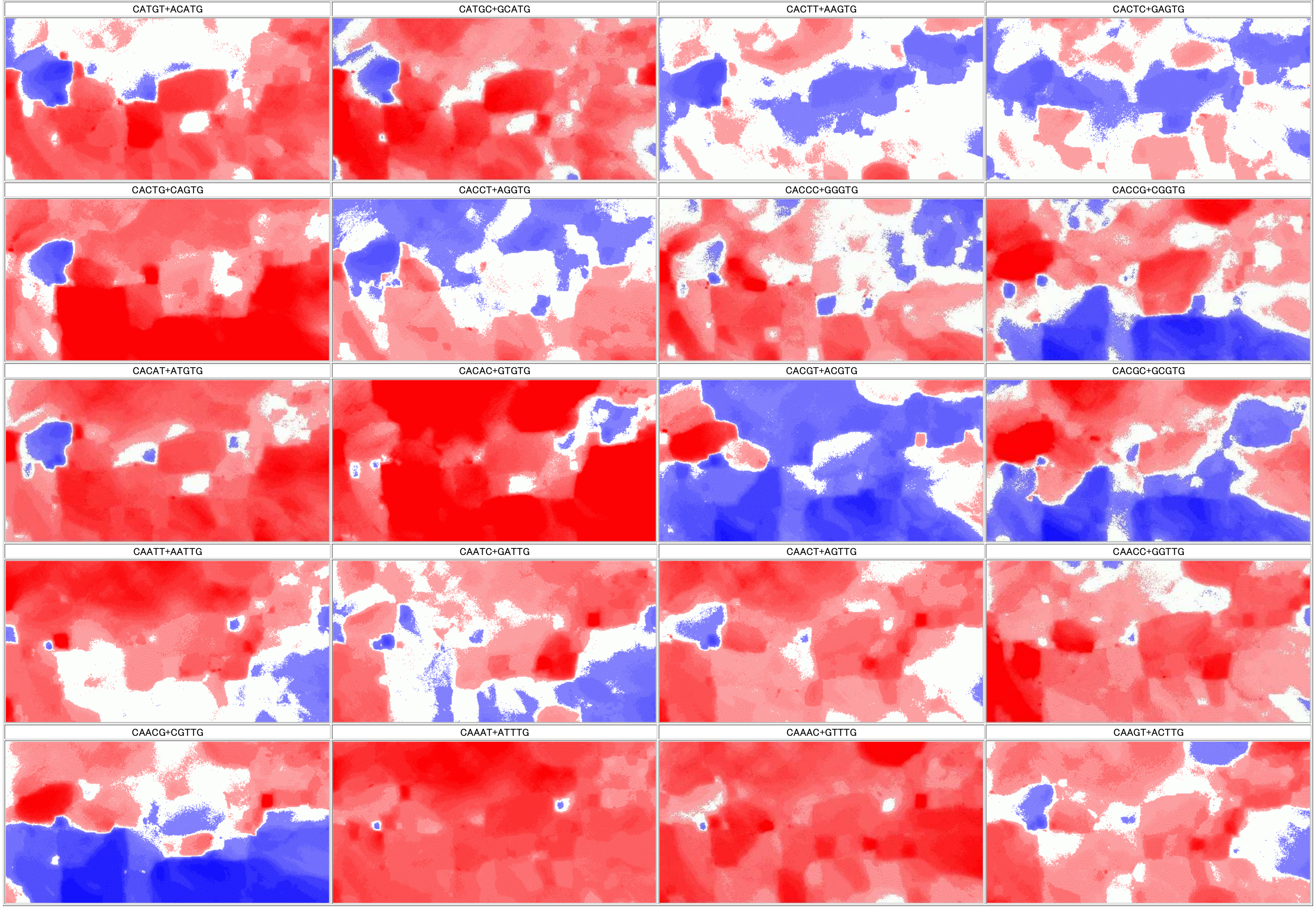

Supplementary Figure S2

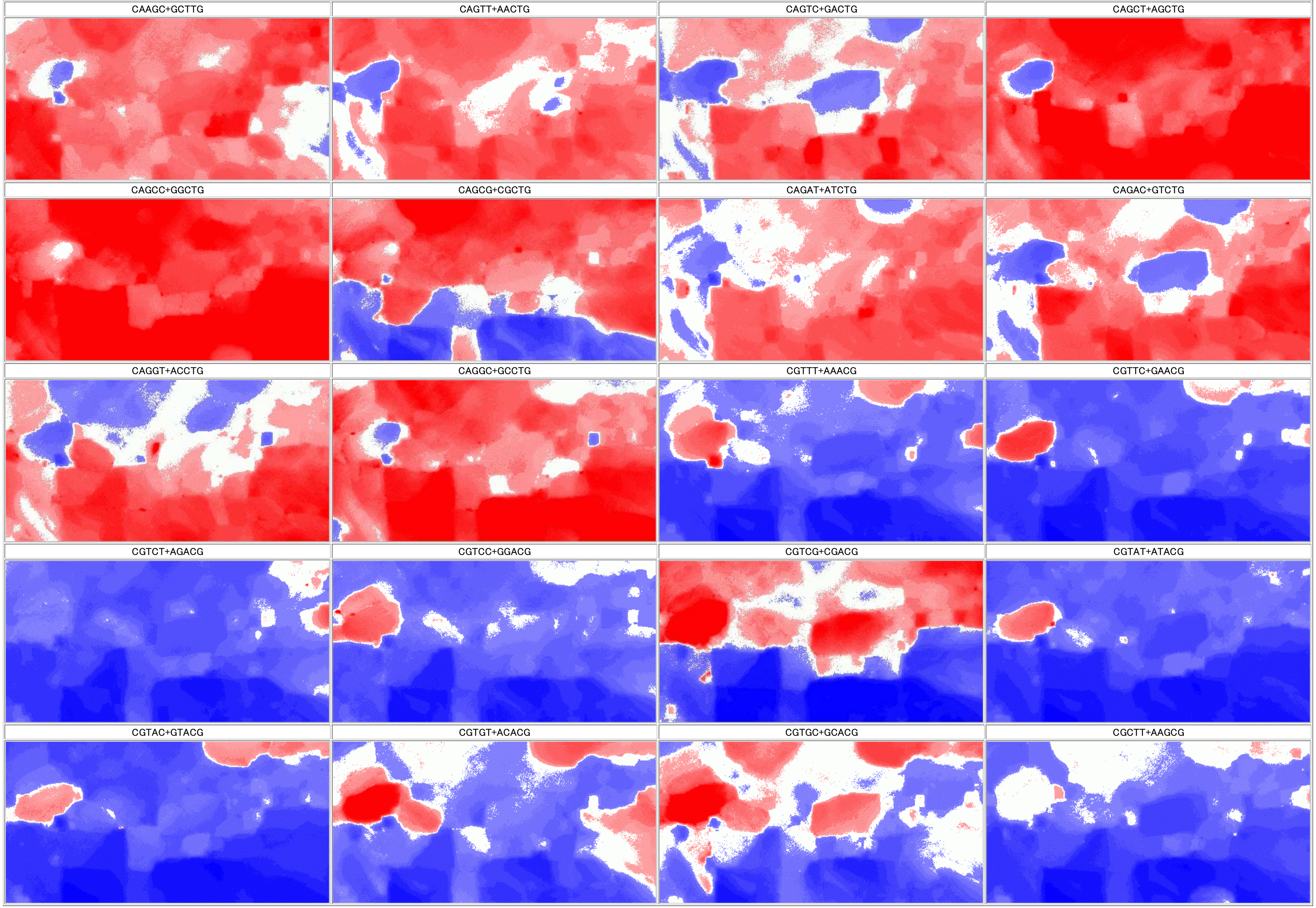

Supplementary Figure S2

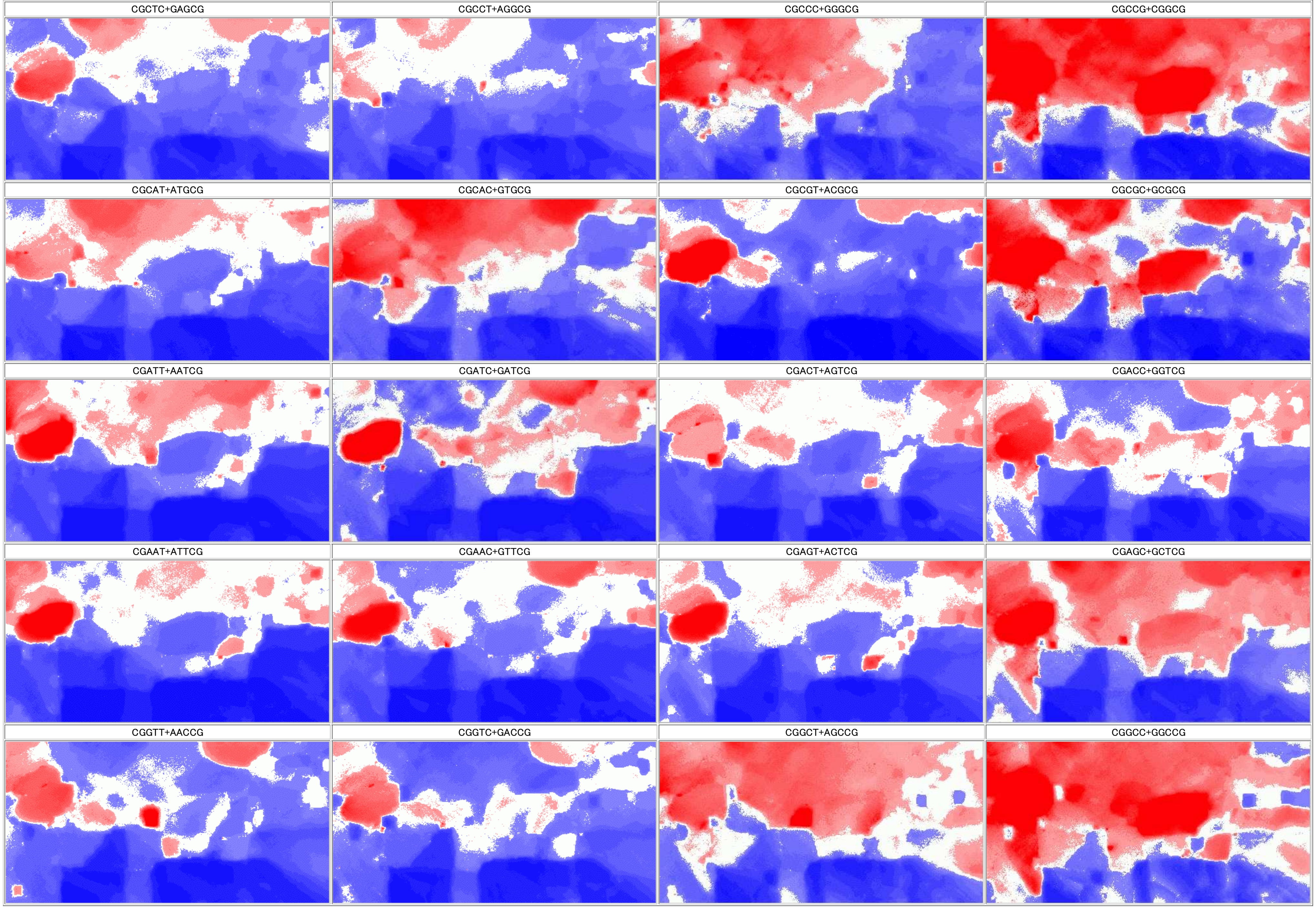

Supplementary Figure S2

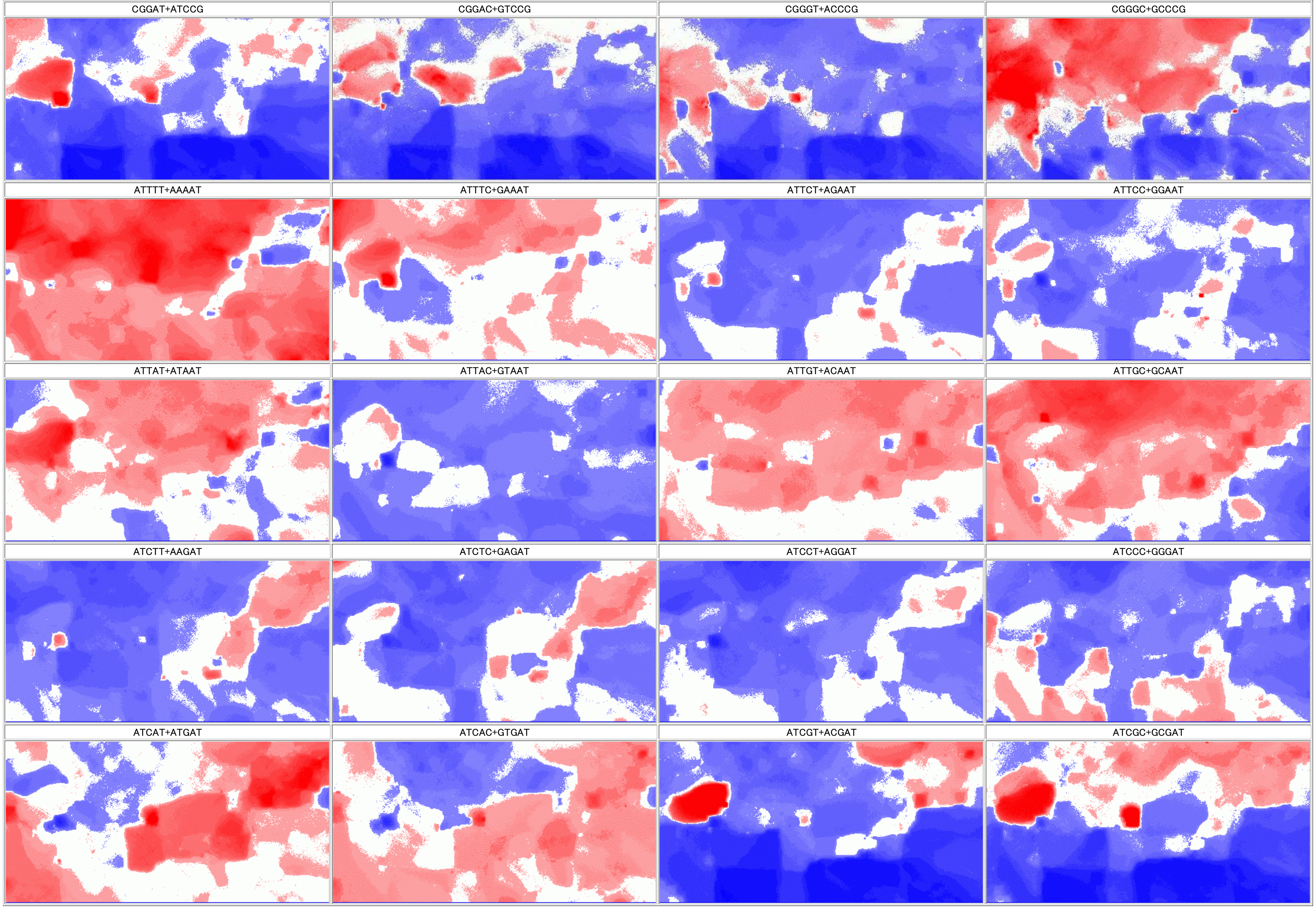

Supplementary Figure S2

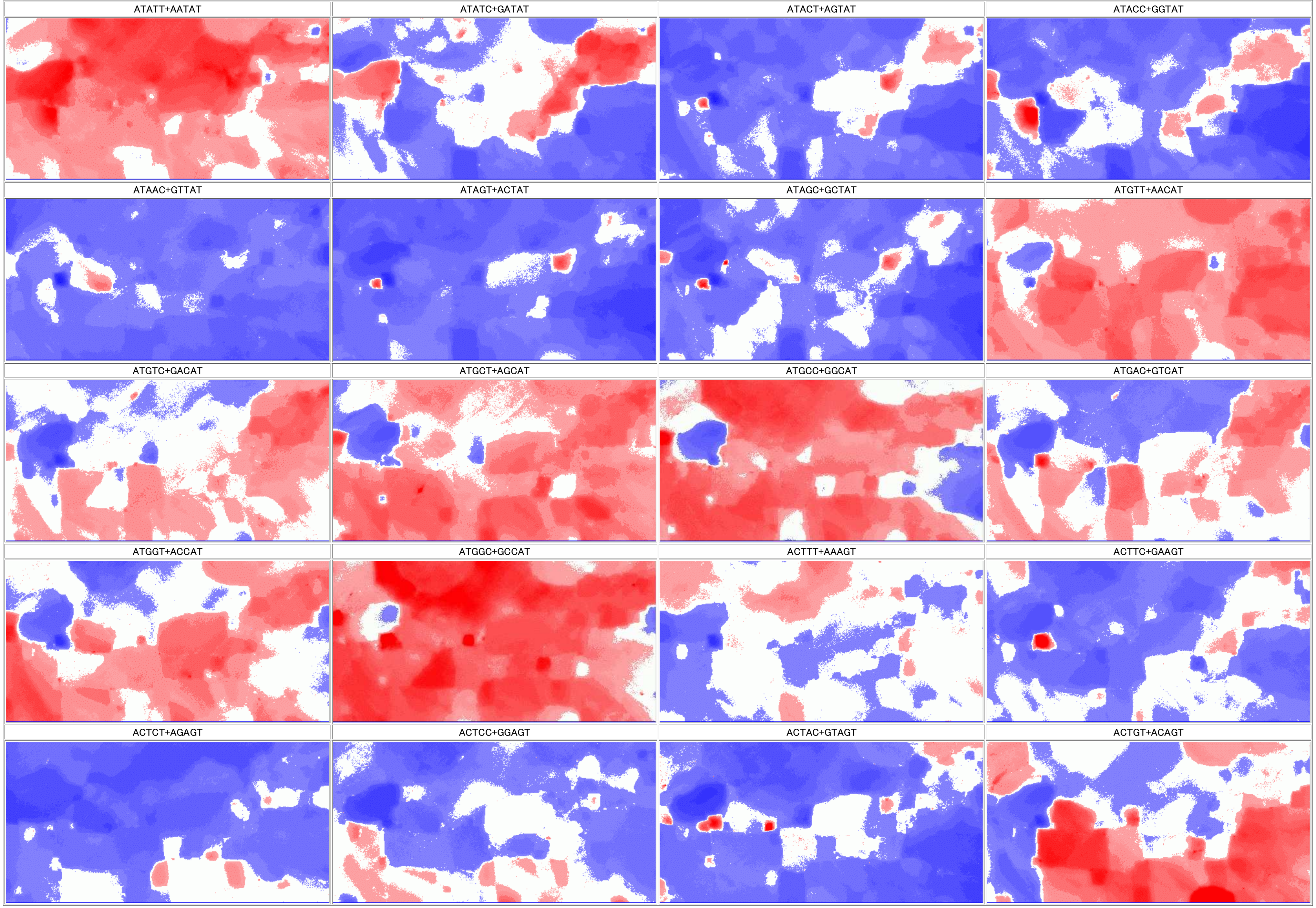

Supplementary Figure S2

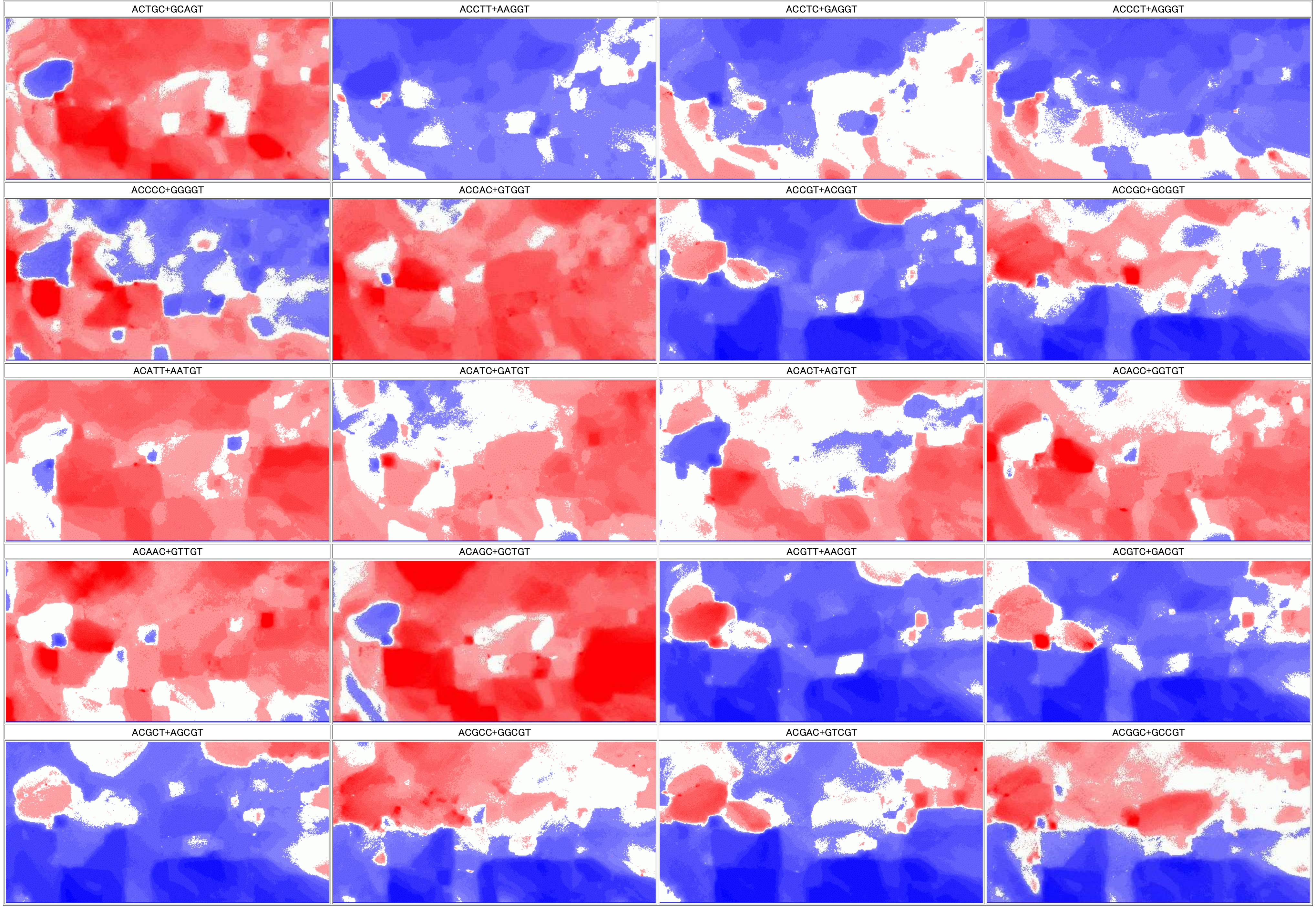

Supplementary Figure S2

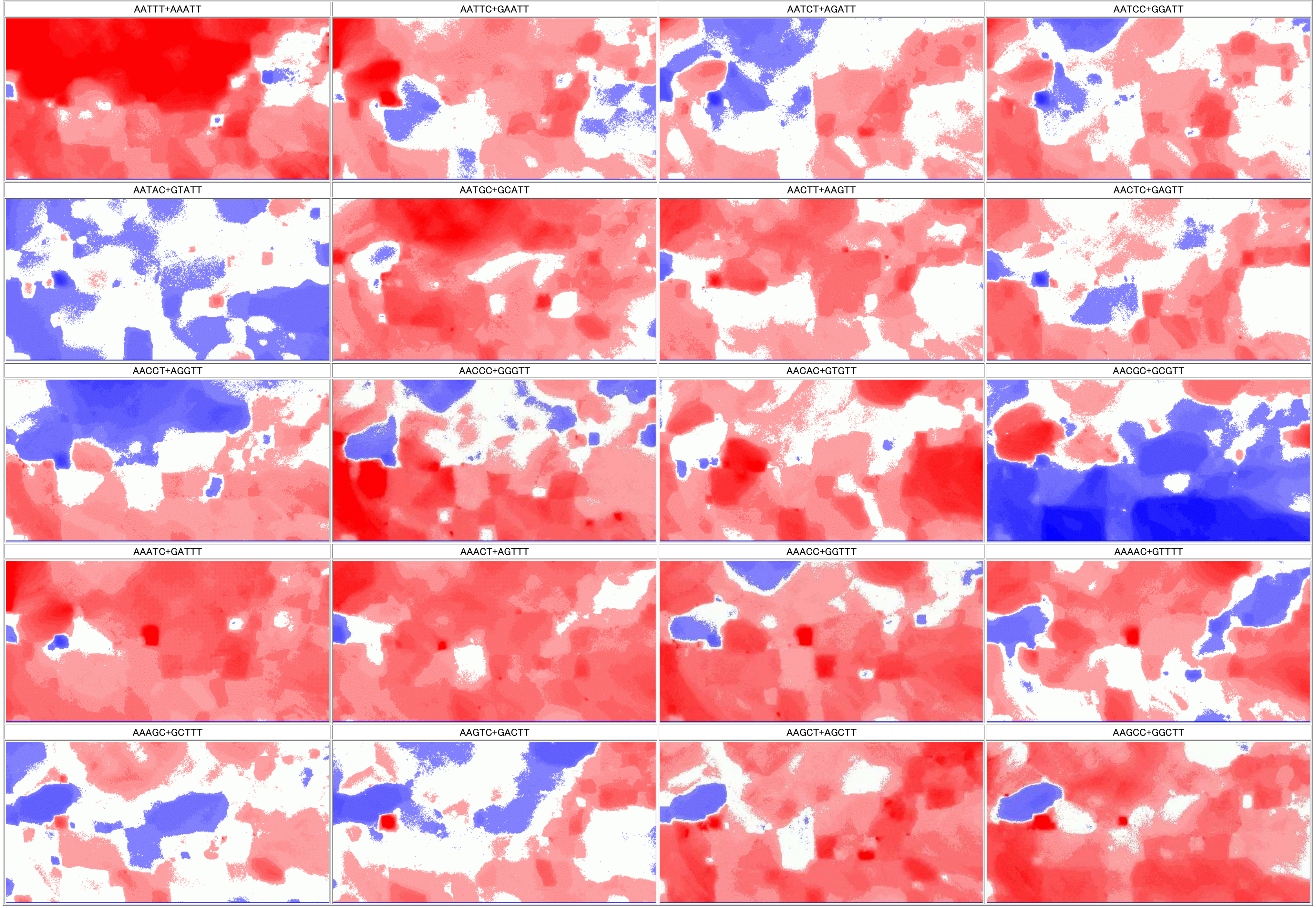

Supplementary Figure S2

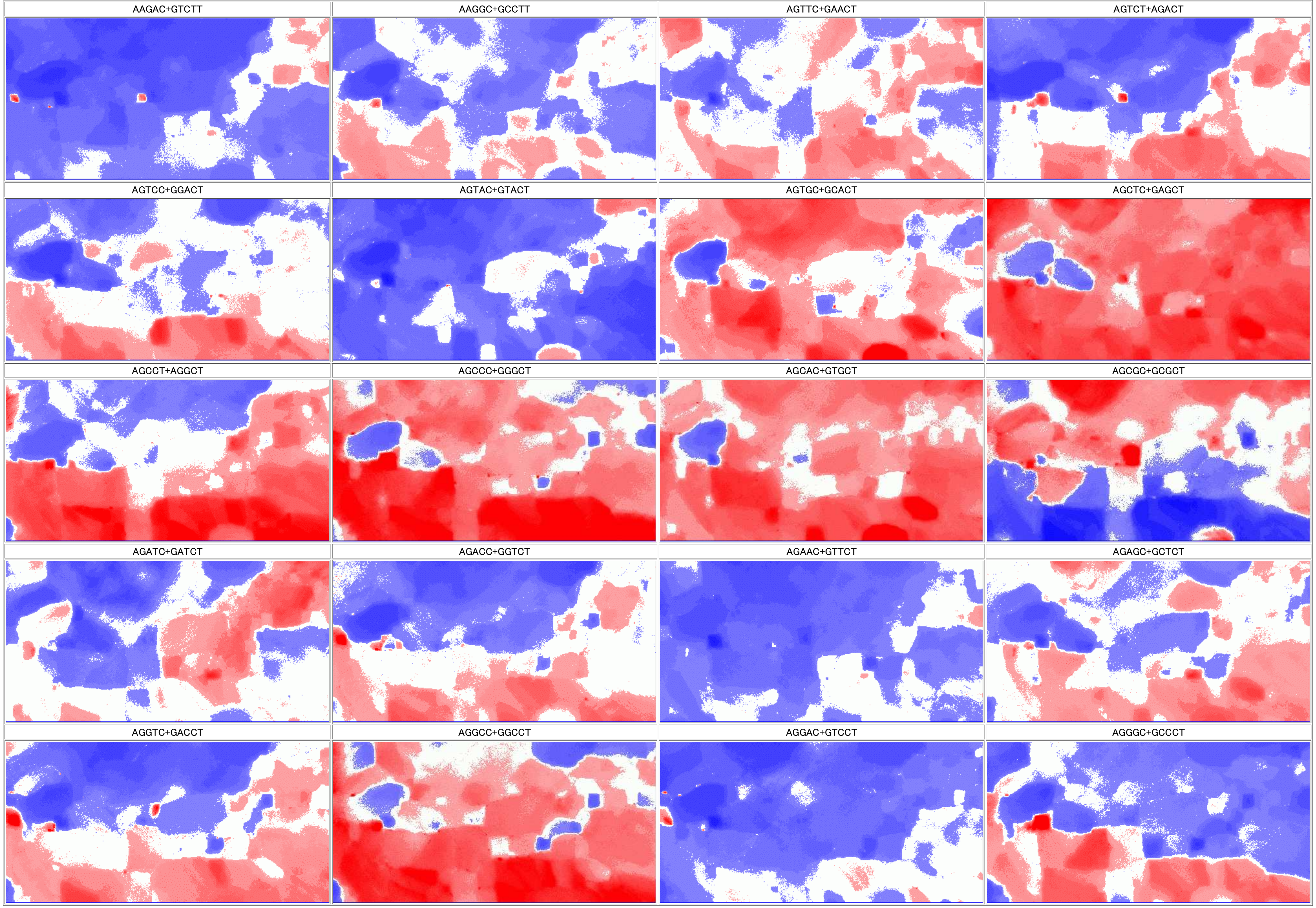

Supplementary Figure S2

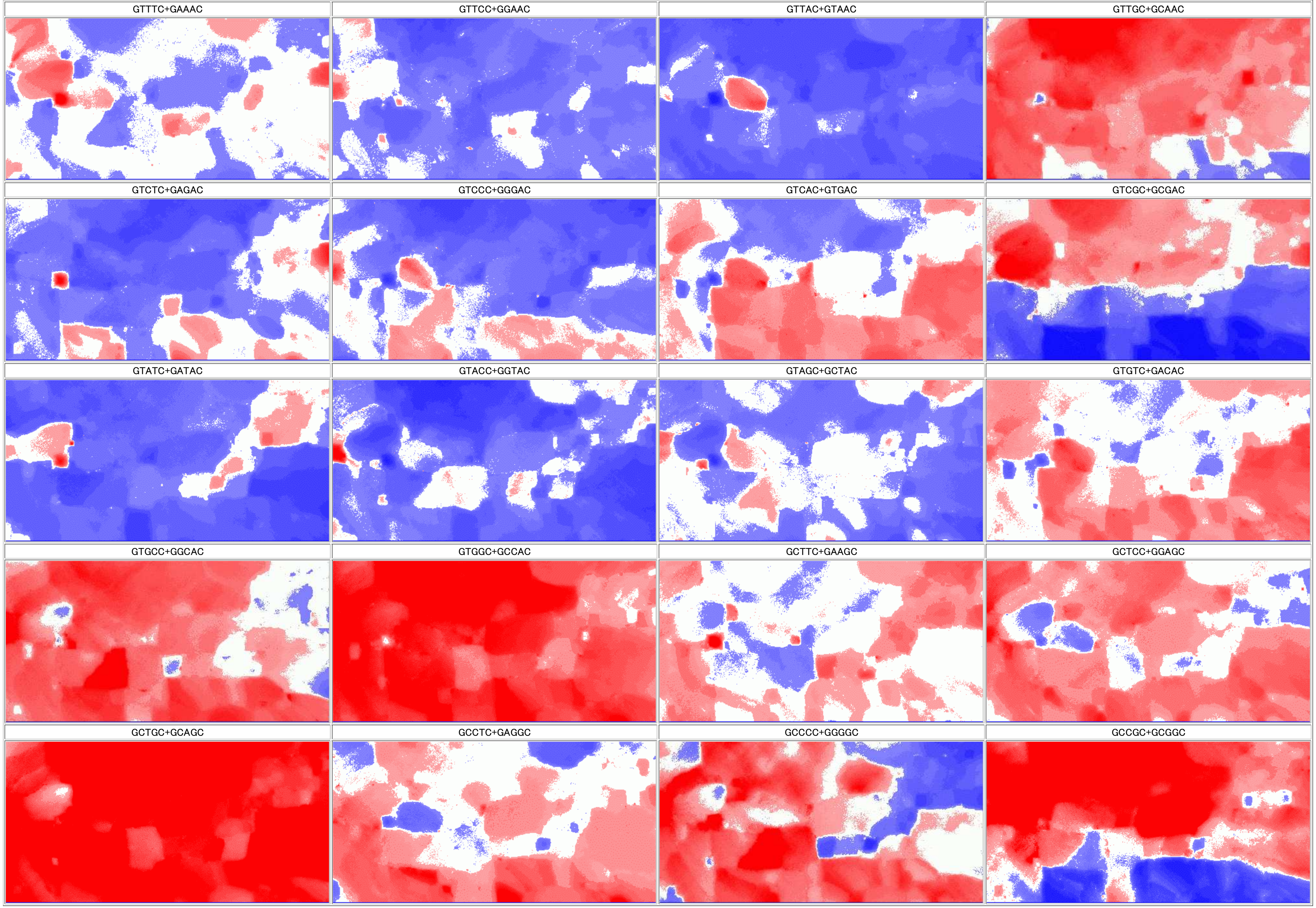

Supplementary Figure S2

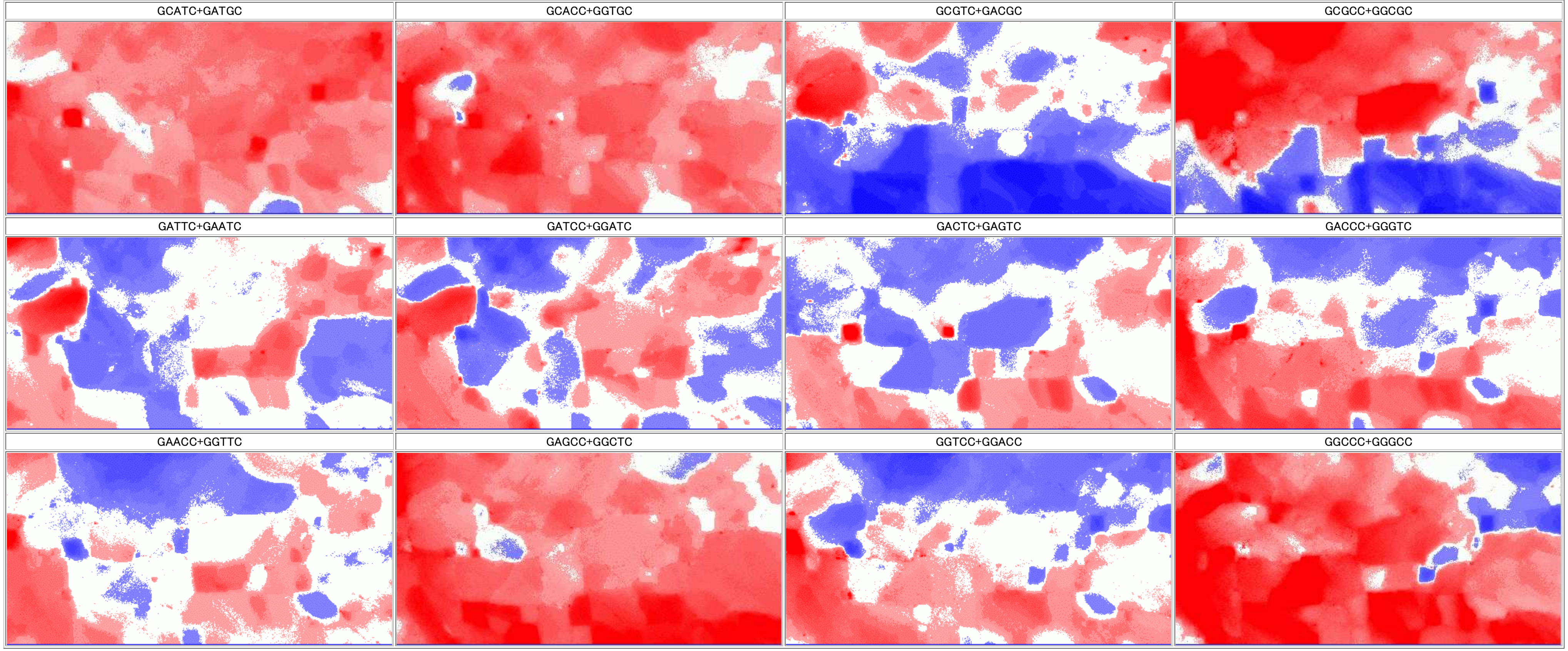

Supplementary Figure S2

Supplementary Figure S3.
Level of each pair of complimentary pentanucleotide in 100-kb DegePenta-BLSOM for the overlapping 100-kb sequences of 12 Drosophila genomes.

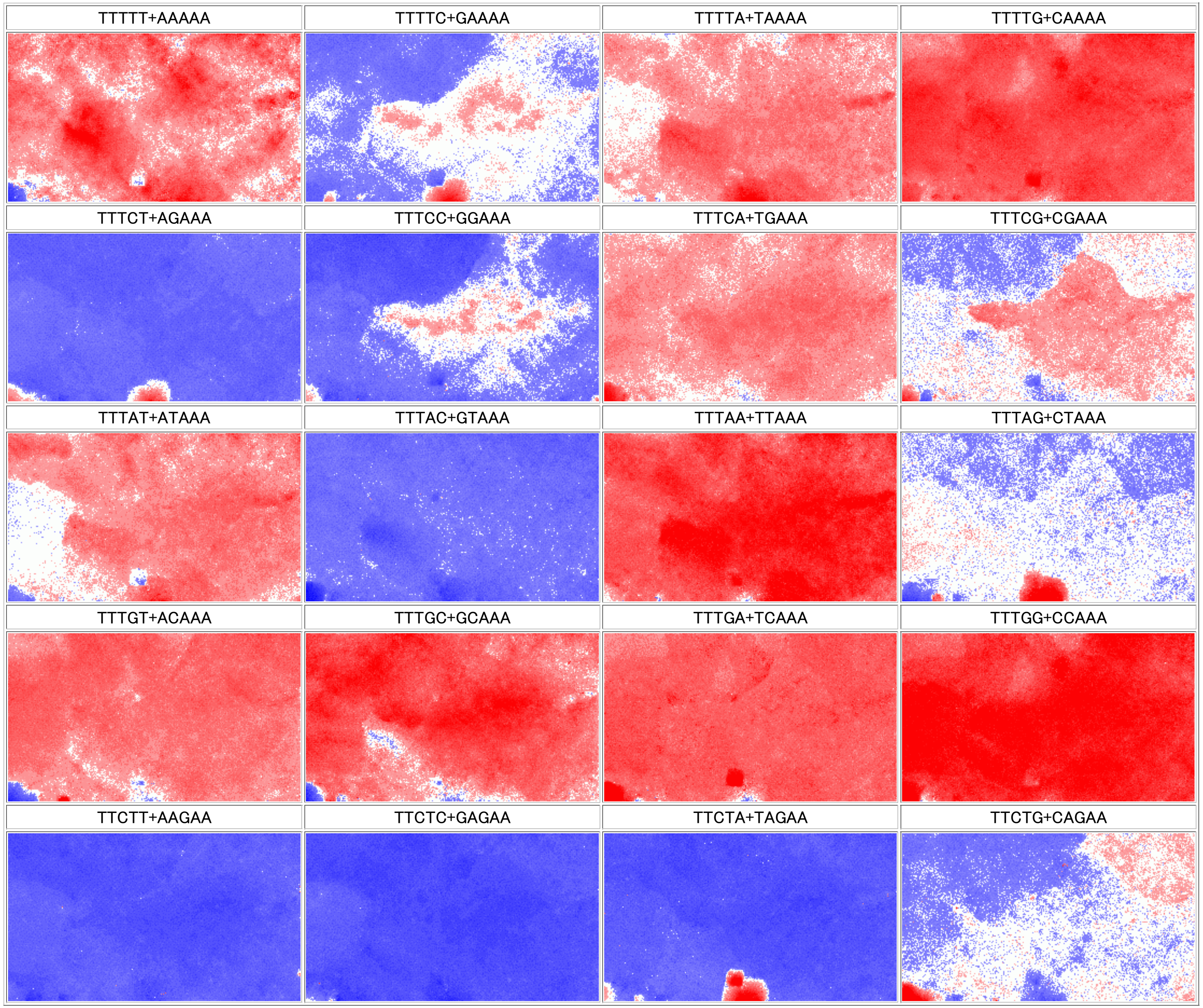

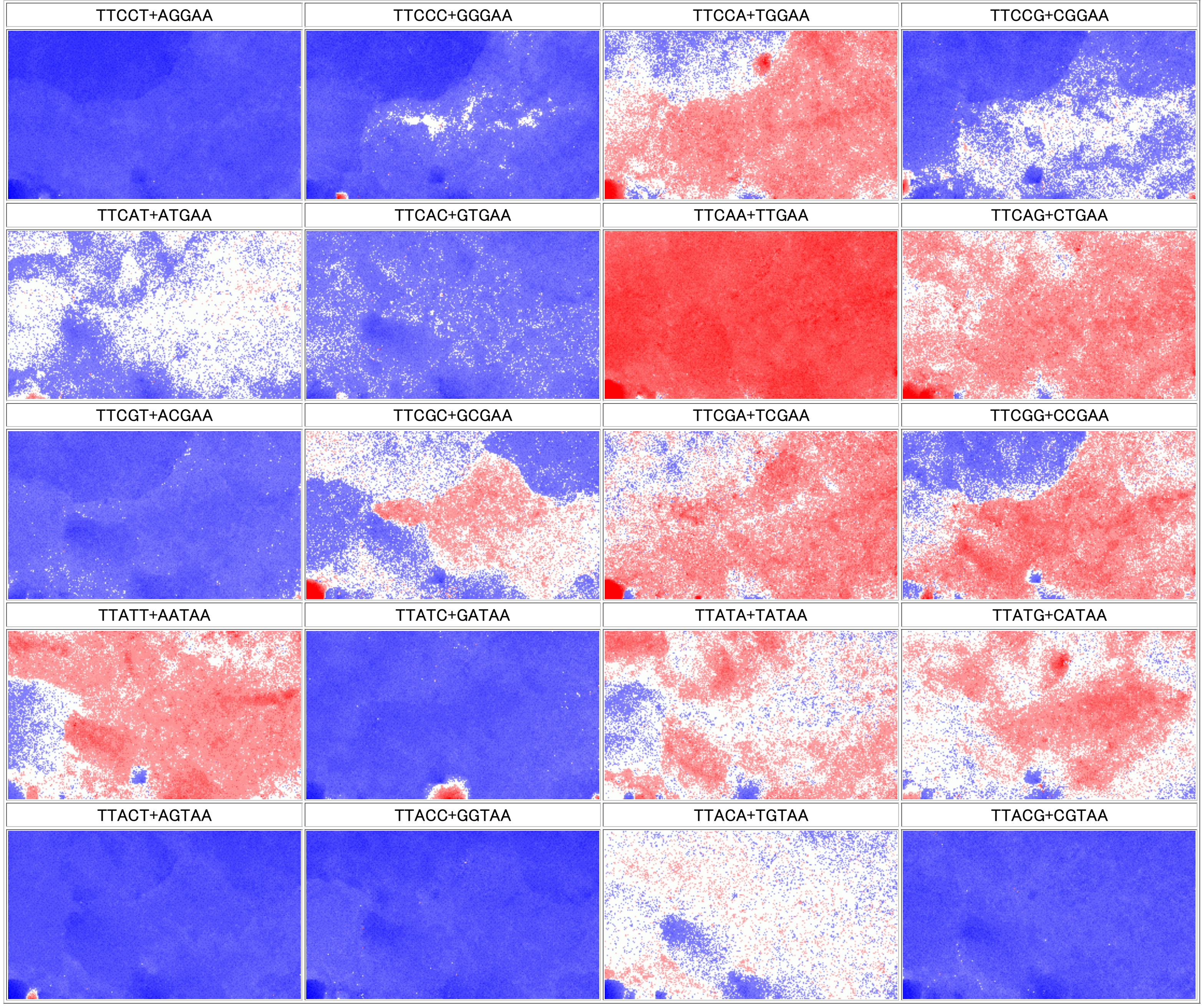

Supplementary Figure S3

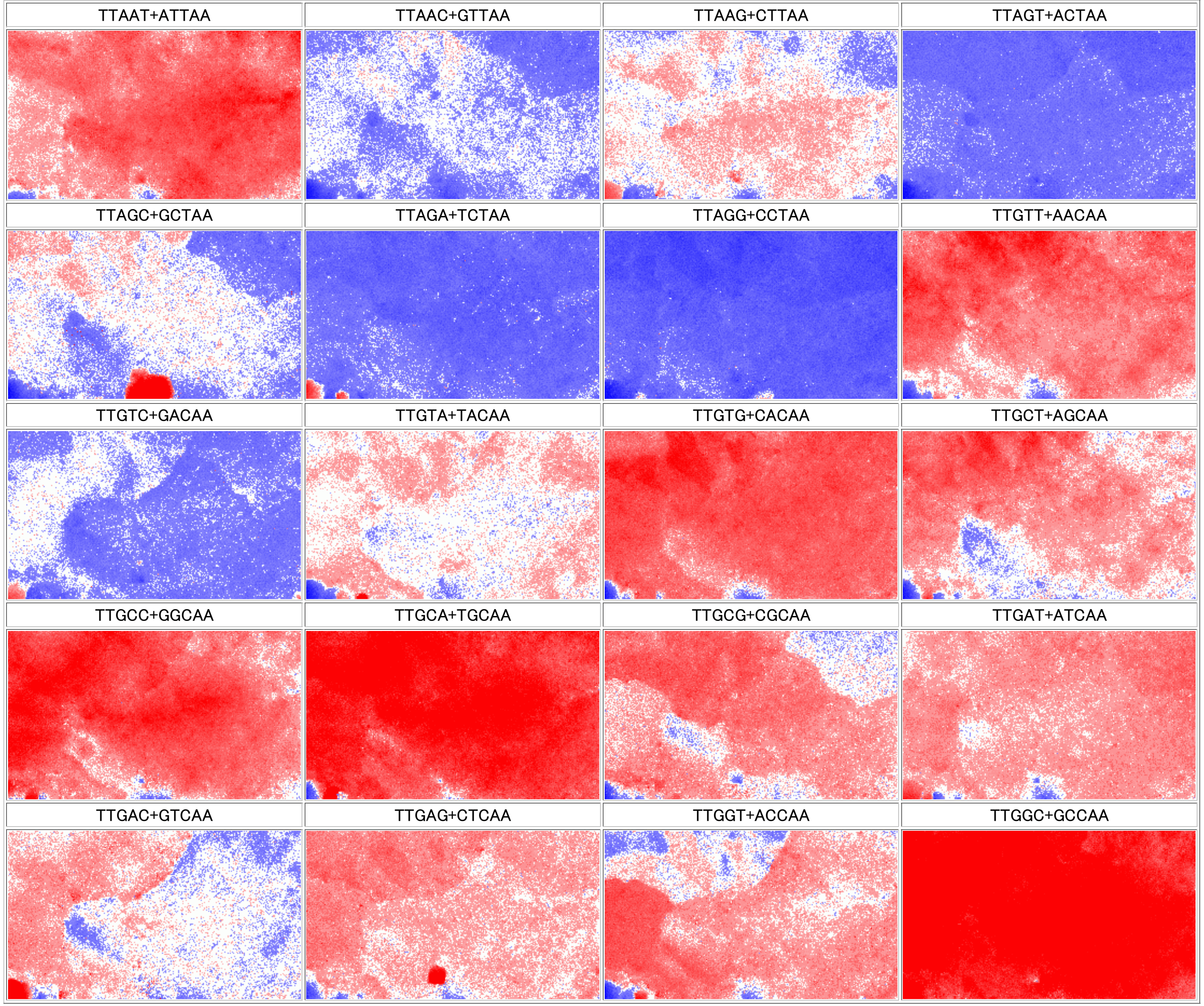

Supplementary Figure S3

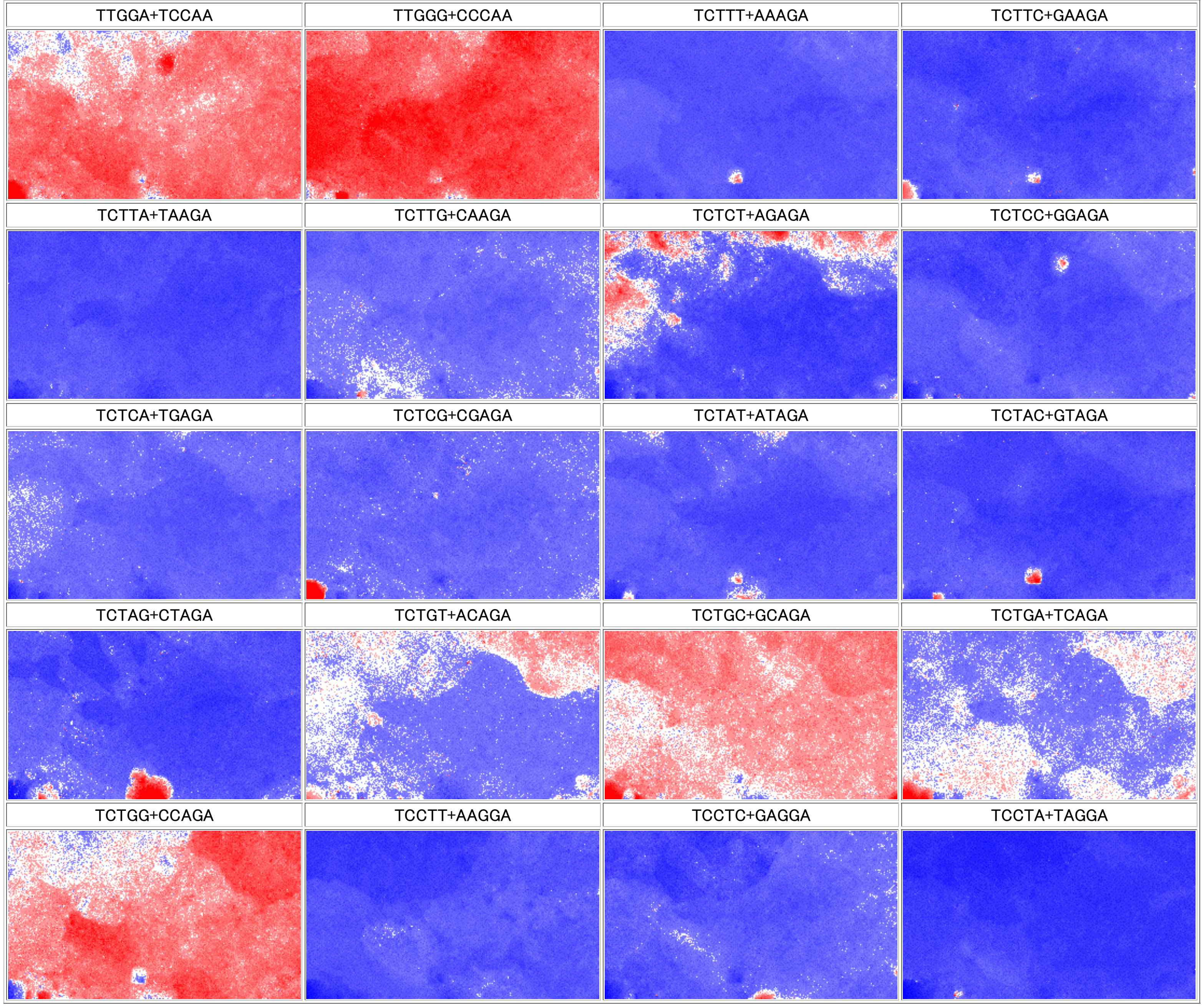

Supplementary Figure S3

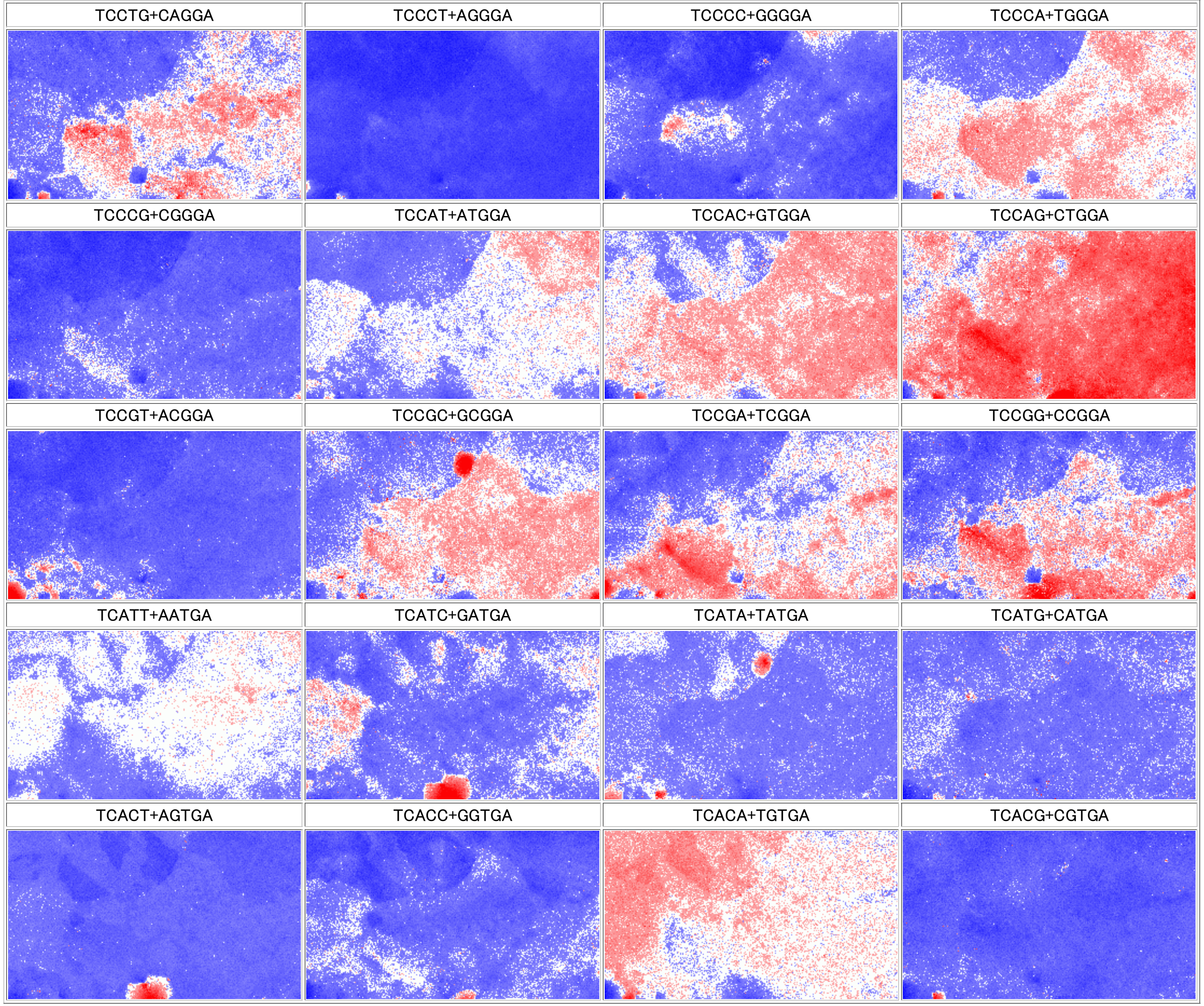

Supplementary Figure S3

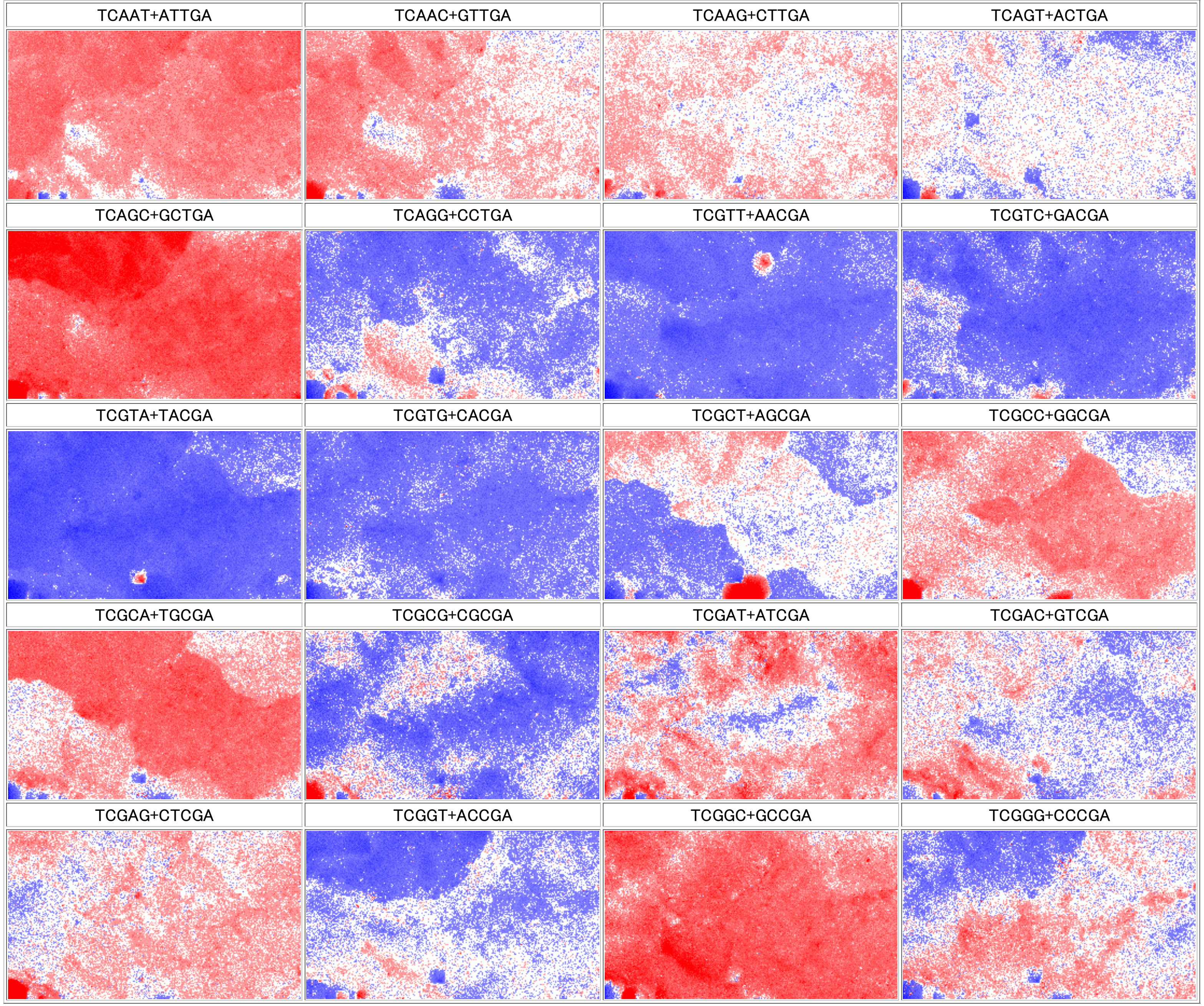

Supplementary Figure S3

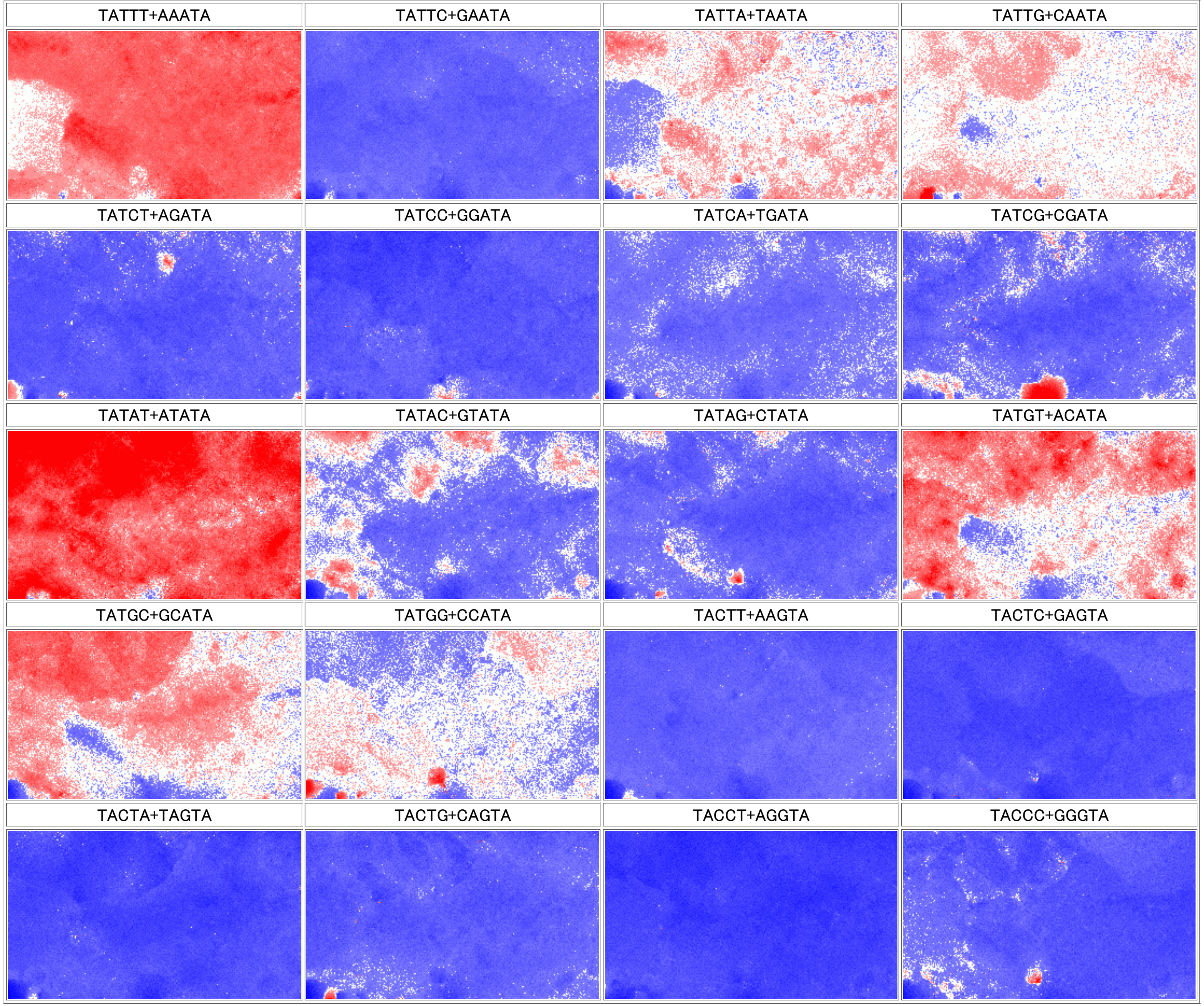

Supplementary Figure S3

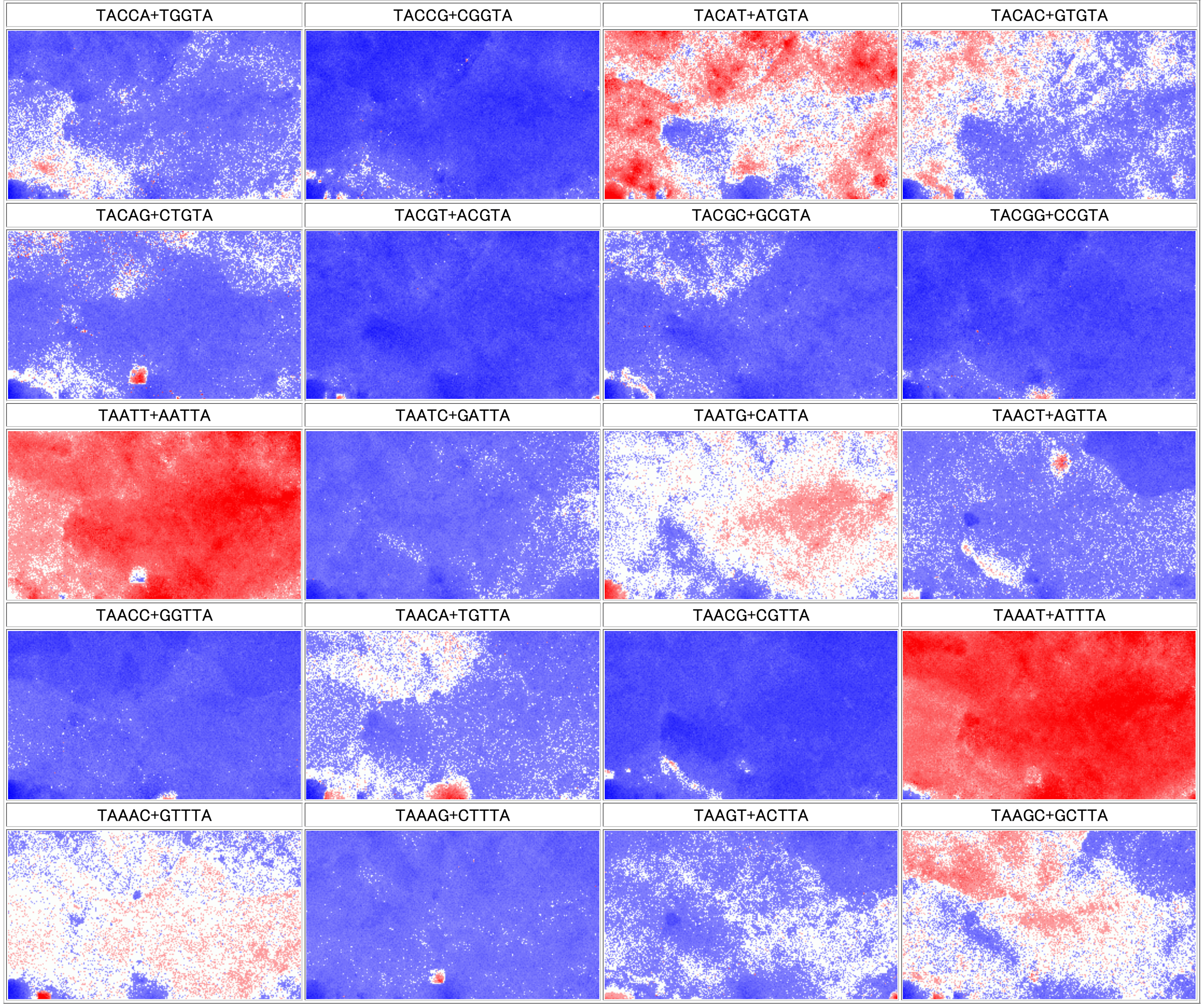

Supplementary Figure S3

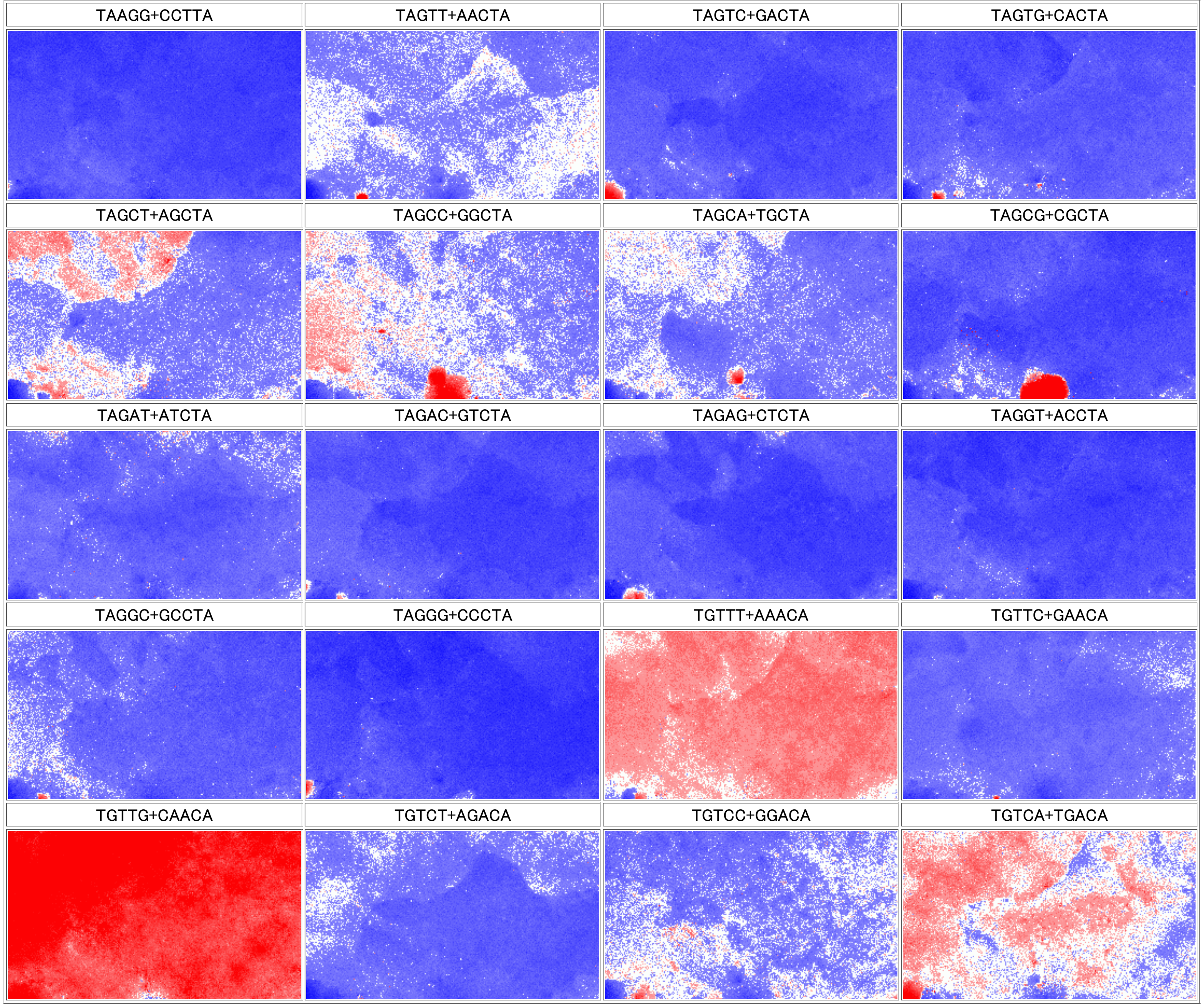

Supplementary Figure S3

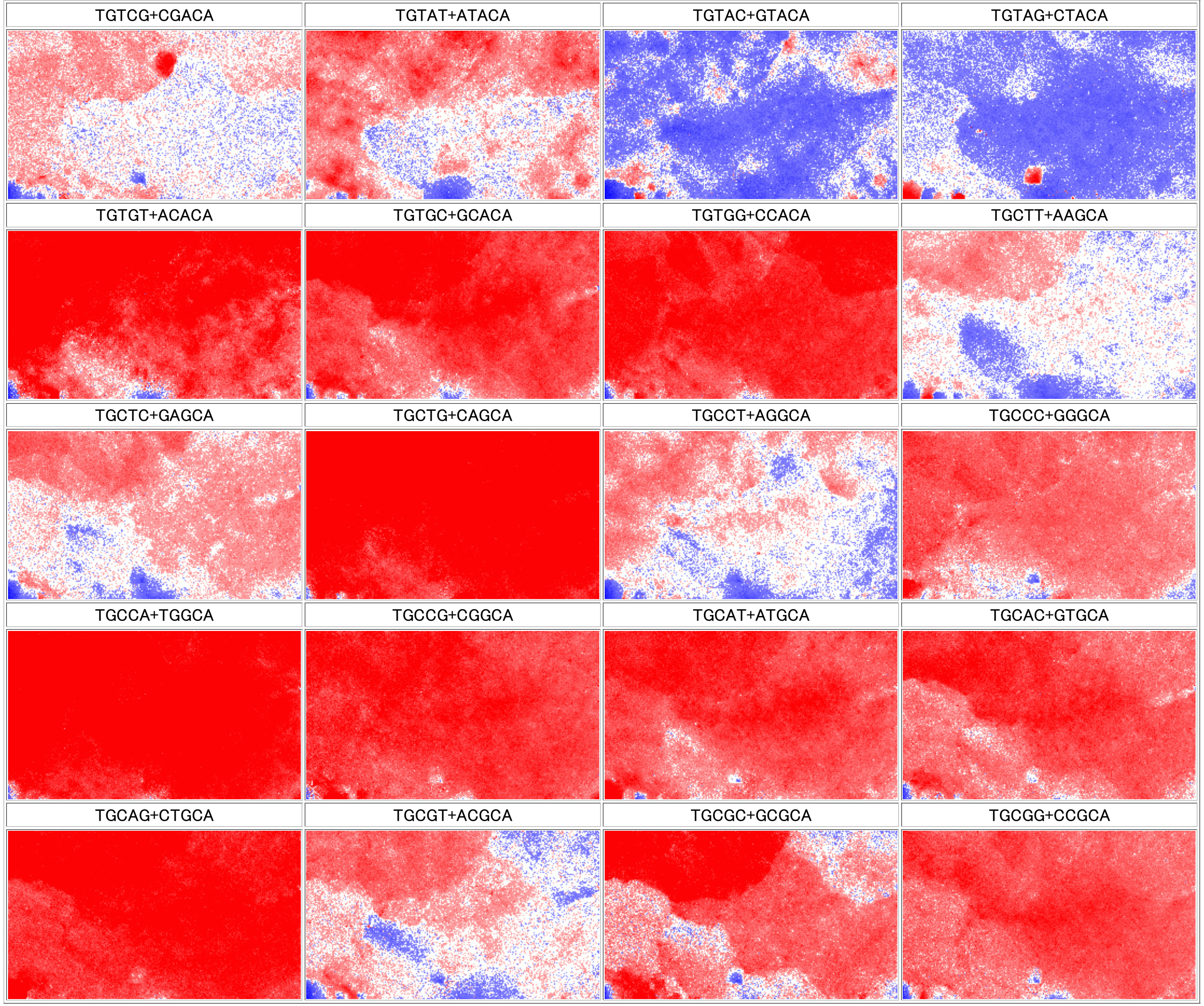

Supplementary Figure S3

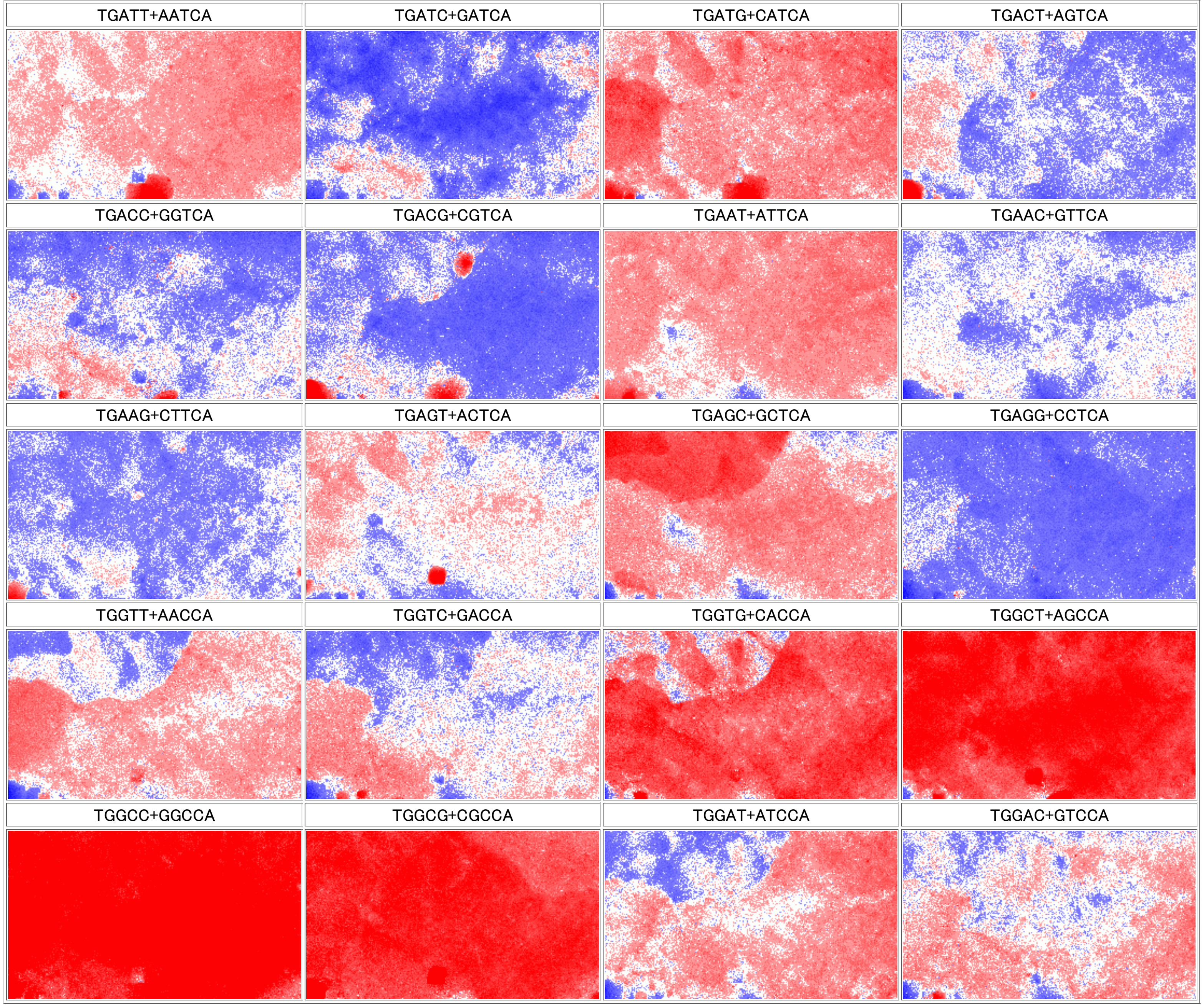

Supplementary Figure S3

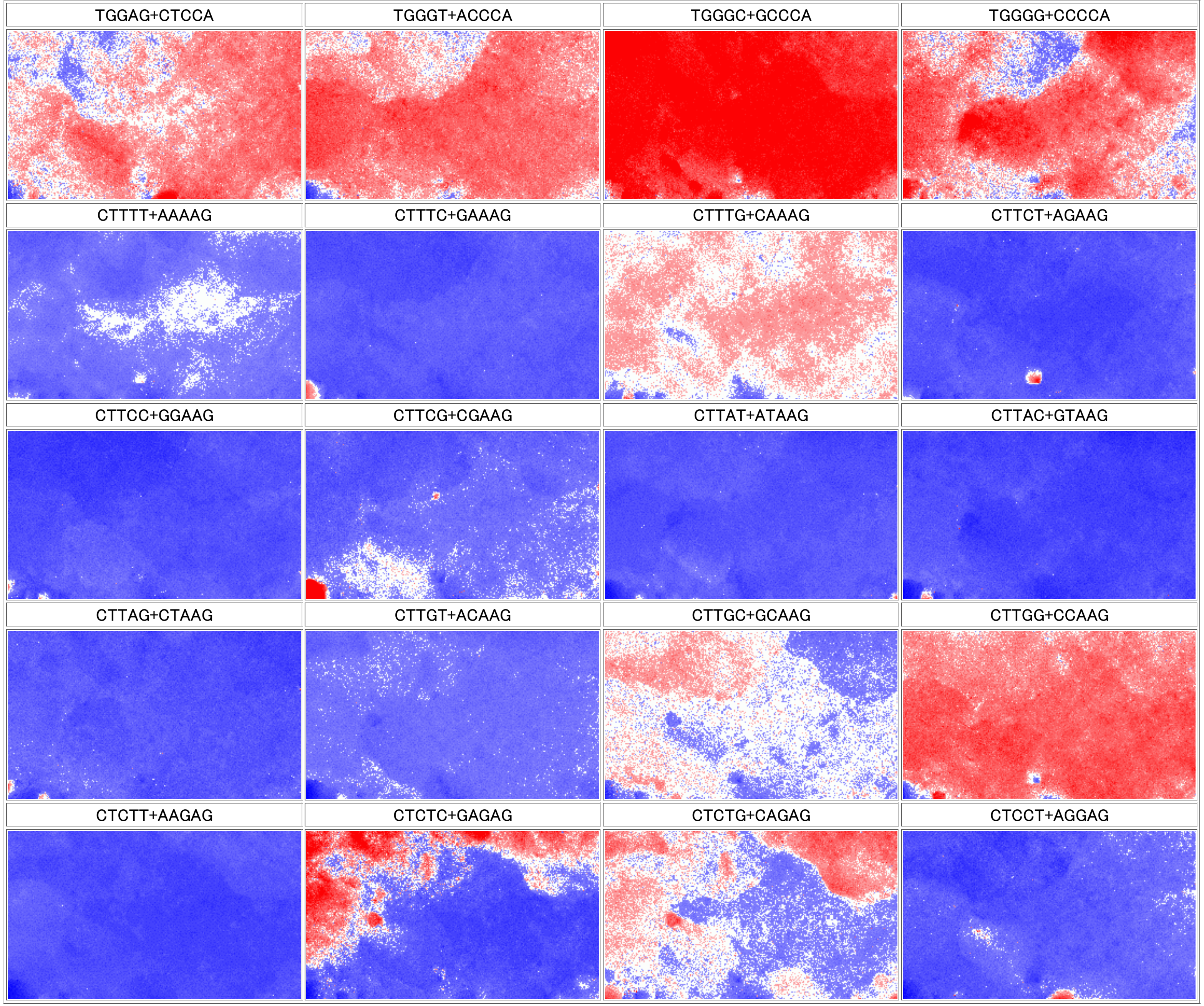

Supplementary Figure S3

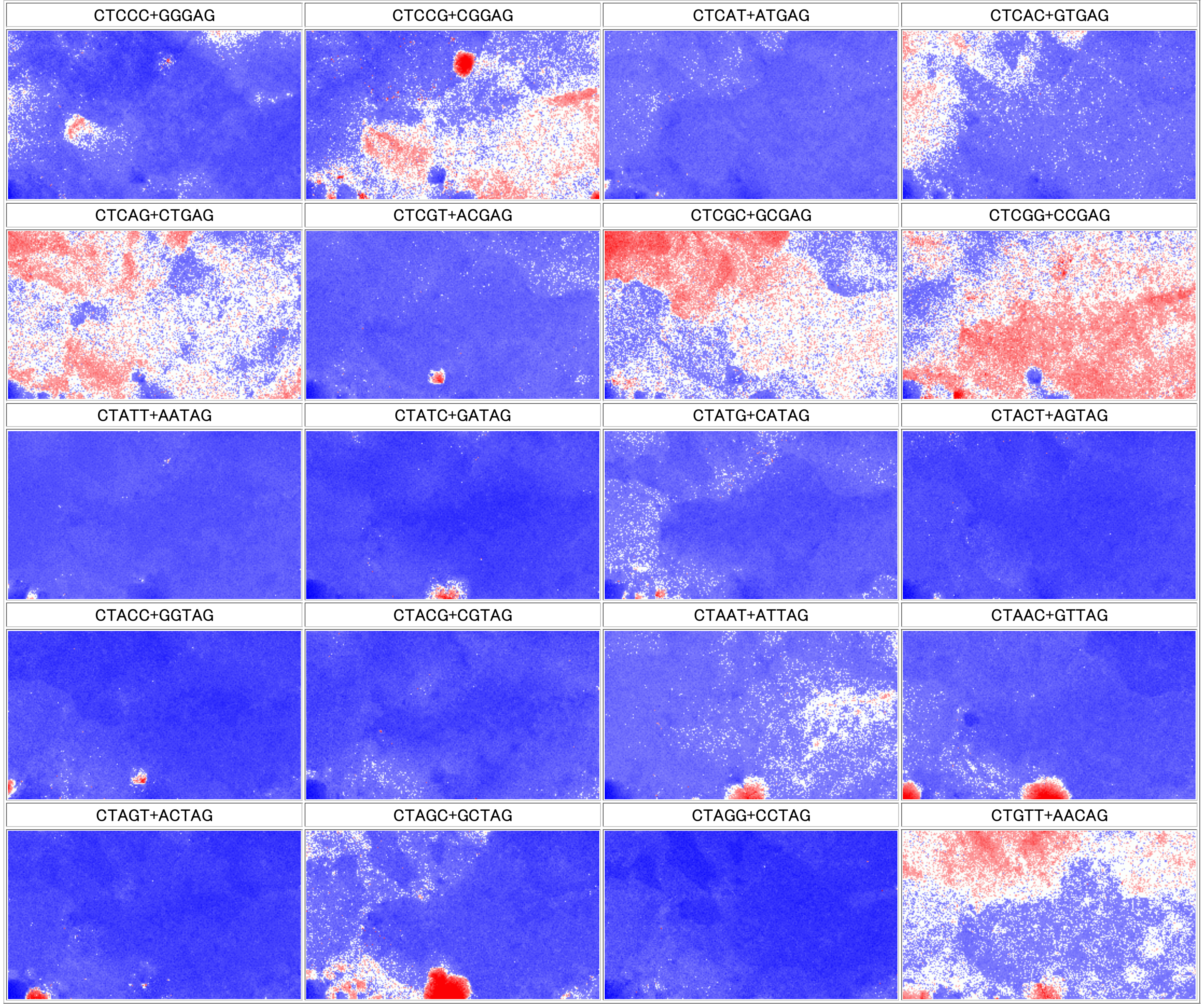

Supplementary Figure S3

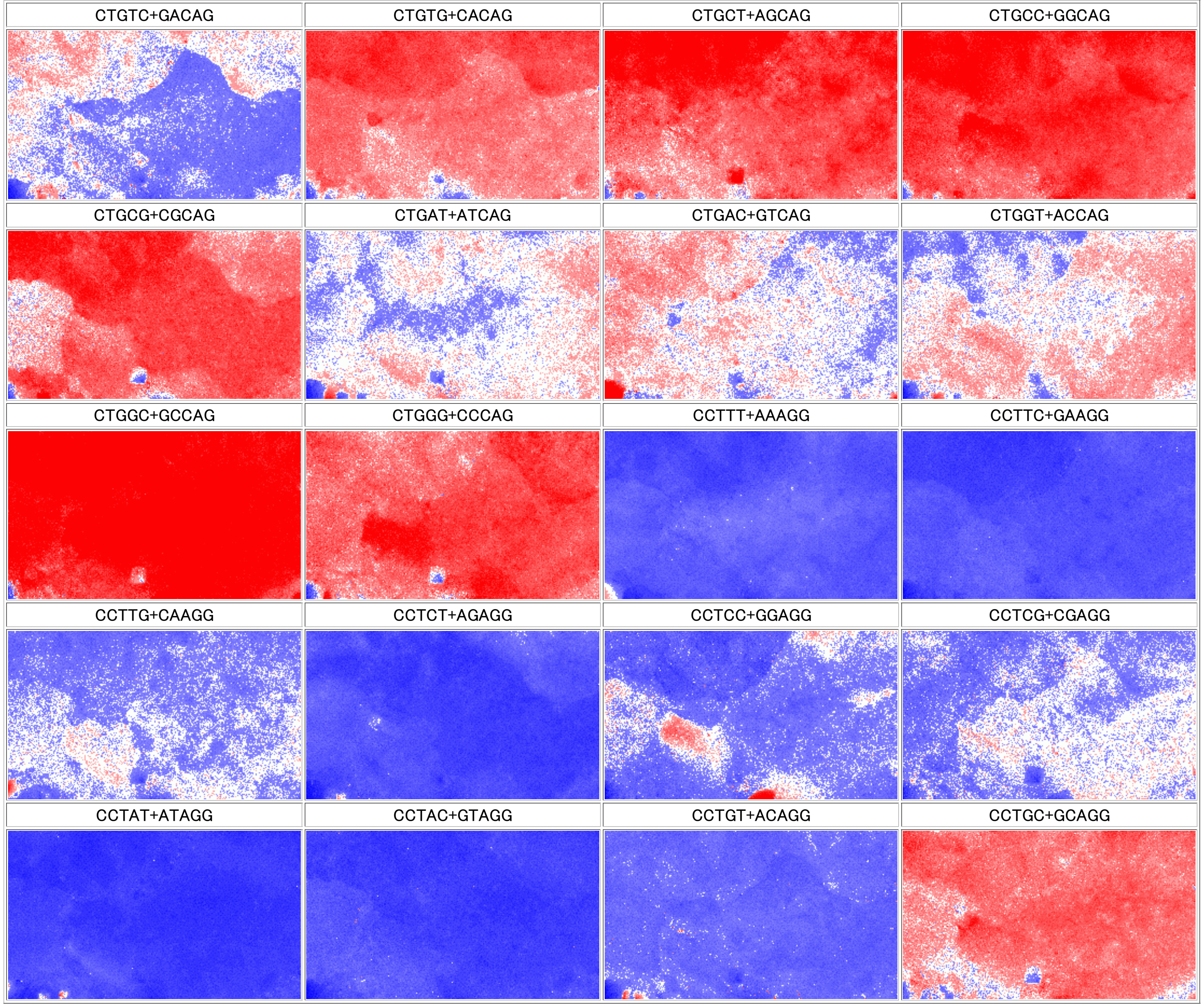

Supplementary Figure S3

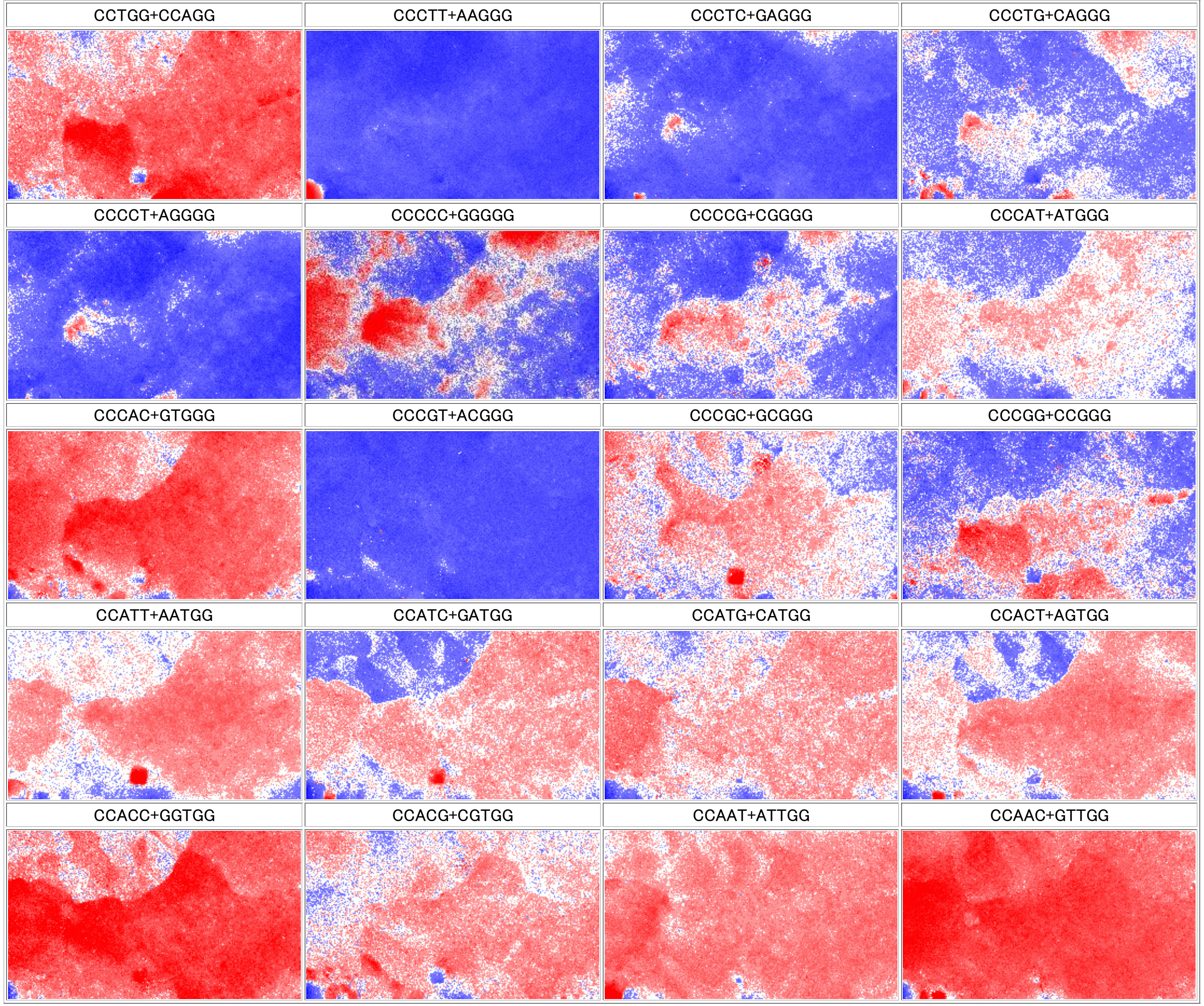

Supplementary Figure S3

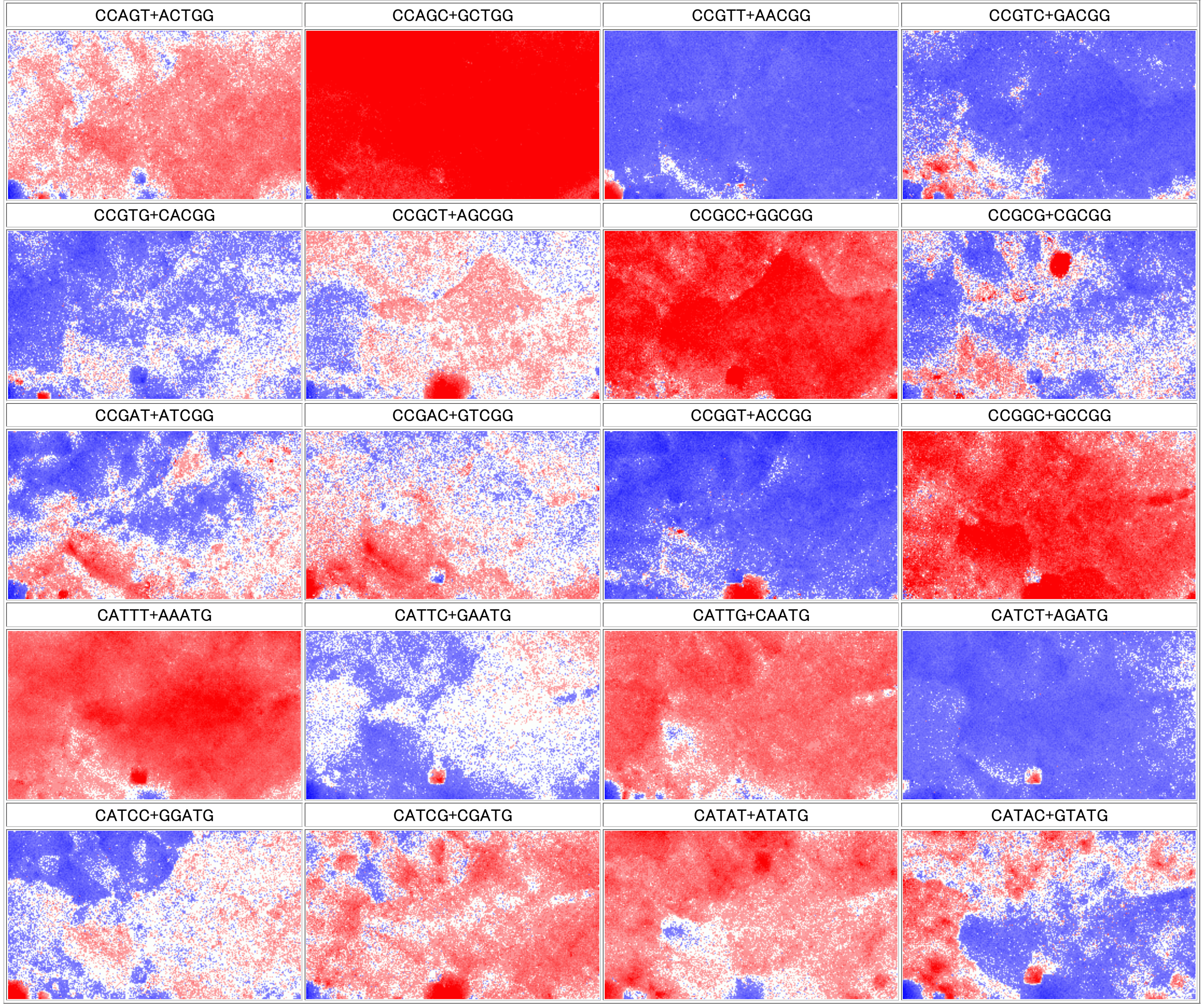

Supplementary Figure S3

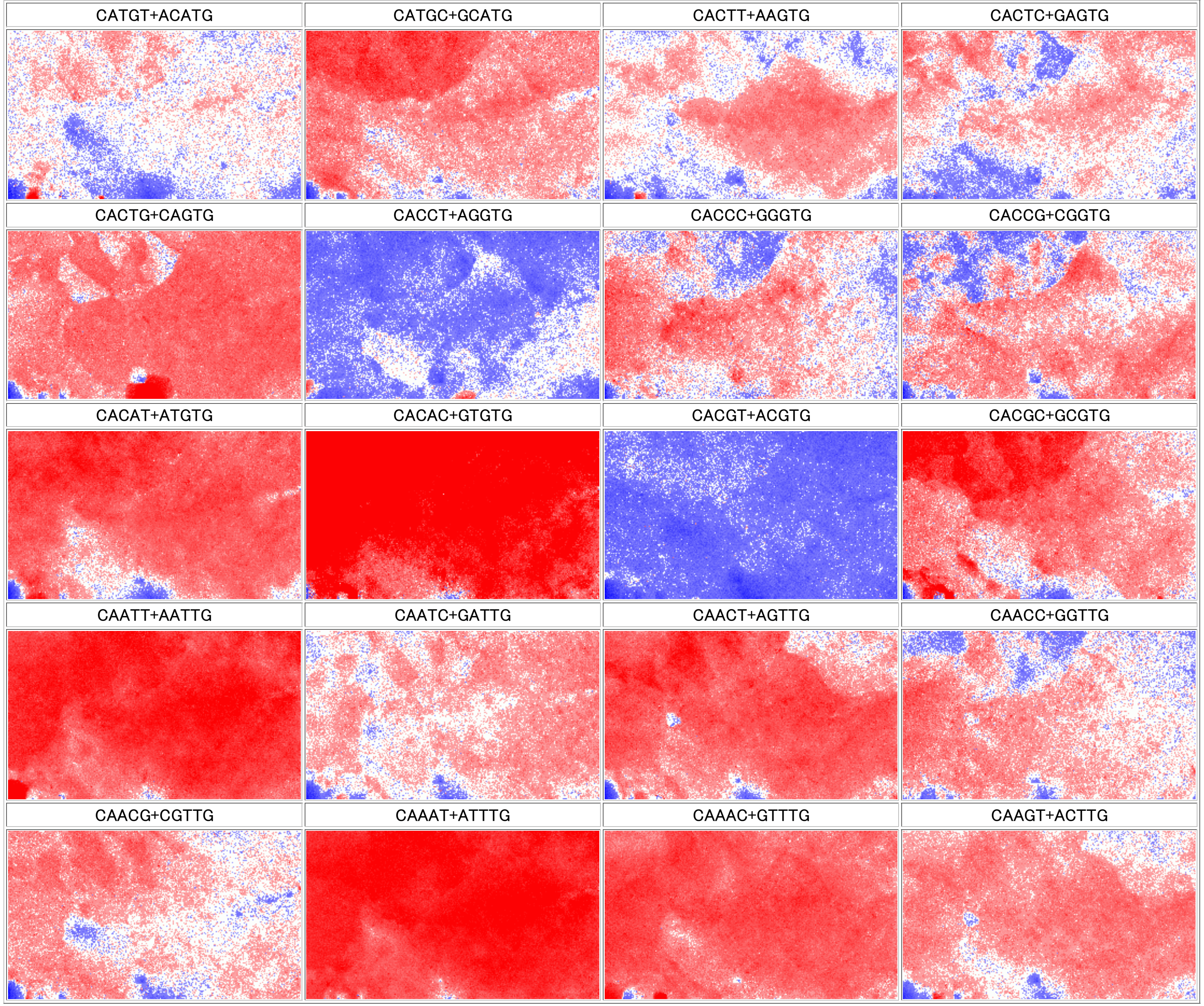

Supplementary Figure S3

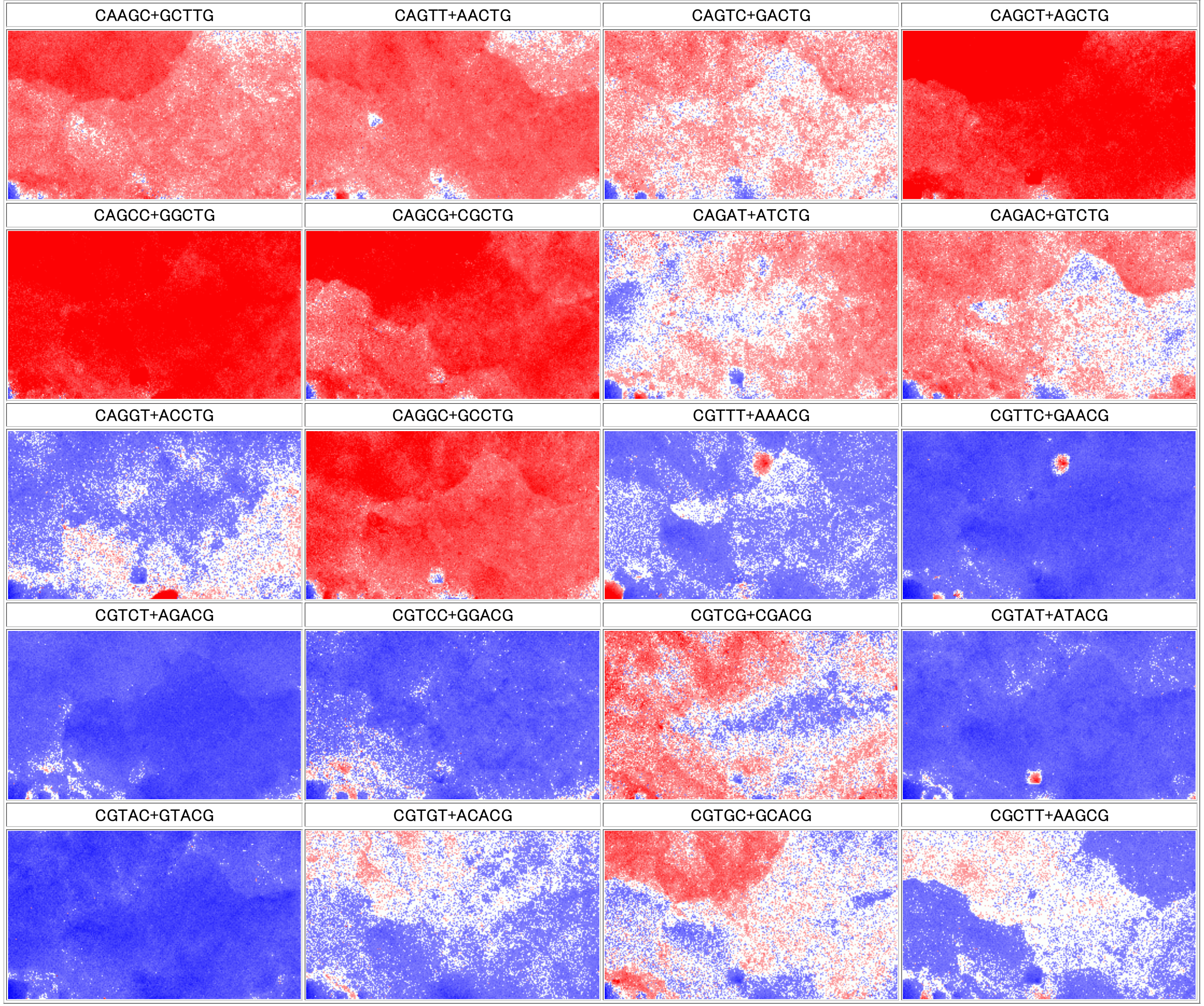

Supplementary Figure S3

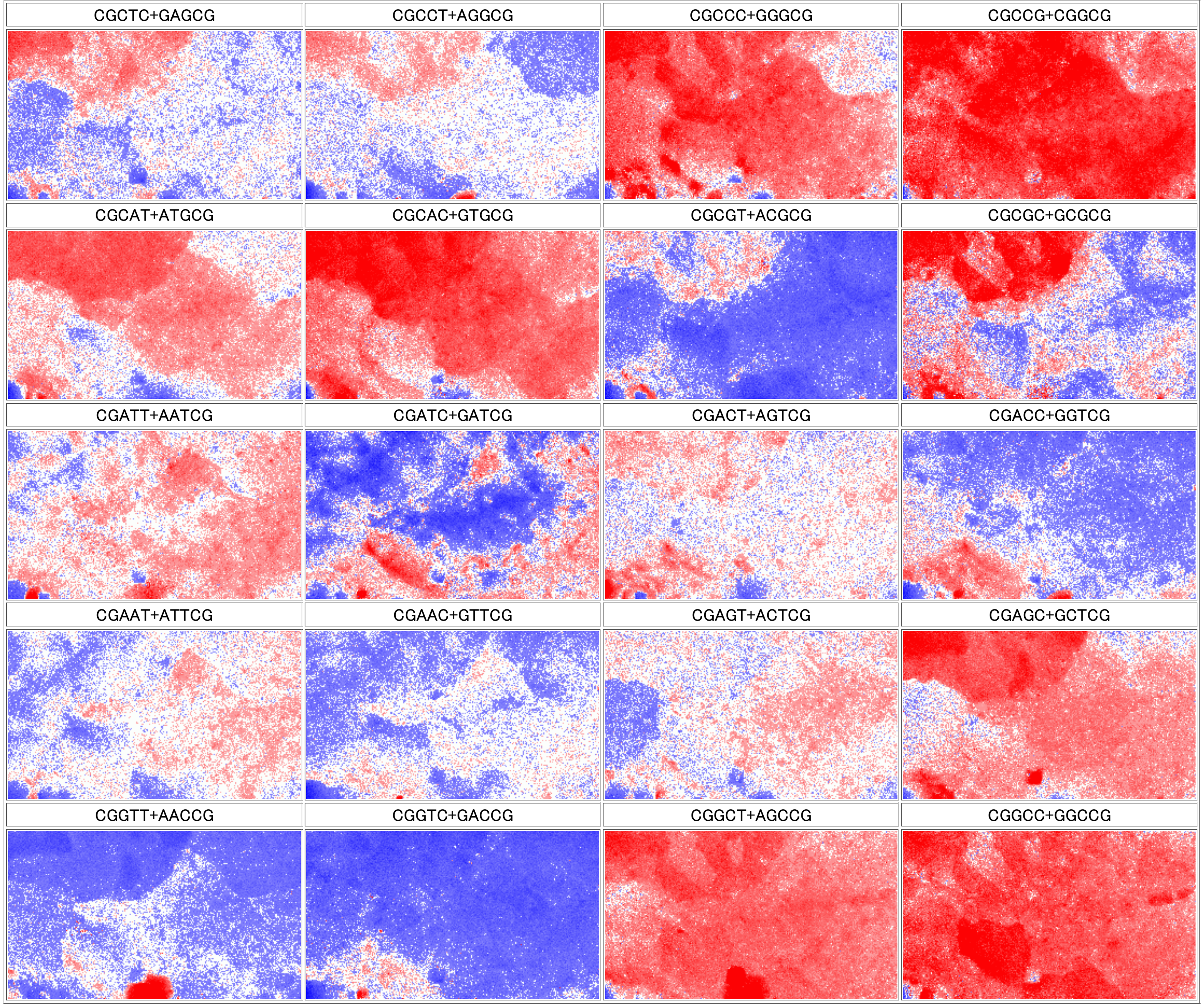

Supplementary Figure S3

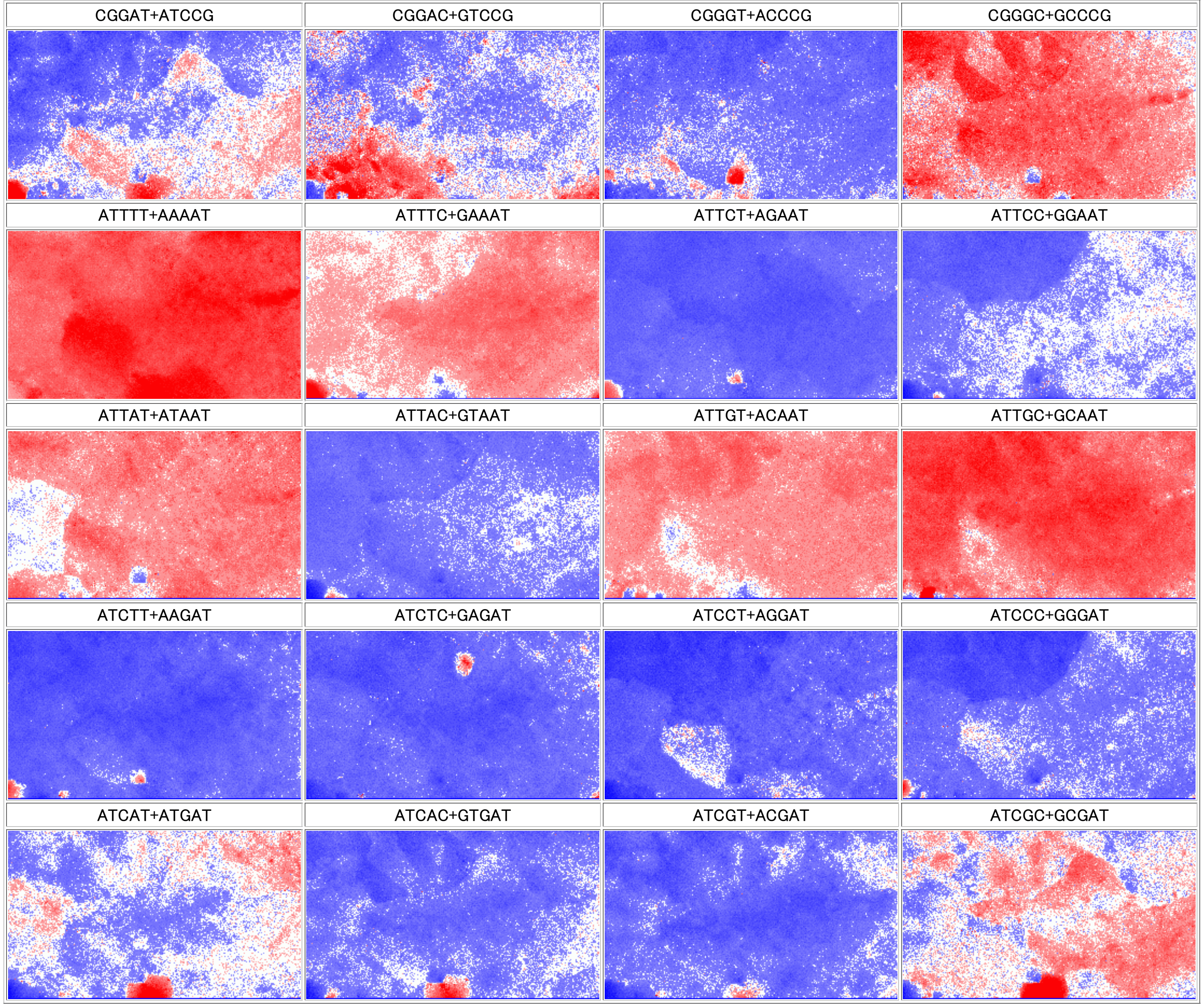

Supplementary Figure S3

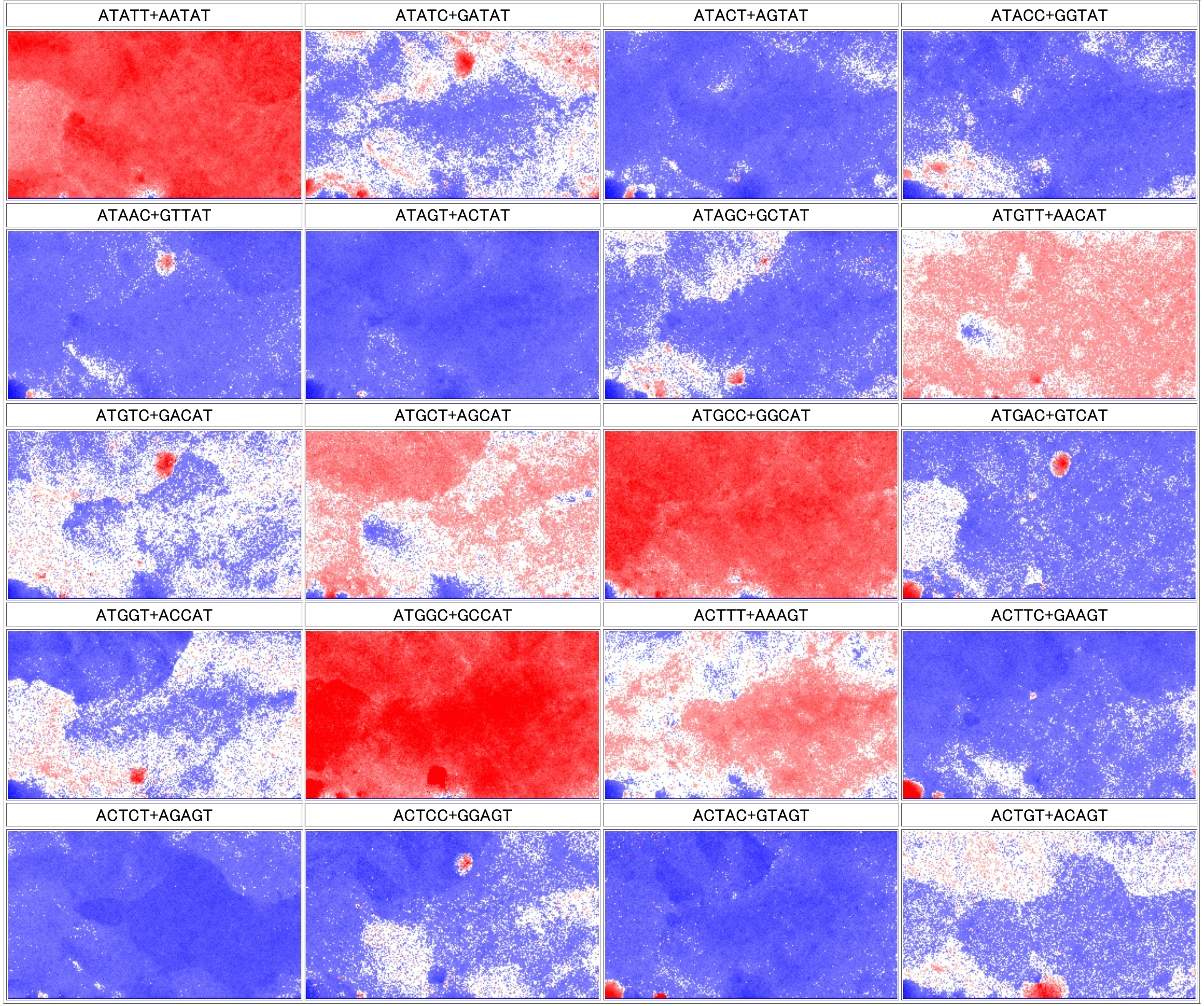

Supplementary Figure S3

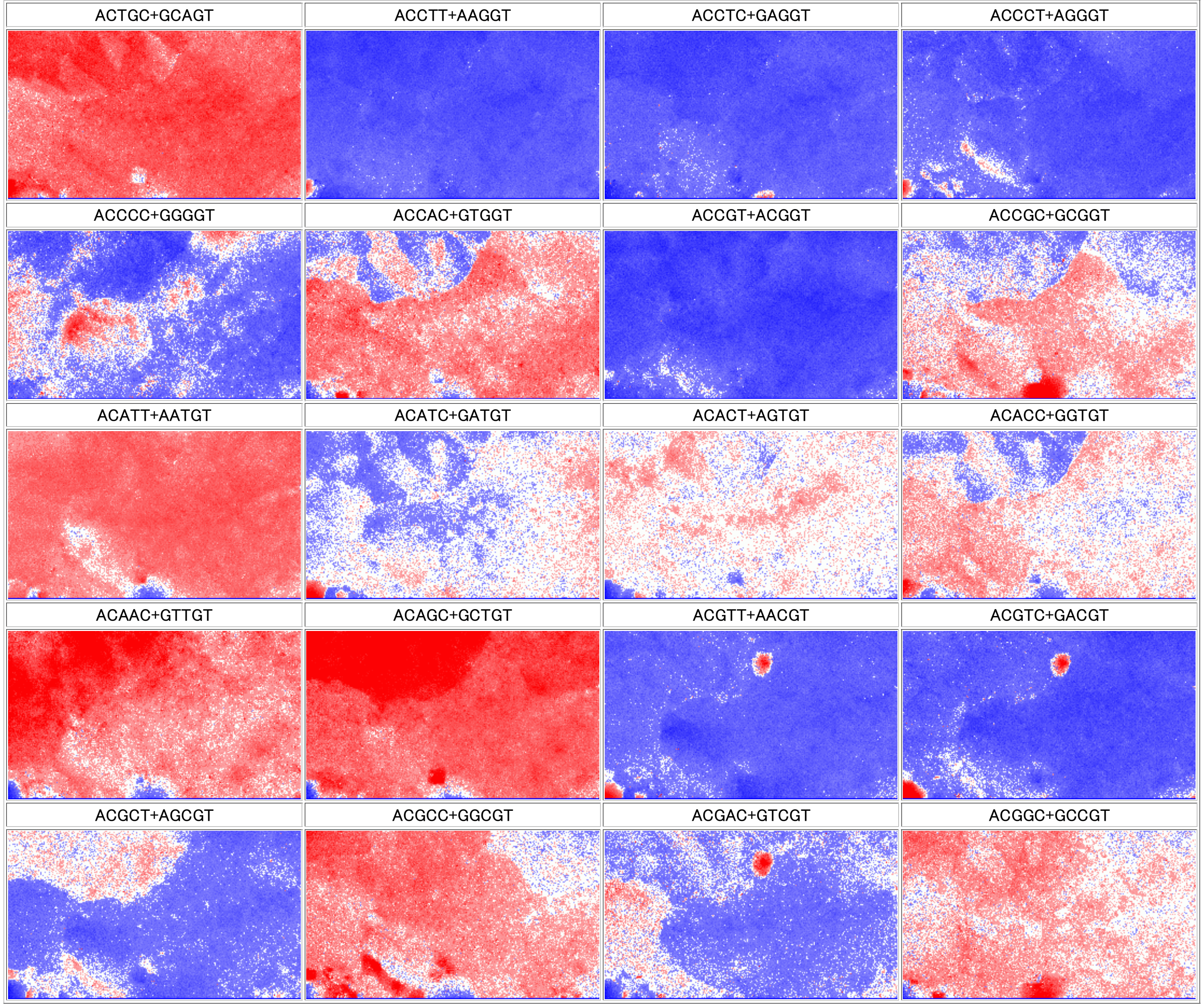

Supplementary Figure S3

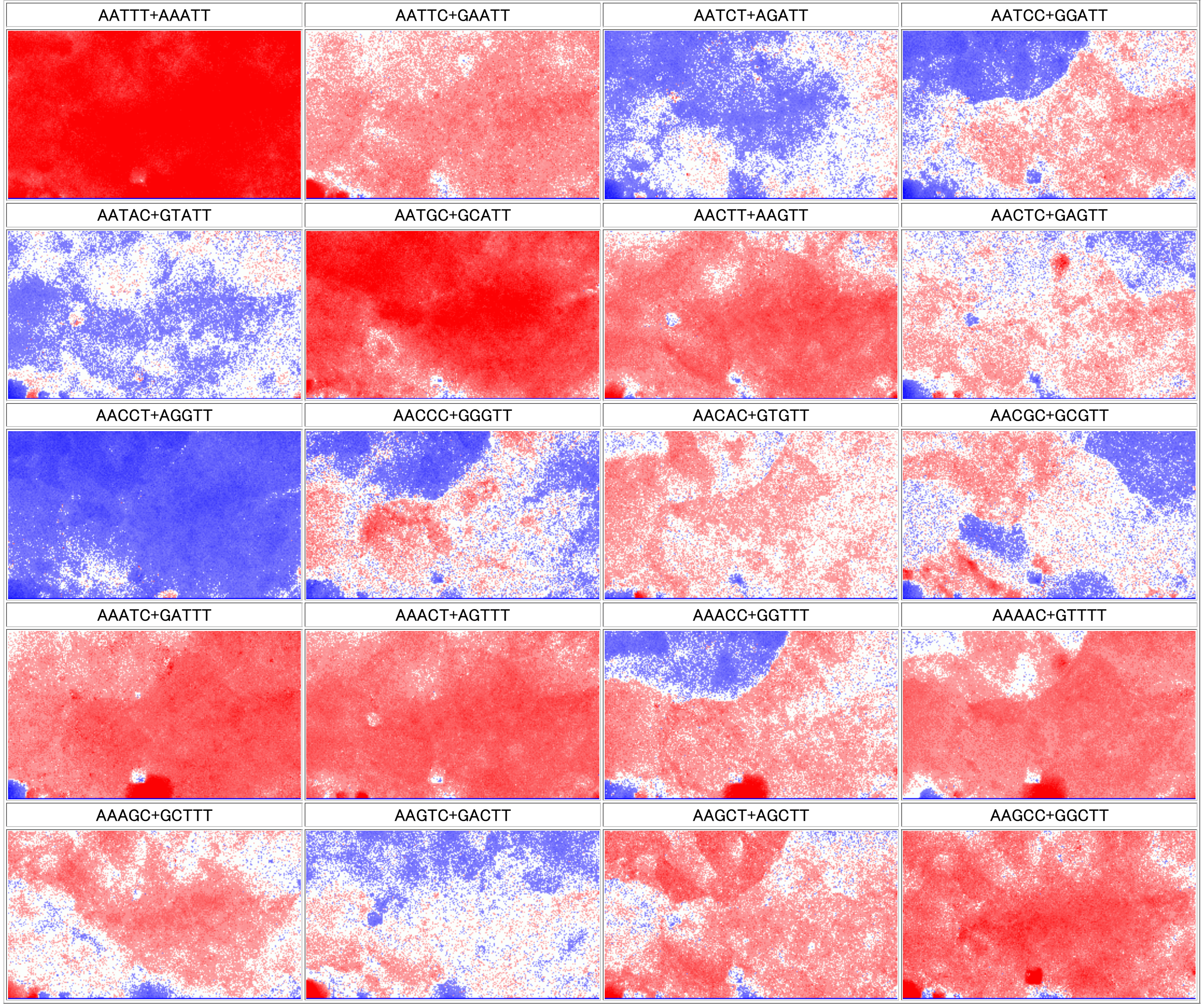

Supplementary Figure S3

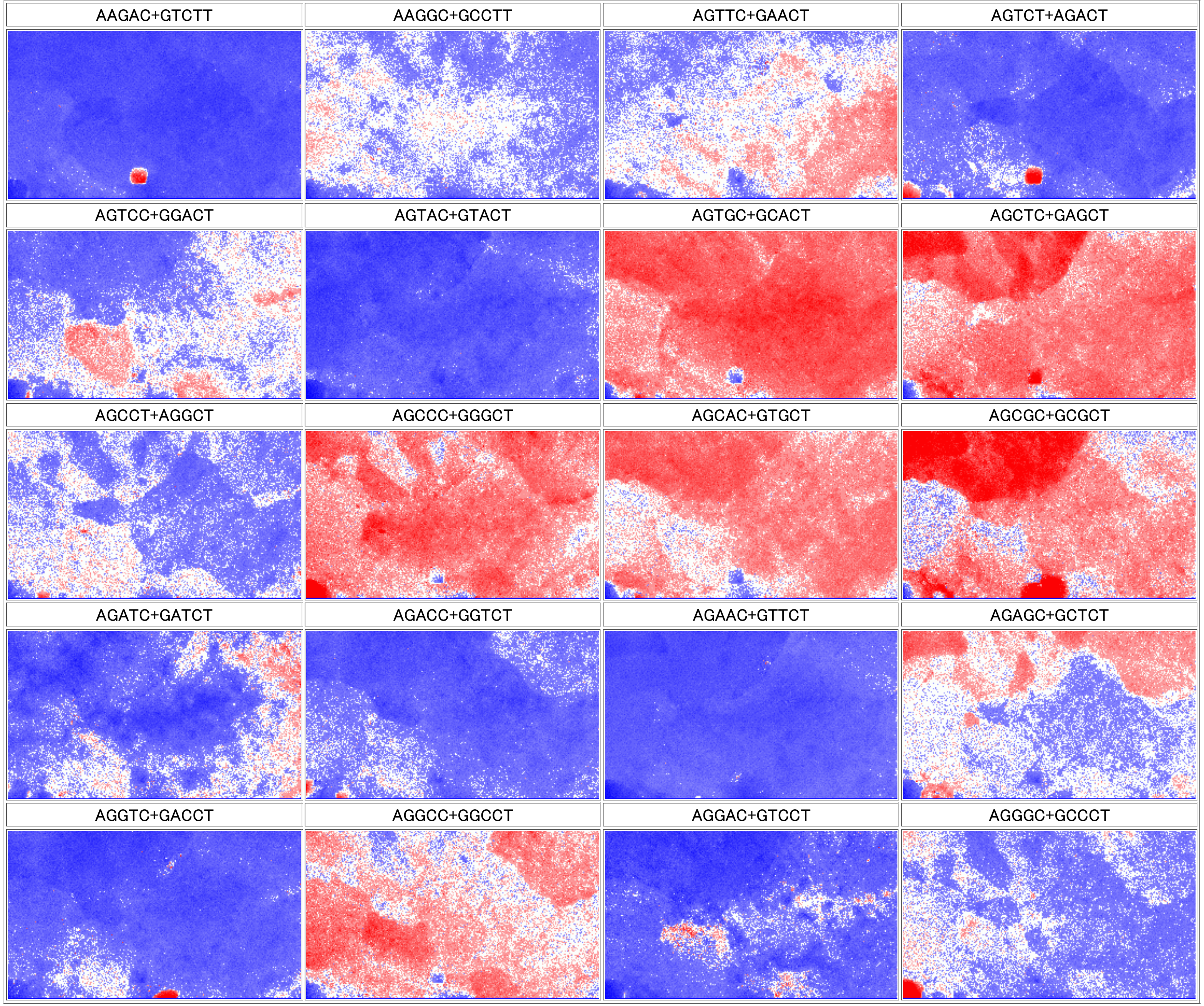

Supplementary Figure S3

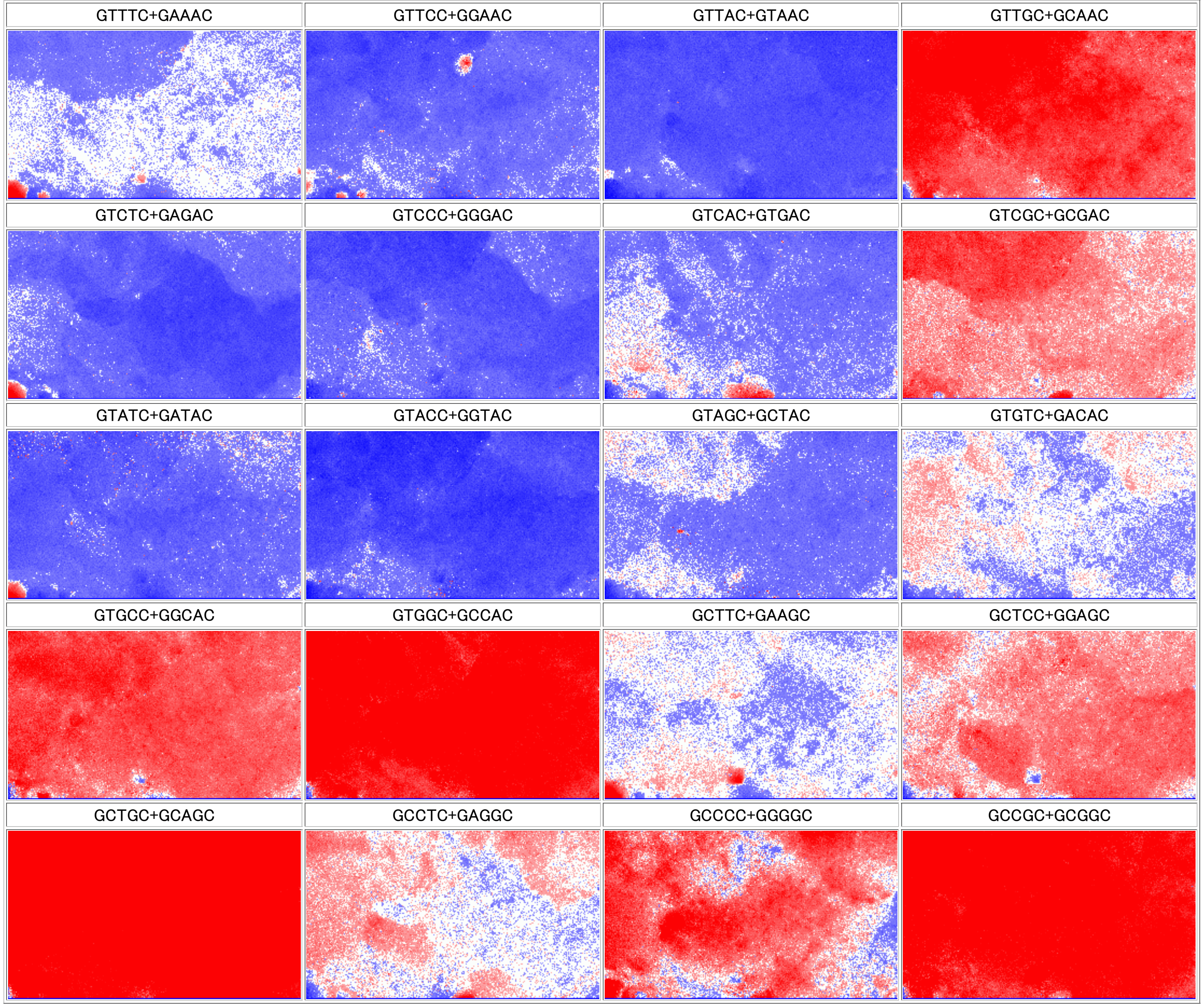

Supplementary Figure S3

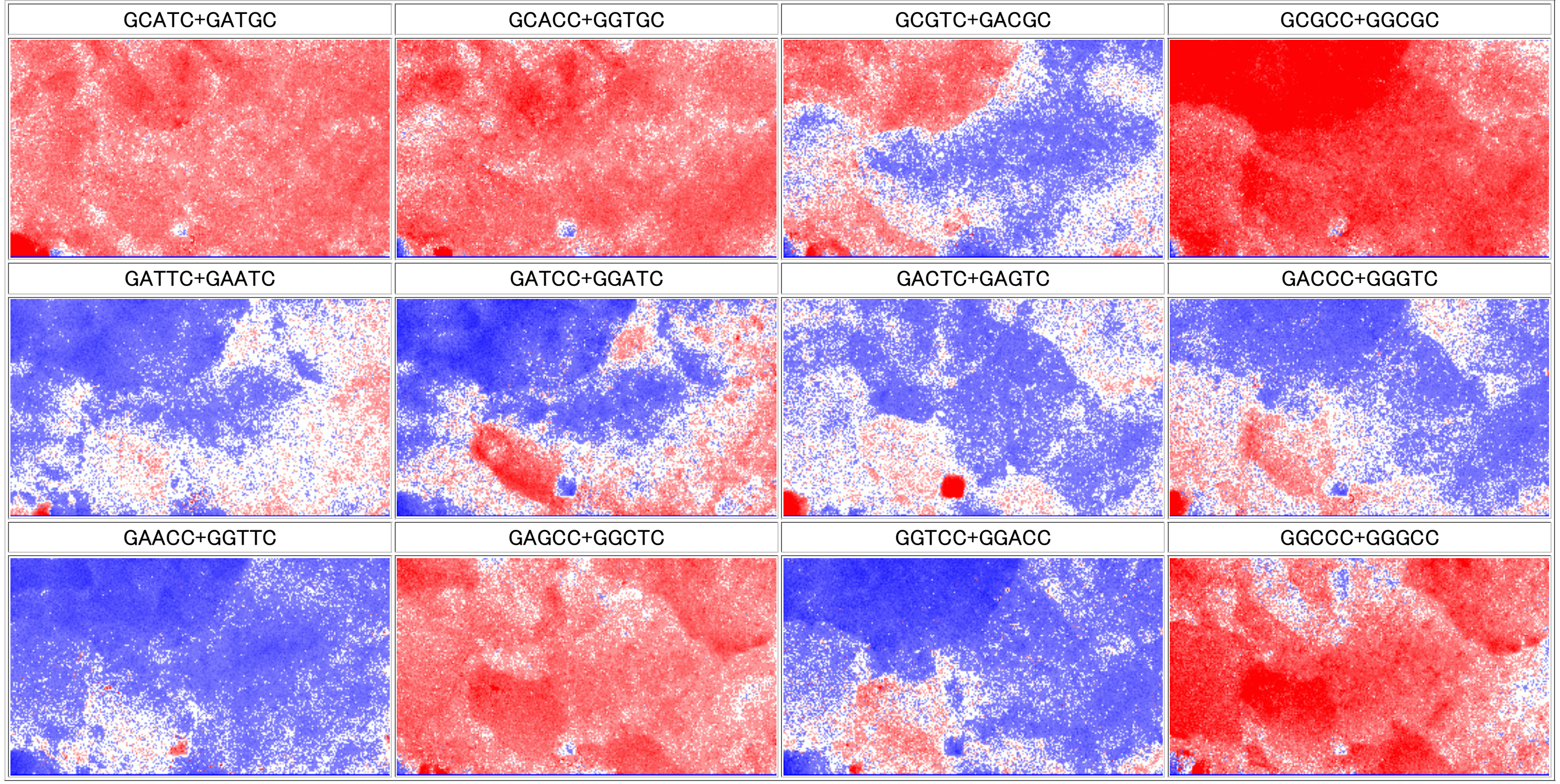

Supplementary Figure S3
